# Improved reference genome of *Aedes aegypti* informs arbovirus vector control

**DOI:** 10.1038/s41586-018-0692-z

**Published:** 2018-11-14

**Authors:** Benjamin J. Matthews, Olga Dudchenko, Sarah B. Kingan, Sergey Koren, Igor Antoshechkin, Jacob E. Crawford, William J. Glassford, Margaret Herre, Seth N. Redmond, Noah H. Rose, Gareth D. Weedall, Yang Wu, Sanjit S. Batra, Carlos A. Brito-Sierra, Steven D. Buckingham, Corey L. Campbell, Saki Chan, Eric Cox, Benjamin R. Evans, Thanyalak Fansiri, Igor Filipović, Albin Fontaine, Andrea Gloria-Soria, Richard Hall, Vinita S. Joardar, Andrew K. Jones, Raissa G. G. Kay, Vamsi K. Kodali, Joyce Lee, Gareth J. Lycett, Sara N. Mitchell, Jill Muehling, Michael R. Murphy, Arina D. Omer, Frederick A. Partridge, Paul Peluso, Aviva Presser Aiden, Vidya Ramasamy, Gordana Rašić, Sourav Roy, Karla Saavedra-Rodriguez, Shruti Sharan, Atashi Sharma, Melissa Laird Smith, Joe Turner, Allison M. Weakley, Zhilei Zhao, Omar S. Akbari, William C. Black, Han Cao, Alistair C. Darby, Catherine A. Hill, J. Spencer Johnston, Terence D. Murphy, Alexander S. Raikhel, David B. Sattelle, Igor V. Sharakhov, Bradley J. White, Li Zhao, Erez Lieberman Aiden, Richard S. Mann, Louis Lambrechts, Jeffrey R. Powell, Maria V. Sharakhova, Zhijian Tu, Hugh M. Robertson, Carolyn S. McBride, Alex R. Hastie, Jonas Korlach, Daniel E. Neafsey, Adam M. Phillippy, Leslie B. Vosshall

**Affiliations:** 1 Laboratory of Neurogenetics and Behavior, The Rockefeller University, New York, NY USA; 2 Howard Hughes Medical Institute, New York, NY USA; 3 Kavli Neural Systems Institute, New York, NY USA; 4 The Center for Genome Architecture, Baylor College of Medicine, Houston, TX USA; 5 Department of Molecular and Human Genetics, Baylor College of Medicine, Houston, TX USA; 6 Department of Computer Science, Rice University, Houston, TX USA; 7 Center for Theoretical and Biological Physics, Rice University, Houston, TX USA; 8 Pacific Biosciences, Menlo Park, CA USA; 9 National Human Genome Research Institute, National Institutes of Health, Bethesda, MD USA; 10 Division of Biology and Biological Engineering, California Institute of Technology, Pasadena, CA USA; 11 Verily Life Sciences, South San Francisco, CA USA; 12 Mortimer B. Zuckerman Mind Brain Behavior Institute, Department of Biochemistry and Molecular Biophysics, Columbia University, New York, NY USA; 13 Broad Institute of MIT and Harvard, Cambridge, MA USA; 14 Department of Immunology and Infectious Disease, Harvard T. H. Chan School of Public Health, Boston, MA USA; 15 Department of Ecology and Evolutionary Biology, Princeton University, Princeton, NJ USA; 16 Princeton Neuroscience Institute, Princeton University, Princeton, NJ USA; 17 Vector Biology Department, Liverpool School of Tropical Medicine, Liverpool, UK; 18 Liverpool John Moores University, Liverpool, UK; 19 Department of Pathogen Biology, School of Public Health, Southern Medical University, Guangzhou, China; 20 Department of Biochemistry, Virginia Tech, Blacksburg, VA USA; 21 Fralin Life Science Institute, Virginia Tech, Blacksburg, VA USA; 22 Department of Entomology, Purdue University, West Lafayette, IN USA; 23 Purdue Institute for Inflammation, Immunology and Infectious Disease, Purdue University, West Lafayette, IN USA; 24 Centre for Respiratory Biology, UCL Respiratory, University College London, London, UK; 25 Department of Microbiology, Immunology and Pathology, Colorado State University, Fort Collins, CO USA; 26 Bionano Genomics, San Diego, CA USA; 27 National Center for Biotechnology Information, National Library of Medicine, National Institutes of Health, Bethesda, MD USA; 28 Department of Ecology and Evolutionary Biology, Yale University, New Haven, CT USA; 29 Vector Biology and Control Section, Department of Entomology, Armed Forces Research Institute of Medical Sciences (AFRIMS), Bangkok, Thailand; 30 Mosquito Control Laboratory, QIMR Berghofer Medical Research Institute, Brisbane, Queensland Australia; 31 Insect-Virus Interactions Group, Department of Genomes and Genetics, Institut Pasteur, Paris, France; 32 Unité de Parasitologie et Entomologie, Département des Maladies Infectieuses, Institut de Recherche Biomédicale des Armées, Marseille, France; 33 Centre National de la Recherche Scientifique, Unité Mixte de Recherche 2000, Paris, France; 34 Aix Marseille Université, IRD, AP-HM, SSA, UMR Vecteurs – Infections Tropicales et Méditerranéennes (VITROME), IHU - Méditerranée Infection, Marseille, France; 35 The Connecticut Agricultural Experiment Station, New Haven, CT USA; 36 Department of Biological and Medical Sciences, Faculty of Health and Life Sciences, Oxford Brookes University, Oxford, UK; 37 Department of Entomology, University of California Riverside, Riverside, CA USA; 38 Department of Bioengineering, Rice University, Houston, TX USA; 39 Department of Pediatrics, Texas Children’s Hospital, Houston, TX USA; 40 Department of Entomology, Center for Disease Vector Research and Institute for Integrative Genome Biology, University of California, Riverside, CA USA; 41 Department of Entomology, Virginia Tech, Blacksburg, VA USA; 42 Institute of Integrative Biology, University of Liverpool, Liverpool, UK; 43 Division of Biological Sciences, University of California, San Diego, La Jolla, CA USA; 44 Tata Institute for Genetics and Society, University of California, San Diego, La Jolla, CA USA; 45 Department of Entomology, Texas A&M University, College Station, TX USA; 46 Laboratory of Ecology, Genetics and Environmental Protection, Tomsk State University, Tomsk, Russia; 47 Laboratory of Evolutionary Genetics and Genomics, The Rockefeller University, New York, NY USA; 48 Department of Entomology, University of Illinois at Urbana-Champaign, Urbana, IL USA

**Keywords:** Mosquito, Primary Contigs, Predicted Transcription Start Site, Aegypti Genome, Nano Bio, Genome, Population genetics

## Abstract

Female *Aedes aegypti* mosquitoes infect more than 400 million people each year with dangerous viral pathogens including dengue, yellow fever, Zika and chikungunya. Progress in understanding the biology of mosquitoes and developing the tools to fight them has been slowed by the lack of a high-quality genome assembly. Here we combine diverse technologies to produce the markedly improved, fully re-annotated AaegL5 genome assembly, and demonstrate how it accelerates mosquito science. We anchored physical and cytogenetic maps, doubled the number of known chemosensory ionotropic receptors that guide mosquitoes to human hosts and egg-laying sites, provided further insight into the size and composition of the sex-determining M locus, and revealed copy-number variation among glutathione *S*-transferase genes that are important for insecticide resistance. Using high-resolution quantitative trait locus and population genomic analyses, we mapped new candidates for dengue vector competence and insecticide resistance. AaegL5 will catalyse new biological insights and intervention strategies to fight this deadly disease vector.

## Abstract (web summary)

An improved, fully re-annotated *Aedes aegypti* genome assembly (AaegL5) provides insights into the sex-determining M locus, chemosensory systems that help mosquitoes to hunt humans and loci involved in insecticide resistance and will help to generate intervention strategies to fight this deadly disease vector.

## Main

**Fig. 1:**
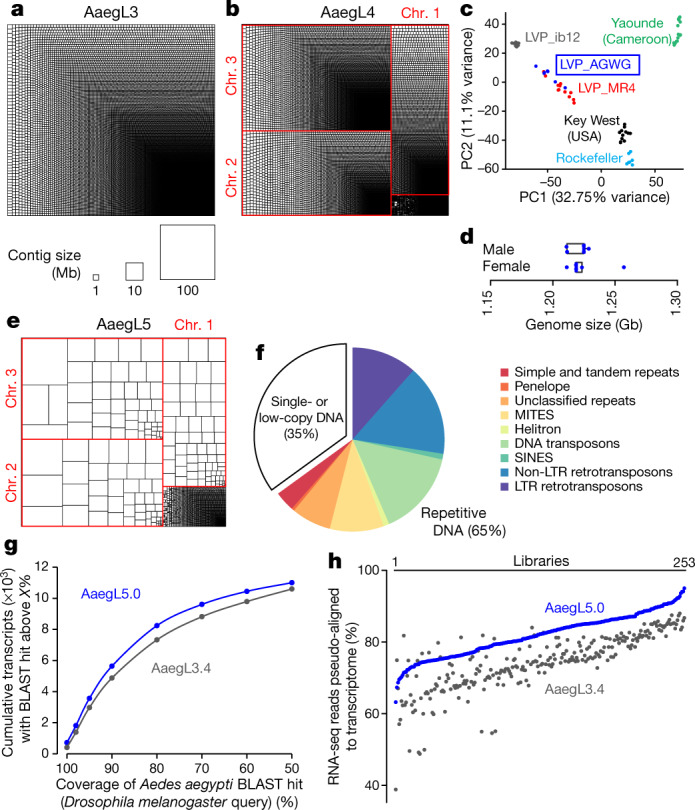
AaegL5 assembly statistics and annotation. **a**, **b**, Treemap of AaegL3 (**a**) and AaegL4 (**b**) contigs scaled by length. **c**, Principal component analysis of allelic variation of the indicated strains at 11,229 SNP loci. *n* = 7 per genotype **d**, Flow cytometry analysis of LVP_AGWG genome size. *n* = 5 per sex. Box plot: median is indicated by the blue line; boxes show first to third quartiles, whiskers are the 1.5× interquartile interval (Extended Data Fig. 1b). **e**, Treemap of AaegL5 contigs scaled by length. **f**, Genome composition (Supplementary Data 2, 3). **g**, Gene set alignment BLASTp coverage is compared between AaegL3.4 and AaegL5.0, with *D. melanogaster* protein queries. **h**, Alignment of 253 RNA-seq libraries to AaegL3.4 and AaegL5.0 gene set annotations (Supplementary Data 4–9). LTR, long terminal repeat retrotransposon; MITES, miniature inverted-repeat transposable elements; SINES, short interspersed nuclear elements.

**Extended Data Fig. 1:**
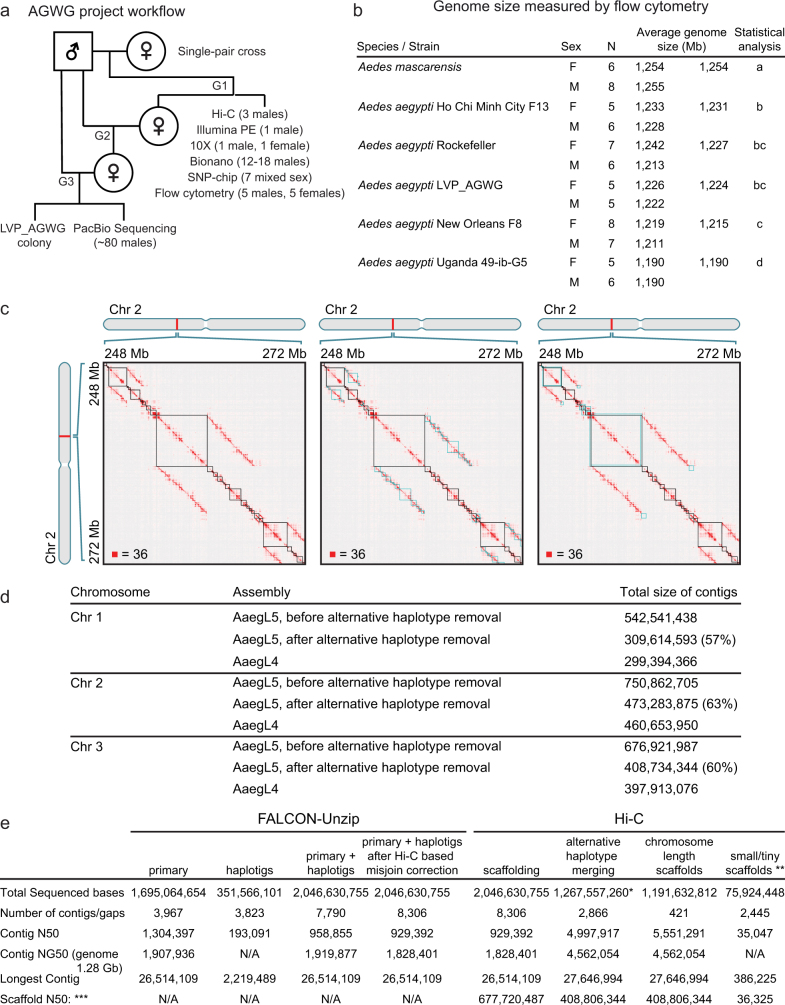
Project flowchart, measured genome size and assembly process. **a**, Flowchart of LVP_AGWG strain inbreeding, data collection and experimental design of the AaegL5 assembly process. **b**, Estimated average 1C genome size for each strain for five *Ae. aegypti* strains and *Ae. mascarensis*, the sister taxon of *Ae. aegypti*, for which the genome size has not previously been measured. There were no significant differences between the sexes within and between the species and strains analysed (*P* > 0.2). Significant differences between strains were determined using Proc GLM in SAS with both a Tukey and a Scheffé option with the same outcome. Data labelled with different letters are significantly different (*P* < 0.01). **c**, Combining Hi-C maps with 2D annotations enabled efficient review of sequences identified as alternative haplotypes by sequence alignment. The figure depicts a roughly 24 Mb × 24 Mb fragment of a contact map generated by aligning a Hi-C dataset to an intermediate genome assembly generated during the process of creating AaegL5. This intermediate assembly was a sequence comprising error-corrected, ordered and oriented FALCON-Unzip contigs. The intensity of each pixel in the contact map correlates with how often pairs of loci co-locate in the nucleus. Maximum intensity is indicated in the lower left of each panel. These maps include reads that do not align uniquely (reads with zero mapping quality); such alignments are randomly assigned to one of the possible genomic locations. Three panels show three types of annotations that are overlaid on top of the contact map. Left, FALCON-Unzip contig boundaries are highlighted as black squares along the diagonal. Notably, large linear features appear above and below the diagonal. These are the result of sequence overlap among contigs, which can indicate the presence of undercollapsed heterozygosity in the contig set. Because reads that do not map uniquely are randomly assigned during the alignment step, Hi-C reads derived from a contig will sometimes be aligned to an overlapping contig. When this happens, the Hi-C read pair may contribute to the formation of a linear feature above and below the diagonal. Therefore, the linear stretches of enriched contact frequency parallel to the diagonal are brought about by the random assignment procedure, and can facilitate the detection of pairs of overlapping contigs. Note that, when the overlap between contigs is owing to undercollapsed heterozygosity, both contigs will exhibit similar long-range contact patterns. This aspect of Hi-C data also provides evidence for the presence of undercollapsed heterozygosity. Centre, LASTZ-alignment-based annotations for fully redundant contigs. The squares shown in blue are obtained by taking diagonal contig boundary annotations (in black) and shifting them up (respectively, left) when drawing above (respectively, below) the diagonal so that the overlapping sequences are horizontally (respectively, vertically) aligned. Note that, as expected, the squares typically span linear, off-diagonal features in the Hi-C data. When one contig is entirely contained in another contig, the redundant contig does not contribute sequence to the merged chromosome-length scaffolds. Right, LASTZ-alignment-based annotations for partially redundant contigs. Again, the squares shown in blue are obtained by taking diagonal contig boundary annotations (in black) and shifting them up and left. The overlaps shown in this panel correspond to contigs that only partially overlap in sequence with other contigs. Consequently, some of their sequence is incorporated in the final fasta. **d**, Comparison of chromosome lengths between AaegL4 and AaegL5. Numbers are given before post-Hi-C polishing and gap closing. **e**, Step-wise assembly statistics for Hi-C scaffolding, alternative haplotype removal and annotation. *Removed length, 779,073,495 bp. **The definition of scaffold groups can be found in a previously published study^4^. ***Gaps between contigs were set to 500 bp for calculating scaffold statistics. N/A, not applicable.

**Extended Data Fig. 2:**
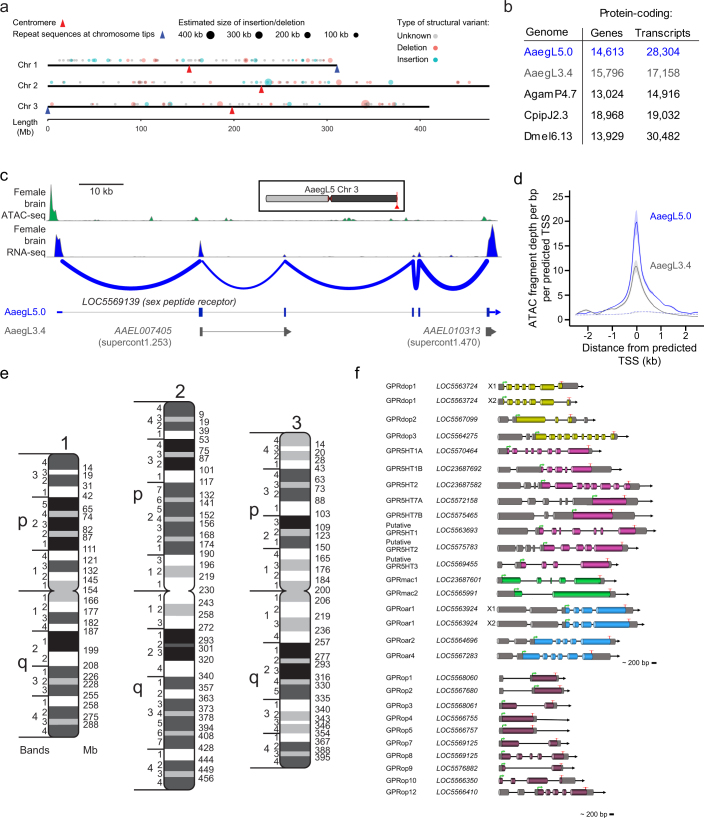
Remaining assembly gaps, summary of geneset annotation improvement, chromatin accessibility analysis, physical genome map and gene strucures of biogenic amine-binding receptors and opsins in AaegL5. **a**, Representation of structural variants identified at assembly gaps by alignment of Bionano optical maps. The estimated size of an insertion (blue) or deletion (red) relative to the reference is represented by the size of the circle. When the size or type of structural variants could not be determined or did not agree between the two optical maps, the location of the assembly gap is plotted in grey. Approximate locations of the centromeres (red triangles) and telomere-associated repeat sequences (blue triangles) are indicated. Raw data are available as Supplementary Data 1. **b**, Comparison of protein-coding genes and transcripts in AaegL5.0 (NCBI RefSeq Release 101) and gene set annotations from *An. gambiae* (Agam), *Culex pipiens* (Cpip) and *D. melanogaster* (Dmel). **c**, *Sex peptide receptor *structure in AaegL3.4 and AaegL5.0, and female brain RNA-seq and ATAC-seq reads aligned to AaegL5. Blue lines on the RNA-seq track indicate splice junctions, with the number of reads spanning a junction represented by line thickness. Exons are represented by tall filled boxes and introns by lines. Arrowheads indicate gene orientation. **d**, Average read profiles across promoter regions, defined as the transcription start site (TSS) ±2.5 kb. Solid lines represent Tn5-treated native chromatin using the ATAC-seq protocol (*n* = 4), dotted lines represent Tn5-treated naked genomic DNA (*n* = 1). Shaded regions represent s.d. **e**, A physical genome map was developed by localizing 500 BAC clones to chromosomes using FISH. For the development of a final chromosome map for the AaegL5 assembly, we assigned the coordinates of each outmost BAC clone within a band (Supplementary Data 12) to the boundaries between bands. The final resolution of this map varies on average between 5 and 10 Mb because of the differences in BAC mapping density in different regions of chromosomes. **f**, Schematic of predicted gene structures of the *Ae. aegypti* biogenic amine-binding receptors and opsins. Exons, cylindrical bars; introns, black lines; dopamine receptors, yellow bars; serotonin receptors, magenta bars; muscarinic acetylcholine receptors, green bars; octopamine receptors, blue bars; opsins, dark purple bars; predicted 3′ and 5′ non-coding sequence (dark shading). The ‘unclassified receptor’ *GPRnna19* is not shown. Details on gene models compared to previous annotations and the predicted amino acid sequences of each gene are available in Supplementary Data 14–16.

An accurate and complete genome assembly is required to understand the unique aspects of mosquito biology and to develop control strategies to reduce their capacity to spread pathogens^1^. The *Ae. aegypti* genome is large (approximately 1.25 Gb) and highly repetitive, and a 2007 genome project (AaegL3)^2^ was unable to produce a contiguous genome fully anchored to a physical chromosome map^3^ (Fig. 1a). A more recent assembly, AaegL4^4^, produced chromosome-length scaffolds that made it possible to detect larger-scale syntenic genomic regions in other species but suffered from short contigs (contig N50, 84 kb, meaning that half of the assembly is found on contigs >84 kb) and a correspondingly large number of gaps (31,018; Fig. 1b). Taking advantage of rapid advances in sequencing and assembly technology in the last decade, we used long-read Pacific Biosciences sequencing and Hi-C (a high-throughput sequencing method based on chromosome conformation capture) scaffolding to produce a new reference genome (AaegL5) that is highly contiguous, with a decrease of 93% in the number of contigs, and anchored end-to-end to the three *Ae. aegypti* chromosomes (Fig. 1 and Extended Data Figs. 1, 2). Using optical mapping and linked-read sequencing, we validated the local structure and predicted structural variants between haplotypes. We generated an improved gene set annotation (AaegL5.0), as assessed by a mean increase in RNA-sequencing (RNA-seq) read alignment of 12%, connections between many gene models that were previously split across multiple contigs, and a roughly twofold increase in the enrichment of assay for transposase-accessible chromatin using sequencing (ATAC-seq) alignments near predicted transcription start sites. We demonstrate the utility of AaegL5 and the AaegL5.0 annotation by investigating a number of scientific questions that could not be addressed with the previous genome annotations.

**Fig. 2:**
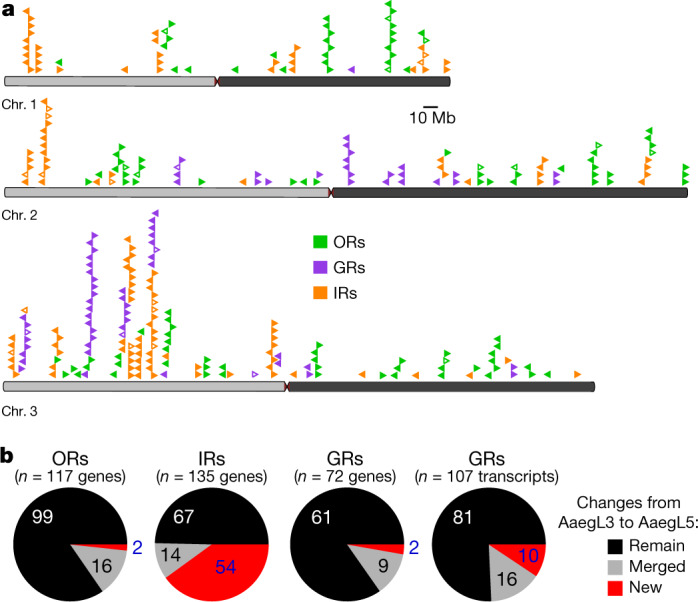
Chromosomal arrangement and increased number of chemosensory receptor genes. **a**, Location of predicted chemoreceptors (odorant receptors (ORs), gustatory receptors (GRs) and ionotropic receptors (IRs)) by chromosome in AaegL5. The blunt end of the arrowheads marks gene position and the arrow indicates orientation. Filled and open arrowheads represent intact genes and pseudogenes, respectively (Supplementary Data 17–20 and Extended Data Fig. 3). **b**, Chemosensory receptor annotations are compared between AaegL5 and AaegL3.

**Fig. 3:**
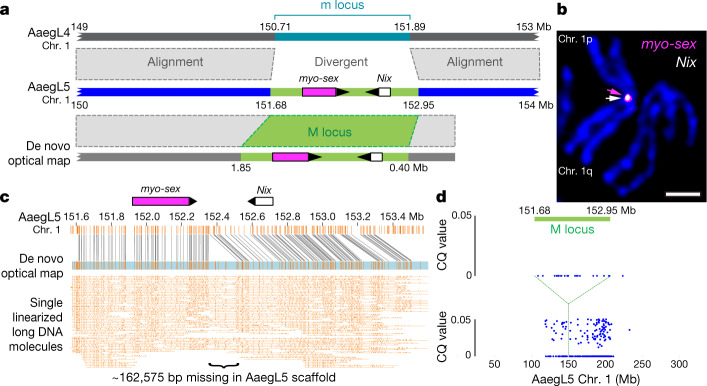
Application of AaegL5 to resolve the sex-determining locus. **a**, M locus structure indicating high alignment identity (grey-dashed boxes) and boundaries of *myo-sex* and *Nix* gene models (magenta and white boxes, arrowheads represent orientation). **b**, FISH of BAC clones containing *myo-sex* and *Nix*. Scale bar, 2 μm. Representative image of 10 samples. **c**, De novo optical map spanning the M locus and bridging the estimated 163-kb gap in the AaegL5 assembly. DNA molecules are cropped at the edges for clarity. **d**, Chromosome quotient (CQ) analysis of genomic DNA from male and female libraries aligned to AaegL5 chromosome 1. Each dot represents the CQ value of a repeat-masked 1-kb window with >20 reads aligned from male libraries.

**Fig. 4:**
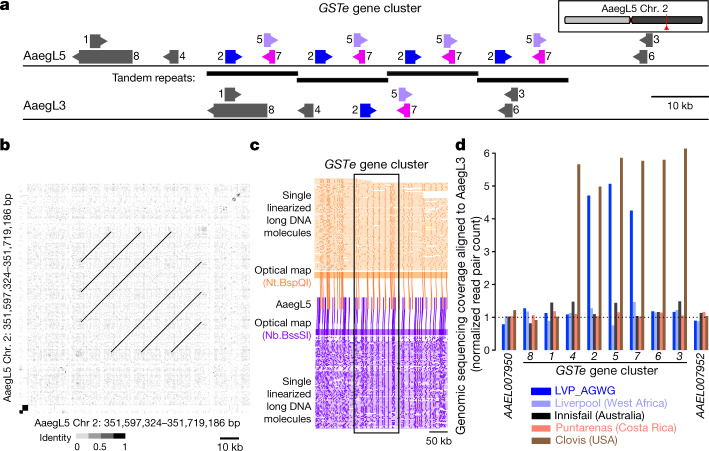
Copy-number variation in the glutathione *S*-transferase epsilon gene cluster. **a**, Glutathione *S*-transferase epsilon (*GSTe*) gene cluster structure in AaegL5 compared to AaegL3 (Supplementary Data 23). Arrowheads indicate gene orientation. **b**, Dot-plot alignment of AaegL5 *GSTe* region to itself. **c**, Optical mapping of DNA labelled with indicated enzymes. DNA molecules are cropped at the edges for clarity. **d**, Genomic sequencing coverage of AaegL3 *GSTe* genes (DNA read pairs mapped to each gene, normalized by gene length in kb) from one LVP_AGWG male and pooled mosquitoes from four other laboratory strains.

**Fig. 5:**
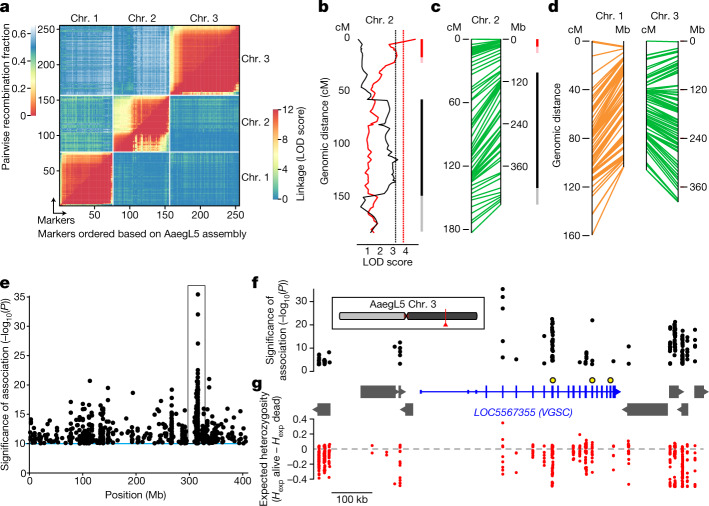
Using the AaegL5 genome for applied population genetics. **a**, Heat map of linkage based on pairwise recombination fractions for 255 RAD markers ordered by AaegL5 physical coordinates. **b**, Significant QTLs on chromosome 2 underly systemic DENV dissemination in midgut-infected mosquitoes (Extended Data Fig. 10a). Curves represent log of the odds ratio (LOD) scores obtained by interval mapping. Dotted vertical lines indicate genome-wide statistical significance thresholds (*α* = 0.05). Confidence intervals of significant QTLs: bright colour, 1.5-LOD interval; light colour, 2-LOD interval with generalist effects (black, across DENV serotypes and isolates) and DENV isolate-specific effects (red, indicative of genotype-by-genotype interactions). **c**, **d**, Synteny between linkage map (in cM) and physical map (in Mb) for chromosome 2 (**c**) and chromosomes 1 and 3 (**d**). The orange color of chromosome 1 denotes uncertainty in the cM estimates because of deviations in Mendelian ratios surrounding the M locus. **e**, Chromosome 3 SNPs significantly correlated with deltamethrin survival. **f**, **g**, Magnified and inverted view of box in **e**, centred on the new gene model of voltage-gated sodium channel (*VGSC*, transcript variant X3; the chromosomal position is indicated in red). **f**, Non-coding genes are omitted for clarity, and other genes indicated with grey boxes. *VGSC* exons are represented by tall boxes and untranslated regions by short boxes. Arrowheads indicate gene orientation. Non-synonymous *VGSC* SNPs are marked with larger black and yellow circles: V1016I = 315,983,763; F1534C = 315,939,224; V410L = 316,080,722. **g**, Difference in expected heterozygosity (*H*_exp_ alive − *H*_exp_ dead) for all SNPs.

This project used the Liverpool *Aedes* Genome Working Group (LVP_AGWG) strain, related to the AaegL3 Liverpool ib12 (LVP_ib12) assembly strain^2^ (Fig. 1c and Extended Data Fig. 1a). Using flow cytometry, we estimated that the genome size of LVP_AGWG is approximately 1.22 Gb (Fig. 1d and Extended Data Fig. 1b). To generate our primary assembly, we produced 166 Gb of Pacific Biosciences data (around 130× coverage for a 1.28-Gb genome) and assembled the genome using FALCON-Unzip^5^. This resulted in a total assembly length of 2.05 Gb (contig N50, 0.96 Mb; and NG50, 1.92 Mb, meaning half of the expected genome size found on contigs >1.92 Mb). FALCON-Unzip annotated the resulting contigs as either primary (3,967 contigs; N50, 1.30 Mb; NG50, 1.91 Mb) or haplotigs (3,823 contigs; N50, 193 kb), representing alternative haplotypes present in the approximately 80 male siblings pooled for sequencing (Table 1 and Extended Data Fig. 1e). The primary assembly was longer than expected for a haploid *Ae. aegypti* genome, as predicted by flow cytometry and prior assemblies, which was consistent with remaining alternative haplotypes that were too divergent to be automatically identified as primary and associated alternative haplotig pairs.

**Table 1:** Comparison of assembly statistics

	Genome assembly			
	AaegL3	AaegL4	AaegL5 FALCON-Unzip	AaegL5 (NCBI) FALCON-Unzip + Hi-C + polish
Total length (non-N bp)	1,310,092,987	1,254,548,160	1,695,064,654	1,278,709,169
Contig number	36,205	37,224	3,967	2,539
Contig N50 (bp)	82,618	84,074	1,304,397	11,758,062
Contig NG50 (bp)	85,043	81,911	1,907,936	11,758,062
Scaffold number	4,757	6,206	N/A	2,310
Scaffold N50 (bp)	1,547,048	404,248,146^a^	N/A	409,777,670^a^
GC content (%)	38.27	38.28	38.16	38.18
Alternative haplotypes (bp)	N/A	N/A	351,566,101	591,941,260
Alternative haplotypes (contigs)	N/A	N/A	3,823	4,224

N/A, not applicable.

^a^Scaffold N50 is the length of chromosome 3.

To generate a linear chromosome-scale reference genome assembly, we combined the primary contigs and haplotigs that were generated by FALCON-Unzip to create an assembly comprising 7,790 contigs. We used Hi-C to order and orient these contigs, correct misjoined sections and merge overlaps (Extended Data Fig. 1c–e). We set aside 359 contigs that were shorter than 20 kb and used the Hi-C data to identify 258 misjoined sections, resulting in 8,306 ordered and oriented contigs. This procedure revealed extensive sequence overlap among the contigs, consistent with the assembly of numerous alternative haplotypes. We developed a procedure to merge these alternative haplotypes, removing 5,440 gaps and boosting the contiguity (N50, 5.0 Mb; NG50, 4.6 Mb). This procedure placed 94% of sequenced (non-duplicated) bases onto three chromosome-length scaffolds that correspond to the three *Ae. aegypti* chromosomes. After scaffolding, we performed gap-filling and polishing using Pacific Biosciences reads. This removed 270 gaps and further increased the contiguity (N50, 11.8 Mb; NG50, 11.8 Mb), resulting in a final 1.279-Gb AaegL5 assembly and a complete mitochondrial genome (Fig. 1e and Table 1). We used Hi-C contact maps to estimate the position of the centromere with a resolution of around 5 Mb: chromosome 1, approximately 150–154 Mb; chromosome 2, around 227–232 Mb, chromosome 3, around 196–201 Mb. There are 229 remaining gaps in the primary assembly, including 173 on the three primary chromosomal scaffolds (Extended Data Fig. 2a and Supplementary Data 1). Analysis of near-universal single-copy orthologues using BUSCO^6^ revealed a slight increase in complete single-copy orthologues and a reduction in fragmented and missing genes compared to previous assemblies (see Supplementary Methods and Supplementary Discussion). AaegL5 is markedly more contiguous than AaegL3 and AaegL4 assemblies^2,4^ (Fig. 1a, b, e and Table 1). Using the TEfam, Repbase and de novo identified repeat databases, we found that 65% of AaegL5 was composed of transposable elements and other repetitive sequences (Fig. 1f and Supplementary Data 2, 3).

Complete and correct gene models are essential for studying all aspects of mosquito biology. We used the NCBI RefSeq annotation pipeline to produce annotation version 101 (AaegL5.0; Extended Data Fig. 2b) followed by manual curation of key gene families. AaegL5.0 formed the basis for a comprehensive quantification of transcript abundance in 253 sex-, tissue- and developmental stage-specific RNA-seq libraries (Supplementary Data 4–8). The AaegL5.0 gene set is considerably more complete and correct than previous versions. Many more genes have high protein coverage when compared to *Drosophila melanogaster* orthologues (915 more genes with >80% coverage, a 12.5% increase over AaegL3.4; Fig. 1g) and >12% more RNA-seq reads map to the AaegL5.0 gene set annotation than AaegL3.4 (Fig. 1h and Supplementary Data 9). In addition, 1,463 genes that were previously annotated separately as paralogues were collapsed into single gene models and 481 previously fragmented gene models were completed (Supplementary Data 10, 11). For example, *sex peptide receptor* is represented by a six-exon gene model in AaegL5.0 compared to two partial gene fragments on separate scaffolds in AaegL3.4 (Extended Data Fig. 2c). Genome-wide, we mapped a 1.8-fold higher number of ATAC-seq reads, known to co-localize with promoters and other *cis*-regulatory elements^7^, to predicted transcription start sites in AaegL5.0 compared to AaegL3.4, consistent with more complete gene models in AaegL5.0 (Extended Data Fig. 2d).

We next validated the base-level and structural accuracy of the AaegL5 assembly. We estimate the lower bound of base-level accuracy of the assembly to have a quality value of 34.75 (meaning that 99.9665% of bases are correct, see Supplementary Methods and Supplementary Discussion). To develop a fine-scale physical genome map based on AaegL5, we compared the assembly coordinates of 500 bacterial artificial chromosome (BAC) clones containing *Ae. aegypti* genomic DNA with physical mapping by fluorescence in situ hybridization (FISH) (Extended Data Fig. 2e and Supplementary Data 12). After removing repetitive BAC-end sequences and those with ambiguous FISH signals, 377 out of 387 (97.4%) of probes showed concordance between physical mapping and BAC-end alignment. The 10 remaining discordant signals were not supported by Bionano or 10X analysis, and therefore probably do not reflect misassemblies in AaegL5. The genome coverage of this physical map is 93.5%, compared to 45% of AaegL3^8^, and is one of the most complete genome maps across mosquito species^9,10^.

## Curation of multi-gene families

**Extended Data Fig. 3:**
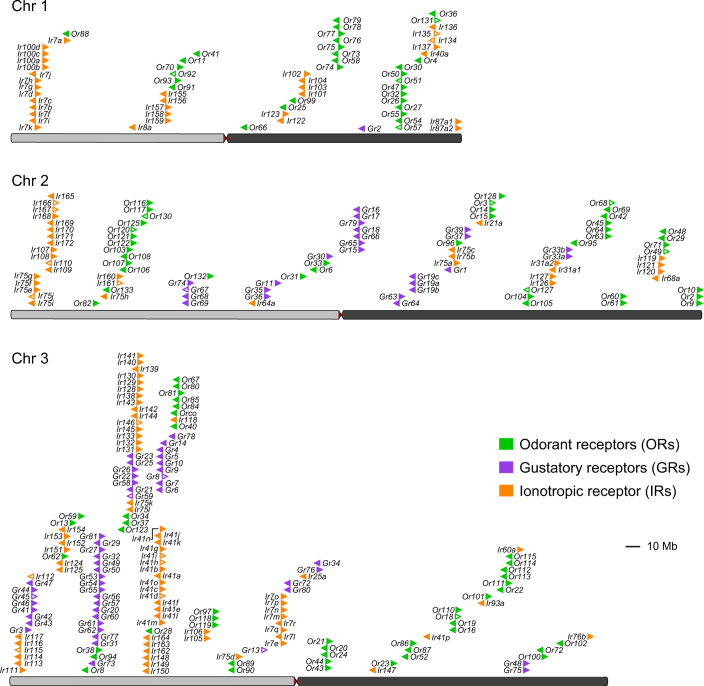
Chromosomal arrangement of chemosensory receptor genes. The location of predicted chemoreceptors (odorant receptors (ORs), gustatory receptors (GRs) and ionotropic receptors (IRs)) across all three chromosomes in AaegL5. The blunt end of each arrowhead plotted above each chromosome marks gene position and arrowhead indicates orientation. Filled and open arrowheads represent intact genes and pseudogenes, respectively (Supplementary Data 17–20). This figure is identical to Fig. 2a, but here includes gene names.

**Extended Data Fig. 4:**
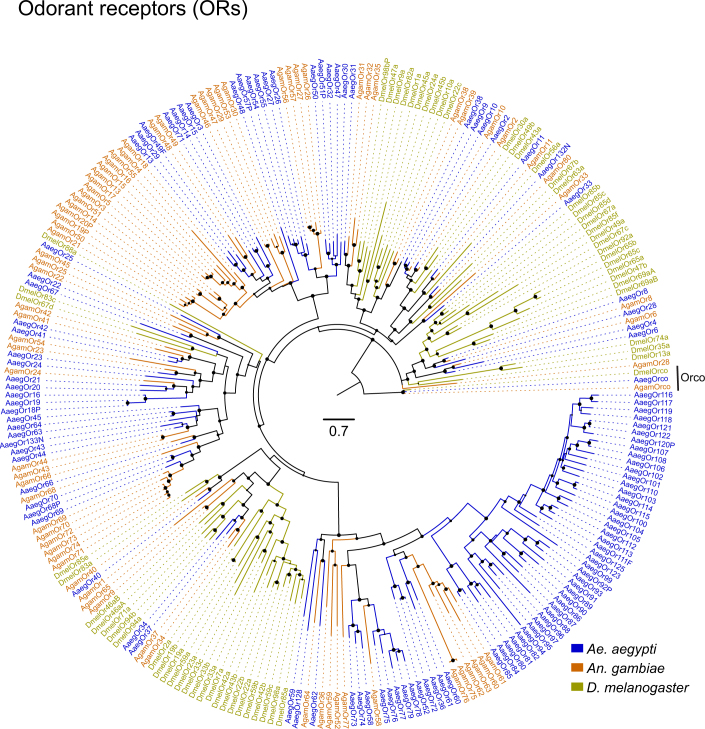
Phylogenetic tree of odorant receptor gene families from *Ae. aegypti*, *An. gambiae* and *D. melanogaster*. Maximum likelihood odorant receptor tree was rooted with Orco proteins, which are both highly conserved and basal within the odorant receptor family^89^. Support levels for nodes are indicated by the size of black circles—reflecting approximate likelihood ratio tests (aLRT values ranging from 0 to 1 from PhyML v.3.0 run with default parameters^90^). Suffixes after protein names are C, minor assembly correction; F, major assembly modification; N, new model; P, pseudogene. Scale bar, amino acid substitutions per site.

**Extended Data Fig. 5:**
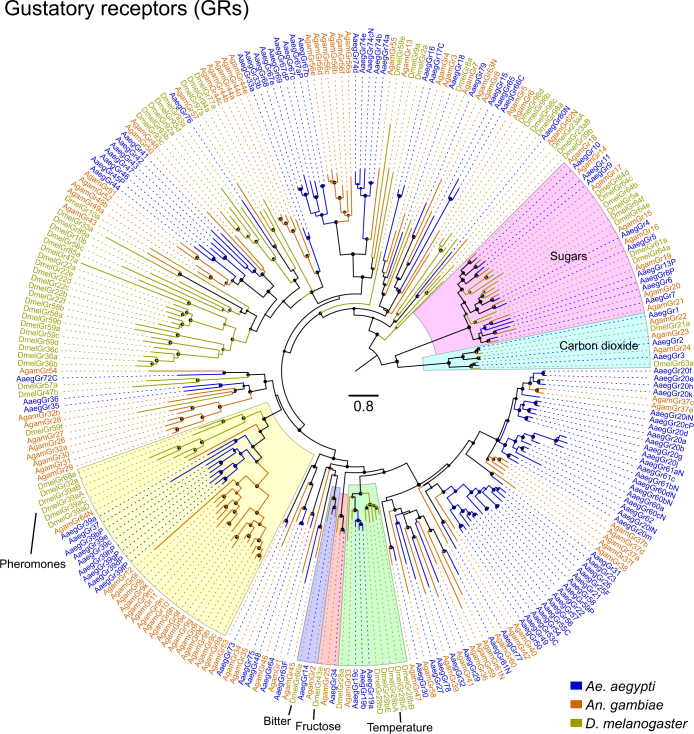
Phylogenetic tree of the gustatory receptor gene families from *Ae. aegypti*, *An. gambiae* and *D. melanogaster*. Maximum likelihood gustatory receptor tree was rooted with the highly conserved and distantly related carbon dioxide and sugar receptor subfamilies, which together form a basal clade within the arthropod gustatory receptor family^89^. Subfamilies and lineages closely related to *D. melanogaster* gustatory receptors of known function are highlighted. Support levels for nodes are indicated by the size of black circles—reflecting approximate likelihood ratio tests (aLRT values ranging from 0 to 1 from PhyML v.3.0 run with default parameters^90^). Suffixes after protein names are C, minor assembly correction; F, major assembly modification; N, new model; P, pseudogene. Scale bar, amino acid substitutions per site.

**Extended Data Fig. 6:**
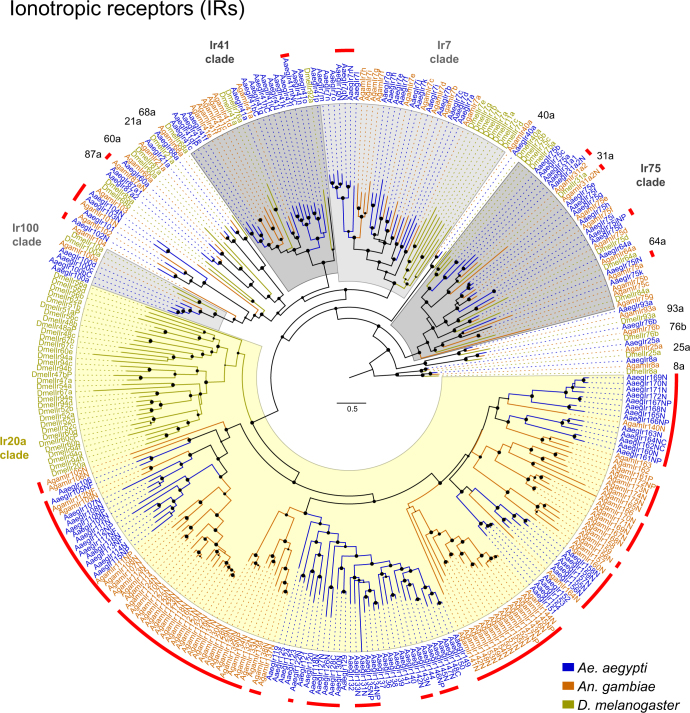
Phylogenetic tree of the ionotropic receptor gene families from *Ae. aegypti*, *An. gambiae* and *D. melanogaster*. Maximum likelihood phylogenetic tree of ionotropic receptor protein sequences from the indicated species rooted with highly conserved Ir8a and Ir25a proteins. Conserved proteins with orthologues in all species are named outside the circle, and previously unannotated ionotropic receptors are highlighted with red lines. Support levels for nodes are indicated by the size of black circles—reflecting approximate likelihood ratio tests (aLRT values ranging from - to 1 from PhyML v.3.0 run with default parameters^90^). Suffixes after protein names are C, minor assembly correction; F, major assembly modification; N, new model; P, pseudogene. Scale bar, amino acid substitutions per site.

**Extended Data Fig. 7:**
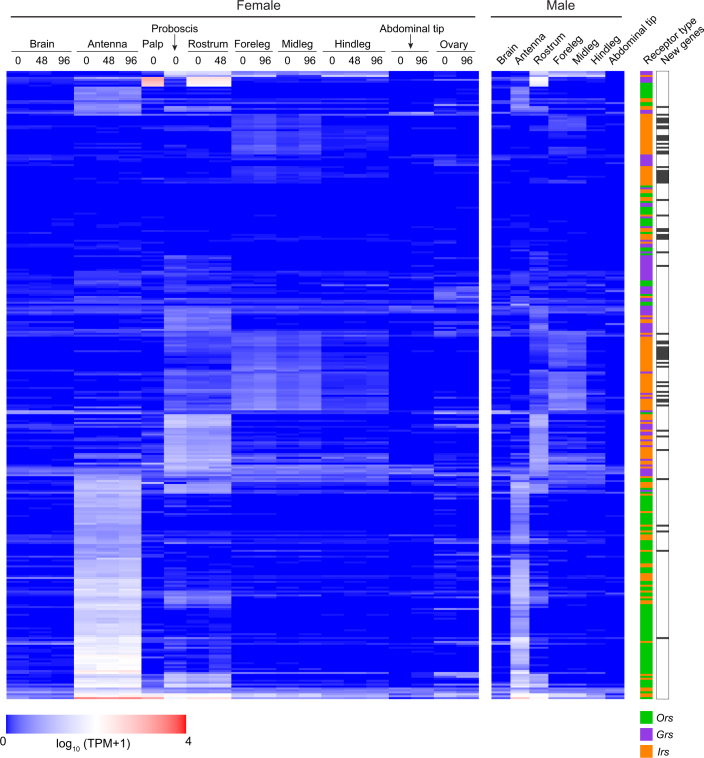
Chemosensory receptor expression in adult *Ae. aegypti* tissues. Previously published RNA-seq data^13^ were reanalysed using the new chemoreceptor annotations and genome assembly. Chemoreceptors have been clustered according to Euclidian distance of their expression vectors using the R function hclust. Expression is given for females at three stages of the gonotrophic cycle (0, 48 or 96 h after taking a blood-meal, for which 0 h indicates not blood-fed, 48 h indicates 48 h after the blood-meal, and 96 h indicates gravid). New genes are indicated by black bars on the right.

Large multi-gene families are very difficult to assemble and correctly annotate, because recently duplicated genes typically share high sequence similarities or can be misclassified as alleles of a single gene. We curated genes in large multi-gene families that encode proteases, G protein-coupled receptors, and chemosensory receptors using the improved AaegL5 genome and AaegL5.0 annotation. Serine proteases mediate immune responses^11^ and metalloproteases have been linked to vector competence and mosquito–*Plasmodium* interactions^12^. Gene models for over 50% of the 404 annotated serine proteases and metalloproteases in AaegL3.4 were improved in AaegL5.0, and we found 49 previously unannotated protease genes (Supplementary Data 13). G protein-coupled receptors are membrane proteins that respond to diverse external and internal sensory stimuli. We provide major corrections to gene models that encode 10 visual opsins and 17 dopamine and serotonin receptors (Extended Data Fig. 2f and Supplementary Data 14–16). Three large multi-gene families of insect chemosensory receptors are ligand-gated ion channels: odorant receptors (*OR* gene family), gustatory receptors (*GR* gene family) and ionotropic receptors (*IR* gene family). These collectively allow insects to sense a vast array of chemical cues, including carbon dioxide and human body odours that activate and attract female mosquitoes. We identified 117 odorant receptors, 72 gustatory receptors (encoding 107 transcripts) and 135 ionotropic receptors in the AaegL5 assembly (Fig. 2a, b, Extended Data Fig. 3 and Supplementary Data 17–20), inferred new phylogenetic trees for each family to investigate the relationship of these receptors in *Ae. aegypti*, *Anopheles gambiae* malaria mosquitoes and *D. melanogaster* (Extended Data Figs. 4–6), and revised expression estimates for adult male and female neural tissues using deep RNA-seq^13^ (Extended Data Fig. 7). Our annotation identified 54 new ionotropic receptor genes (Fig. 2b, Extended Data Fig. 3 and Supplementary Data 17), nearly doubling the known members of this family in *Ae. aegypti*. We additionally reannotated ionotropic receptors in *An. gambiae* and found 64 new genes. In *Ae. aegypti*, chemoreceptors are extensively clustered in tandem arrays (Fig. 2a and Extended Data Fig. 3), in particular on chromosome 3p, in which over a third of all chemoreceptor genes (*n* = 111) are found within a 109-Mb stretch. Although 71 gustatory receptor genes are scattered across chromosomes 2 and 3, only *AaegGr2*, a subunit of the carbon-dioxide receptor, is found on chromosome 1. Characterization of the full chemosensory receptor repertoire will enable the development of novel strategies to disrupt mosquito biting behaviour.

## Structure of the sex-determining M locus

Sex determination in *Aedes* and *Culex* mosquitoes is governed by a dominant male-determining factor (M factor) at a male-determining locus (M locus) on chromosome 1^14–16^. This chromosome is homomorphic between the sexes except for the M/m karyotype, meaning that males are M/m and females are m/m. Despite the recent discovery of the M factor *Nix* in *Ae. aegypti*^17^, which was entirely missing from the previous *Ae. aegypti* genome assemblies^2,4^, the full molecular properties of the M locus remain unknown. We aligned AaegL5 (from M/m males) and AaegL4 (from m/m females), and identified a region that contained *Nix* in AaegL5 at which the assemblies diverged and that may represent the divergent M/m locus (Fig. 3a). A de novo optical map assembly spanned the putative AaegL5 M locus and extended beyond its two borders. We estimated the size of the M locus at approximately 1.5 Mb, including an approximately 163-kb gap between contigs (Fig. 3a, c). We tentatively identified the female m locus as the region in AaegL4 not shared with the M locus-containing chromosome 1, but note that the complete phased structure of the divergent male M locus and corresponding female m locus remain to be determined. *Nix* contains a single intron of 100 kb, while *myo-sex*, a gene encoding a myosin heavy chain protein that has previously been shown to be tightly linked to the M locus^18^, is approximately 300 kb in length. More than 73.7% of the M locus is repetitive: long terminal repeat retrotransposons comprise 29.9% of the M locus compared to 11.7% genome-wide. Chromosomal FISH with *Nix*- and *myo-sex*-containing BAC clones^19^ showed that these genes co-localize to the 1p pericentromeric region (1p11) in only one homologous copy of chromosome 1, supporting the placement of the M locus at this position in AaegL5 (Fig. 3b). We investigated the differentiation between the sex chromosomes (Fig. 3d) using a chromosome quotient method to quantify regions of the genome with a strictly male-specific signal^20^. A sex-differentiated region in the LVP_AGWG strain extends to an approximately 100-Mb region surrounding the approximately 1.5-Mb M locus. This is consistent with the recent analysis of male–female *F*_ST_ in wild population samples and linkage map intercrosses^21^ and could be explained by a large region of reduced recombination encompassing the centromere and M locus^22^. The availability of a more completely assembled mosquito M locus provides opportunities to study the evolution and maintenance of homomorphic sex-determining chromosomes. The sex-determining chromosome of *Ae. aegypti* may have remained homomorphic at least since the evolutionary divergence between the *Aedes* and *Culex* genera more than 50 million years ago. With the more completely assembled M locus, we can investigate how these chromosomes have avoided the proposed eventual progression into heteromorphic sex chromosomes^23^.

## Structural variation and gene families

**Extended Data Fig. 8:**
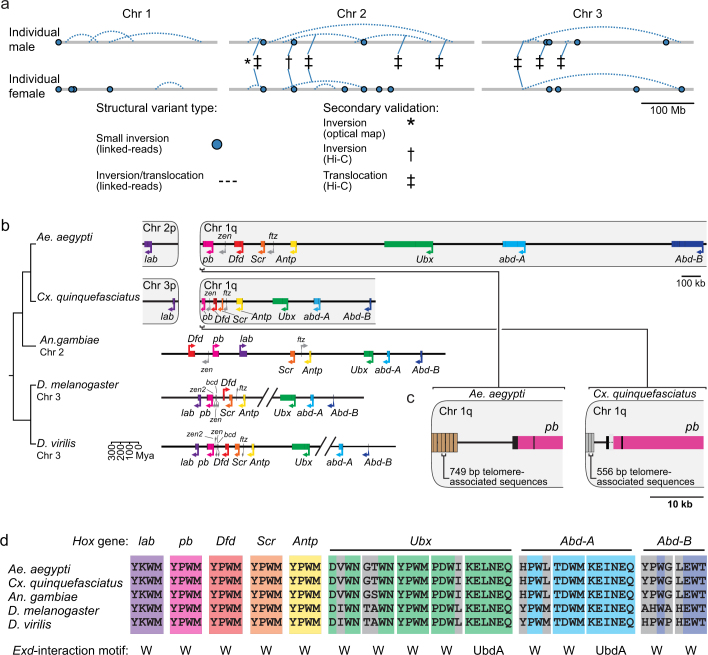
Structural variation, the *Hox* gene cluster and *Hox* cofactor motifs. **a**, Linked-read sequencing of two individuals from the LVP_AGWG strain identified putative structural variants in the AaegL5 assembly. **b**, Comparative genomic arrangement of the Hox cluster (*HOXC*) in five species (Supplementary Data 22). Note the split of *labial* (*lab*) and *proboscipedia* (*pb*) between two chromosomes in *Ae. aegypti* and *Cx. quinquefasciatus*. Owing to chromosome arm exchange, chromosome 3p in *Cx. quinquefasciatus* is the homologue of chromosome 2p in *Ae. aegypti*^4^. **c**, Repeats in putative telomere-associated sequences downstream of *pb* in both species. **d**, Motifs known to mediate protein–protein interactions with the Hox cofactor Extradenticle (*Exd*)^91^ from the five indicated species are aligned using Clustal-Omega. Perfectly aligned residues are coloured according to *Hox* gene identity, non-conserved residues are grey.

Structural variation is associated with the capacity to vector pathogens^24^. We produced ‘read cloud’ Illumina sequencing libraries of linked reads with long-range (around 80 kb) phasing information from one male and one female mosquito using the 10X Genomics Chromium platform to investigate structural variants, including insertions, deletions, translocations and inversions, in individual mosquitoes. We observed abundant small-scale insertions and deletions (indels; 26 insertions and 81 deletions called, median 42.9 kb) and inversions and/or translocations (29 called) in these two individuals (Extended Data Fig. 8a and Supplementary Data 21). Eight of the inversions and translocations coincided with structural variants seen independently by Hi-C or FISH, suggesting that those variants are relatively common within this population and can be detected by different methods. AaegL5 will provide a foundation for the study of structural variants across *Ae. aegypti* populations.

*Hox* genes encode highly conserved transcription factors that specify segment identity along the anterior–posterior body axis of all metazoans^25^. In most vertebrates, *Hox* genes are clustered in a co-linear arrangement, although they are often disorganized or split in other animal lineages^26^. All expected *Hox* genes are present as a single copy in *Ae. aegypti*, but we identified a split between *labial* and *proboscipedia* placing *labial* on a separate chromosome (Extended Data Fig. 8b and Supplementary Data 22). We confirmed this in AaegL4, which was generated with Hi-C contact maps from a different *Ae. aegypti* strain^4^, and note a similar arrangement in *Culex quinquefasciatus*, suggesting that it occurred before these two species diverged. Although this is not unprecedented^27^, a unique feature of this organization is that both *labial* and *proboscipedia* appear to be close to telomeres.

Glutathione *S*-transferases (GSTs) are a large multi-gene family involved in the detoxification of compounds such as insecticides. Increased GST activity has been associated with resistance to multiple classes of insecticides, including organophosphates, pyrethroids and the organochlorine dichlorodiphenyltrichloroethane (DDT)^28^. Amplification of detoxification genes is one mechanism by which insects can develop insecticide resistance^29^. We found that three insect-specific GST epsilon (GSTe) genes on chromosome 2, located centrally in the cluster (*GSTe2*, *GSTe5 *and *GSTe7*), are duplicated four times in AaegL5 relative to AaegL3 (Fig. 4a, b and Supplementary Data 23). Short Illumina read coverage and optical maps confirmed the copy number and arrangement of these duplications in AaegL5 (Fig. 4c, d), and analysis of whole-genome sequencing data for four additional laboratory colonies showed variable copy numbers across this gene cluster (Fig. 4d). GSTe2 is a highly efficient metaboliser of DDT^30^, and it is noteworthy that the cDNA from three GST genes in the quadruplication was detected at higher levels in DDT-resistant *Ae. aegypti* mosquitoes from southeast Asia^31^.

## Genome-wide genetic variation

**Extended Data Fig. 9:**
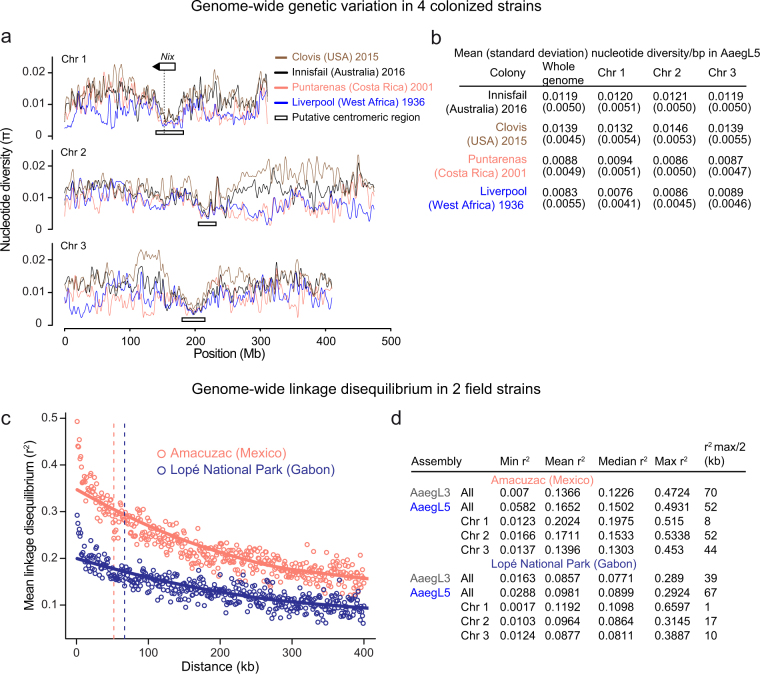
Population genomic structure and linkage disequilibrium analysis of *Ae. aegypti* strains. **a**, Chromosomal patterns of nucleotide diversity (*π*) in four strains of *Ae. aegypti* measured in 100-kb non-overlapping windows and presented as a LOESS-smoothed curve. **b**, Mean nucleotide diversity in the strains in **a**, with s.d. indicated in parentheses. Nucleotide diversity (*π*) was measured in non-overlapping 100-kb windows. The Liverpool and Costa Rica colonies maintain extensive diversity despite being colonized in the laboratory more than a decade ago, but show reduced genome-wide diversity (on the order of 30–40%) relative to the more recently laboratory colonized Innisfail and Clovis. **c**, Pairwise linkage disequilibrium between SNPs located within the same chromosome estimated from 28 wild-caught individuals from the indicated populations. Each point represents the mean linkage disequilibrium for that set of binned SNP pairs. Solid lines are LOESS-smoothed curves, and dashed lines correspond to *r*^2^_max_/2. Inclusion of additional individuals available from the Amacuzac population (up to 137) had a minimal effect on the linkage disequilibrium estimations (∆*R*^2^ < 0.017; data not shown). **d**, Linkage disequilibrium (*r*^*2*^) values along the *Ae. aegypti* AaegL5 genome assembly based on pairwise SNP comparisons. Data were obtained from the average *r*^*2*^ of SNPs in 1-kb bins.

Measurement of genetic variation within and between populations is important for inferring ongoing and historic evolution in a species^32^. To understand genomic diversity in *Ae. aegypti*, which spread in the last century from Africa to tropical and subtropical regions around the world, we performed whole-genome resequencing on four laboratory colonies. Chromosomal patterns of nucleotide diversity should correlate with regional differences in meiotic recombination rates^33^. We observed pronounced declines in genetic diversity near the centre of each chromosome (Extended Data Fig. 9a, b), providing independent corroboration of the estimated position of each centromere by Hi-C (Extended Data Fig. 2a).

To investigate linkage disequilibrium in geographically diverse populations of *Ae. aegypti*, we first mapped Affymetrix SNP-Chip markers that were designed using AaegL3^34^ to positions on AaegL5. We genotyped 28 individuals from two populations from Amacuzac, Mexico and Lopé National Park, Gabon and calculated the pairwise linkage disequilibrium of single-nucleotide polymorphisms (SNPs) from 1-kb bins both genome-wide and within each chromosome (Extended Data Fig. 9c, d). The maximum linkage disequilibrium in the Mexican population is approximately twice that of the population from Gabon, which probably reflects a recent bottleneck associated with the spread of this species out of Africa.

## Dengue competence and pyrethroid resistance

**Extended Data Fig. 10:**
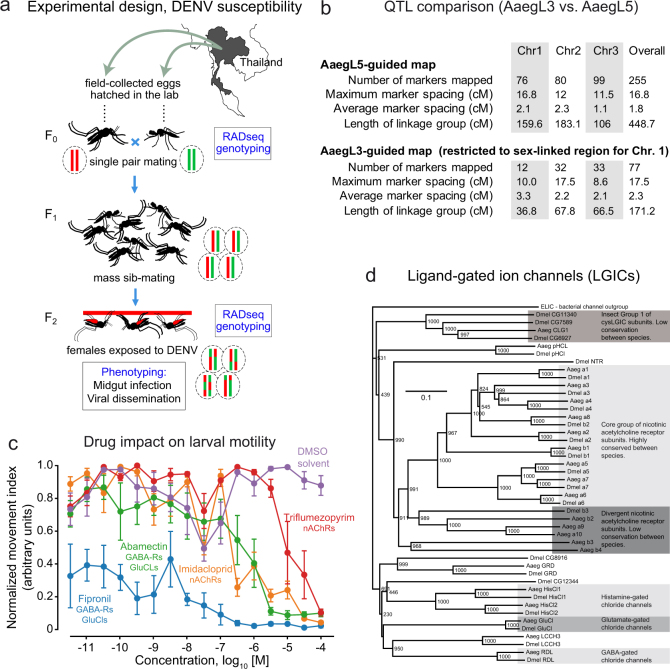
QTL analysis of DENV competence in *Ae. aegypti* and Cys-loop LGICs. **a**, Schematic representation of the experimental workflow for testing DENV competence in *Ae. aegypti*, related to Fig. 5b–d. **b**, Comparison of QTL map density constructed against AaegL3 or AaegL5 assemblies. **c**, Concentration–response curves showing the effect on *Ae. aegypti* larval motility of insecticides currently used in veterinary and agricultural applications (mean ± s.e.m., *n* = 7). **d**, Phylogenetic tree of Cys-loop LGIC subunits for *Ae. aegypti* and *D. melanogaster*. The accession numbers of the *D. melanogaster* sequences used in constructing the tree are: Dα1 (CAA30172), Dα2 (CAA36517), Dα3 (CAA75688), Dα4(CAB77445), Dα5 (AAM13390), Dα6 (AAM13392), Dα7(AAK67257), Dβ1 (CAA27641), Dβ2 (CAA39211), Dβ3 (CAC48166), GluCl (AAG40735), GRD (Q24352), HisCl1 (AAL74413), HisCl2 (AAL74414), LCCH3 (AAB27090), Ntr (NP_651958), pHCl (NP_001034025), RDL (AAA28556). For *Ae. aegypti* sequences, see Supplementary Data 24. ELIC (Erwinia ligand-gated ion channel), which is an ancestral Cys-loop LGIC found in bacteria (accession number P0C7B7), was used as an outgroup. Scale bar, amino acid substitutions per site.

To illustrate the value of AaegL5 for mapping quantitative trait loci (QTLs), we used restriction site-associated DNA (RAD) markers to locate QTLs underlying dengue virus (DENV) vector competence. We identified and genotyped RAD markers in the F_2_ progeny of a laboratory cross between wild *Ae. aegypti* founders from Thailand^35^ (Extended Data Fig. 10a). For this population, 197 F_2_ females had previously been scored for DENV vector competence against four different DENV isolates (two isolates from serotype 1 and two from serotype 3)^35^. The newly developed linkage map included a total of 255 RAD markers (Fig. 5a) with perfect concordance between genetic distances in centiMorgans (cM) and AaegL5 physical coordinates in Mb (Fig. 5a, c, d). We detected two significant QTLs on chromosome 2 that underlie the likelihood of DENV dissemination from the midgut (that is, systemic infection), an important component of DENV vector competence^36^. One QTL was associated with a generalist effect across DENV serotypes and isolates, whereas the other was associated with an isolate-specific effect (Fig. 5b, c). QTL mapping powered by AaegL5 will make it possible to understand the genetic basis of *Ae. aegypti* vector competence for arboviruses.

Pyrethroid insecticides are used to combat mosquitoes, including *Ae. aegypti*, and emerging resistance to these compounds is a global problem^37^. Understanding the mechanisms that underlie insecticide targets and resistance in different mosquito populations is critical to combating arboviral pathogens. Many insecticides act on ion channels, and we curated members of the Cys-loop ligand-gated ion channel (Cys-loop LGIC) superfamily in AaegL5. We found 22 subunit-encoding Cys-loop LGICs (Extended Data Fig. 10d and Supplementary Data 24), of which 14 encode nicotinic acetylcholine receptor (nAChR) subunits. nAChRs consist of a core group of subunit-encoding genes (α1–α8 and β1) that are highly conserved between insect species, and at least one divergent subunit^38^. Whereas *D. melanogaster* possesses only one divergent nAChR subunit, *Ae. aegypti* has five. We found that agricultural and veterinary insecticides impaired the motility of *Ae. aegypti* larvae (Extended Data Fig. 10c), suggesting that these Cys-loop LGIC-targeting compounds have potential as mosquito larvicides. The improved annotation presented here provides a valuable resource for investigating insecticide efficacy.

To demonstrate how a chromosome-scale genome assembly informs genetic mechanisms of insecticide resistance, we performed a genome-wide population genetic screen for SNPs correlating with resistance to deltamethrin in *Ae. aegypti* collected in Yucatán, Mexico, where pyrethroid-resistant and -susceptible populations co-exist (Fig. 5e). We uncovered an association with non-synonymous changes to three amino acid residues of the voltage-gated sodium channel VGSC, a known target of pyrethroids (Fig. 5f). The gene model for *VGSC*, a complex locus spanning nearly 500 kb in AaegL5, was incomplete and highly fragmented in AaegL3. SNPs in this region have a lower expected heterozygosity (*H*_exp_) in the resistant compared to the susceptible population, suggesting that they are part of a selective sweep for the resistance phenotype surrounding *VGSC* (Fig. 5g). Accurately associating SNPs with phenotypes requires a fully assembled genome, and we expect that AaegL5 will be critical to understanding the evolution of insecticide resistance and other important traits.

## Summary

The high-quality genome assembly and annotation described here will enable major advances in mosquito biology, and has already allowed us to carry out a number of experiments that were previously impossible. The highly contiguous AaegL5 genome permitted high-resolution genome-wide analysis of genetic variation and the mapping of loci for DENV vector competence and insecticide resistance. A new appreciation of copy number variation in insecticide-detoxifying *GSTe* genes and a more complete accounting of Cys-loop LGICs will catalyse the search for new resistance-breaking insecticides. A doubling in the known number of chemosensory ionotropic receptors provides opportunities to link odorants and tastants on human skin to mosquito attraction, a key first step in the development of novel mosquito repellents. ‘Sterile Insect Technique’ and ‘Incompatible Insect Technique’ show great promise to suppress mosquito populations^39^, but these population suppression methods require that only males are released. A strategy that connects a gene for male determination to a gene drive construct has been proposed to effectively bias the population towards males over multiple generations^40^, and improved understanding of M locus evolution and the function of its genetic content should facilitate genetic control of mosquitoes that infect many hundreds of millions of people with arboviruses every year^1^.

## Methods

### Data reporting

No statistical methods were used to predetermine sample size. The experiments were not randomized and the investigators were not blinded to allocation during experiments and outcome assessment.

### Ethics information

The participation of one human subject in blood-feeding mosquitoes was approved and monitored by The Rockefeller University Institutional Review Board (IRB protocol LVO-0652). This subject gave their written and informed consent to participate.

### Mosquito rearing and DNA preparation

*Ae. aegypti* eggs from a strain labelled ‘LVP_ib12’ were supplied by M.V.S. from a colony maintained at Virginia Tech. We performed a single pair cross between a male and female individual to generate material for Hi-C, Bionano optical mapping, flow cytometry, SNP-Chip analysis of strain variance, paired-end Illumina sequencing and 10X Genomics linked reads (Extended Data Fig. 1a). The same single male was crossed to a single female in two additional generations to generate high-molecular weight (HMW) genomic DNA for Pacific Biosciences long-read sequencing and to establish a colony (LVP_AGWG). Rearing was performed as previously described^13^ and all animals were offered a human arm as a blood source.

### SNP analysis of mosquito strains

Data were generated as described^34^, and PCA was performed using LEA 2.0 available for R v.3.4.0^41,42^. The following strains were used: *Ae. aegypti* LVP_AGWG (samples from the laboratory strain used for the AaegL5 genome assembly, reared as described in Extended Data Fig. 1a by a single pair mating in 2016 from a strain labelled LVP_ib12 maintained at Virginia Tech), *Ae. aegypti* LVP_ib12 (laboratory strain, LVP_ib12, provided in 2013 by D. Severson, University of Notre Dame), *Ae. aegypti* LVP_MR4 (laboratory strain labelled LVP_ib12 obtained in 2016 from MR4 at the Centers for Disease Control via BEI Resources catalogue MRA-735), *Ae. aegypti* Yaounde, Cameroon (field specimens collected in 2014 and provided by B. Kamgang), *Ae. aegypti* Rockefeller (laboratory strain provided in 2016 by G. Dimopoulos, Johns Hopkins Bloomberg School of Public Health), *Ae. aegypti* Key West, Florida (field specimens collected in 2016 and provided by W. Tabachnick). Strains used for the linkage disequilibrium data presented in Extended Data Fig. 9c, d were: *Ae. aegypti* from Amacuzac, Morelos, Mexico (field specimens collected in 2016 and provided by C. Gonzalez Acosta) and *Ae. aegypti* from La Lope National park forest, Gabon (field specimens collected and provided by S. Xia).

### Flow cytometry

Genome size was estimated by flow cytometry as described^43^, except that the propidium iodide was added at a concentration of 25 μl mg^−1^, not 50 μl mg^−1^, and samples were stained in the cold and dark for 24 h to allow the stain to fully saturate the sample. In brief, nuclei were isolated by placing a single frozen head of an adult sample along with a single frozen head of an adult *Drosophila virilis* female standard from a strain with 1C = 328 Mb into 1 ml of Galbraith buffer (4.26 g MgCl_2_, 8.84 g sodium citrate, 4.2 g 3-[*N*-morpholino] propane sulfonic acid (MOPS), 1 ml Triton X-100 and 1 mg boiled RNase A in 1 l of ddH_2_O, adjusted to pH 7.2 with HCl and filtered through a 0.22-μm filter)^44^ and grinding with 15 strokes of the A pestle at a rate of 3 strokes per 2 s. The resultant ground mixture was filtered through a 60-μm nylon filter (Spectrum Labs). Samples were stained with 25 μg of propidium iodide and held in the cold (4 °C) and dark for 24 h at which time the relative red fluorescence of the 2C nuclei of the standard and sample were determined using a Beckman Coulter CytoFlex flow cytometer with excitation at 488 nm. At least 2,000 nuclei were scored under each 2C peak and all scored peaks had a coefficient of variation of 2.5 or less^43,44^. Average channel numbers for sample and standard 2C peaks were scored using CytExpert software version 1.2.8.0 supplied with the CytoFlex flow cytometer. Significant differences among strains were determined using Proc GLM in SAS with both a Tukey and a Sheffé option. Significance levels were the same with either option. Genome size was determined as the ratio of the mean channel number of the 2C sample peak divided by the mean channel number of the 2C *D. virilis* standard peak times 328 Mb, where 328 Mb is the amount of DNA in a gamete of the standard. The following species/strains were used: *Ae. mascarensis* (collected by A. Bheecarry on Mauritius in December 2014. Colonized and maintained by J.R.P.), *Ae. aegypti* Ho Chi Minh City F13 (provided by D. J. Gubler, Duke-National University of Singapore as F_1_ eggs from females collected in Ho Chi Minh City in Vietnam, between August and September 2013. Colonized and maintained for 13 generations by A.G.-S.), *Ae. aegypti* Rockefeller (laboratory strain provided by D. Severson, Notre Dame), *Ae. aegypti* LVP_AGWG (reared as described in Extended Data Fig. 1a from a strain labelled LVP_ib12 maintained by M.V.S. at Virginia Tech), *Ae. aegypti* New Orleans F8 (collected by D. Wesson in New Orleans 2014, colonized and maintained by J.R.P. through 8 generations of single pair mating), *Ae. aegypti* Uganda 49-ib-G5 (derived by C.S.M. through 5 generations of full-sibling mating of the U49 colony established from eggs collected by J.-P. Mutebi in Entebbe, Uganda in March 2015).

### Pacific Biosciences library construction, sequencing and assembly

#### HMW DNA extraction for Pacific Biosciences sequencing

HMW DNA extraction for Pacific Biosciences sequencing was performed using the Qiagen MagAttract Kit (67563) following the manufacturer’s protocol with approximately 80 male sibling pupae in batches of 25 mg.

#### SMRTbell library construction and sequencing

Three libraries were constructed using the SMRTbell Template Prep Kit 1.0 (Pacific Biosciences). In brief, genomic DNA (gDNA) was mechanically sheared to 60 kb using the Megaruptor system (Diagenode) followed by DNA damage repair and DNA end repair. Universal blunt hairpin adapters were then ligated onto the gDNA molecules after which non-SMRTbell molecules were removed with exonuclease. Pulse-field gels were run to assess the quality of the SMRTbell libraries. Two libraries were size-selected using SageELF (Sage Science) at 30 kb and 20 kb, the third library was size-selected at 20 kb using BluePippin (Sage Science). Prior to sequencing, another DNA-damage repair step was performed and quality was assessed with pulse-field gel electrophoresis. A total of 177 SMRT cells were run on the RS II using P6-C4 chemistry and 6 h videos.

#### Contig assembly and polishing

A diploid contig assembly was carried out using FALCON v.0.4.0 followed by the FALCON-Unzip module (revision 74eefabdcc4849a8cef24d1a1bbb27d953247bd7)^5^. The resulting assembly contains primary contigs, a partially phased haploid representation of the genome and haplotigs, which represent phased alternative alleles for a subset of the genome. Two rounds of contig polishing were performed. For the first round, as part of the FALCON-Unzip pipeline, primary contigs and secondary haplotigs were polished using haplotype-phased reads and the Quiver consensus caller^45^. For the second round of polishing we used the ‘resequencing’ pipeline in SMRT Link v.3.1, with primary contigs and haplotigs concatenated into a single reference. Resequencing maps all raw reads to the combined assembly reference with BLASR (v.3.1.0)^46^, followed by consensus calling with Arrow (https://github.com/PacificBiosciences/GenomicConsensus)^46^.

### Hi-C sample preparation and analysis

#### Library preparation

In brief, insect tissue was crosslinked and homogenized. The nuclei were then extracted and permeabilized, and libraries were prepared using a modified version of the in situ Hi-C protocol that we optimized for insect tissue^47^. Separate libraries were prepared for samples derived from three individual male pupae. The resulting libraries were sequenced to yield 118 million, 249 million and 114 million reads (coverage: 120×) and these were processed using Juicer^48^.

#### Hi-C approach

Using the results of FALCON-Unzip as input, we used Hi-C to correct misjoins, to order and orient contigs, and to merge overlaps (Extended Data Fig. 1c–e). The Hi-C based assembly procedure that we used is described in detail in the Supplementary Methods and Supplementary Discussion. Notably, both primary contigs and haplotigs were used as input. This was essential, because Hi-C data identified genomic loci in which the corresponding sequence was absent in the primary FALCON-Unzip contigs, and present only in the haplotigs; the loci would have led to gaps, instead of contiguous sequence, if the haplotigs were excluded from the Hi-C assembly process (Extended Data Fig. 1e).

#### Hi-C scaffolding

We set aside 359 FALCON-Unzip contigs shorter than 20 kb, because such contigs are more difficult to accurately assemble using Hi-C. To generate chromosome-length scaffolds, we used the Hi-C maps and the remaining contigs as inputs to the previously described algorithms^4^. Note that both primary contigs and haplotigs were used as input. We performed quality control, manual polishing and validation of the scaffolding results using Assembly Tools^49^. This produced three chromosome-length scaffolds. Notably, the contig N50 decreased slightly, to 929,392 bp, because of the splitting of misjoined contigs.

#### Hi-C alternative haplotype merging

Examination of the initial chromosome-length scaffolds using Assembly Tools^49^ revealed that extensive undercollapsed heterozygosity was present. In fact, most genomic intervals were repeated, with variations, on two or more unmerged contigs. This suggested that the levels of undercollapsed heterozygosity were unusually high, and that the true genome length was far shorter than either the total length of the Pacific Biosciences contigs (2,047 Mb), or the initial chromosome-length scaffolds (1,973 Mb). Possible factors that could have contributed to the unusually high rate of undercollapsed heterozygosity seen in the FALCON-Unzip Pacific Biosciences contigs relative to prior contig sets for *Ae. aegypti* generated using Sanger sequencing (AaegL3)^2^, include high heterozygosity levels in the species and incomplete inbreeding in the samples that we sequenced. The merge algorithm described previously^4^ detects and merges draft contigs that overlap one another owing to undercollapsed heterozygosity. Because undercollapsed heterozygosity does not affect most loci in a typical draft assembly, the default parameters are relatively stringent. We adopted more permissive parameters for AaegL5 to accommodate the exceptionally high levels of undercollapsed heterozygosity, but found that the results would occasionally merge contigs that did not overlap. To avoid these false positives, we developed a procedure to manually identify and ‘whitelist’ regions of the genome containing no overlap, based on both Hi-C maps and LASTZ alignments (Extended Data Fig. 1c, Supplementary Methods and Supplementary Discussion). We then reran the merge step, using the whitelist as an additional input. Finally, we performed quality control of the results using Assembly Tools^49^, which confirmed the absence of the undercollapsed heterozygosity that we had previously observed. The resulting assembly contained three chromosome-length scaffolds (310 Mb, 473 Mb and 409 Mb), which spanned 94% of the merged sequence length. The assembly also contained 2,364 small scaffolds, which spanned the remaining 6% (Table 1). These lengths were consistent with the results of flow cytometry and the lengths obtained in prior assemblies. Notably, the merging of overlapping contigs using the above procedure frequently eliminated gaps, and thus greatly increased the contig N50, from 929,392 to 4,997,917 bp.

### Final gap-filling and polishing

#### Scaffolded assembly polishing

Following scaffolding and de-duplication, we performed a final round of arrow polishing. PBJelly^50^ from PBSuite version 15.8.24 was used for gapfilling of the de-duplicated HiC assembly (see ‘Protocol.xml’ in Supplementary Methods and Supplementary Discussion). After PBJelly, the liftover file was used to translate the renamed scaffolds to their original identifiers. For this final polishing step (run with SMRT Link v3.1 resequencing), the reference sequence included the scaffolded, gap-filled reference, as well as all contigs and contig fragments not included in the final scaffolds (https://github.com/skingan/AaegL5_FinalPolish). This reduces the likelihood that reads map to the wrong haplotype, by providing both haplotypes as targets for read mapping. For submission to NCBI, two scaffolds identified as mitochondrial in origin were removed (see below), and all remaining gaps on scaffolds were standardized to a length of 100 Ns to indicate a gap of unknown size. The assembly quality value was estimated using independent Illumina sequencing data from a single individual male pupa (library H2NJHADXY_1/2). Reads were aligned with BWA-MEM v.0.7.12-r1039^51^. FreeBayes v.1.1.0-50-g61527c5-dirty^52^ was used to call SNPs and short indels with the parameters -C 2 -0 -O -q 20 -z 0.10 -E 0 -X -u -p 2 -F 0.6. Any SNP and short indels showing heterozygosity (for example, 0/1 genotype) were excluded. The quality value was estimated at 34.75 using the PHRED formula with SNPs as the numerator (597,798) and number of bases with at least threefold coverage as the denominator, including alternate alleles (1,782,885,792).

#### Identification of mitochondrial contigs

During the submission process for this genome, two contigs were identified as mitochondrial in origin and were removed from the genomic assembly, manually circularized, and submitted separately. The mitochondrial genome is available as GenBank accession number MF194022.1, RefSeq accession number NC_035159.1.

### Bionano optical mapping

#### HMW DNA extraction

HMW DNA extraction was performed using the Bionano Animal Tissue DNA Isolation Kit (RE-013-10), with a few protocol modifications. A single-cell suspension was made as follows. First, 47 mg of frozen male pupae was fixed in 2% v/v formaldehyde in Homogenization Buffer from the kit (Bionano 20278), for 2 min on ice. Then, the pupae were roughly homogenized by blending for 2 s, using a rotor–stator tissue homogenizer (TissueRuptor, Qiagen 9001271). After another 2 min fixation, the tissue was finely homogenized by running the rotor–stator for 10 s. Subsequently, the homogenate was filtered with a 100-μm nylon filter, fixed with ethanol for 30 min on ice, spun down, and washed with more Homogenization Buffer (to remove residual formaldehyde). The final pellet was resuspended in Homogenization Buffer. A single agarose plug was made using the resuspended cells, using the CHEF Mammalian Genomic DNA Plug Kit (BioRad 170-3591), following the manufacturer’s instructions. The plug was incubated with Lysis Buffer (Bionano 20270) and Puregene Proteinase K (Qiagen 1588920) overnight at 50 °C, then again the following morning for 2 h (using new buffer and Proteinase K). The plug was washed, melted and solubilized with GELase (Epicentre G09200). The purified DNA was subjected to 4 h of drop dialysis (Millipore, VCWP04700) and quantified using the Quant-iT PicoGreen dsDNA Assay Kit (Invitrogen/Molecular Probes P11496).

#### DNA labelling

DNA was labelled according to commercial protocols using the DNA Labelling Kit NLRS (RE-012-10, Bionano Genomics). Specifically, 300 ng of purified genomic DNA was nicked with 7 U nicking endonuclease Nt.BspQI (New England BioLabs, NEB) at 37 °C for 2 h in NEBuffer3. The nicked DNA was labelled with a fluorescent-dUTP nucleotide analogue using Taq polymerase (NEB) for 1 h at 72 °C. After labelling, the nicks were ligated with Taq ligase (NEB) in the presence of dNTPs. The backbone of fluorescently labelled DNA was counterstained with YOYO-1 (Invitrogen).

#### Data collection

The DNA was loaded onto the nanochannel array of Bionano Genomics IrysChip by electrophoresis of DNA. Linearized DNA molecules were then imaged automatically followed by repeated cycles of DNA loading using the Bionano Genomics Irys system. The DNA-molecule backbones (YOYO-1 stained) and locations of fluorescent labels along each molecule were detected using the in-house-generated software package, IrysView. The set of label locations of each DNA molecule defines an individual single-molecule map. After filtering data using normal parameters (molecule reads with length greater than 150 kb, a minimum of 8 labels and standard filters for label and backbone signals), a total of 299 Gb and 259 Gb of data were collected from Nt.BspQI and Nb.BssSI samples, respectively.

#### De novo genome map assembly

De novo assembly was performed with non-haplotype aware settings (optArguments_nonhaplotype_noES_irys.xml) and pre-release version of Bionano Solve3.1 (Pipeline version 6703 and RefAligner version 6851). On the basis of the overlap–layout–Consensus paradigm, pairwise comparisons of all DNA molecules were performed to create an overlap graph, which was then used to create the initial consensus genome maps. By realigning molecules to the genome maps (RefineB *P* = 10 × 10^−11^) and by using only the best match molecules, a refinement step was performed to refine the label positions on the genome maps and to remove chimeric joins. Next, during an extension step, the software aligned molecules to genome maps (extension, *P* = 10 × 10^−11^), and extended the maps based on the molecules aligning past the map ends. Overlapping genome maps were then merged using a merge *P*-value cut-off of 10 *P* = 10 × 10^−15^. These extension and merge steps were repeated five times before a final refinement was applied to ‘finish’ all genome maps (refine final, *P* = 10 × 10^−11^). Two genome map de novo assemblies, one with nickase Nt.BspQI and the other with nickase Nb.BssSI, were constructed. Alignments between the constructed de novo genome assemblies and the L5 assembly were performed using a dynamic programming approach with a scoring function and a *P*-value cutoff of *P* = 10 × 10^−12^.

### Transposable element identification

#### Identification of known transposon elements

We first identified known transposable elements using RepeatMasker (version 3.2.6)^53^ against the mosquito TEfam (https://tefam.biochem.vt.edu/tefam/, data downloaded July 2017), a manually curated mosquito transposable-elements database. We then ran RepeatMasker using the TEfam database and Repbase transposable-elements library (version 10.05). RepeatMasker was set to default parameters with the -s (slow search) flag and NCBI/RMblast program (v.2.2.28).

#### De novo repeat family identification

We searched for repeat families and consensus sequences using the de novo repeat prediction tool RepeatModeler (version 1.0.8)^54^ using default parameters with RECON (version 1.07) and RepeatScout (1.0.5) as core programs. Consensus sequences were generated and classified for each repeat family. Then RepeatMasker was run on the genome sequences, using the RepeatModeler consensus sequence as the library.

#### Tandem repeats

We also predicted tandem repeats in the whole genome and in the repeatmasked genome using Tandem Repeat Finder^55^. Long tandem copies were identified using the ‘Match=2, Mismatch=7, Delta=7, PM=80, PI=10, Minscore=50 MaxPeriod=500’ parameters. Simple repeats, satellites and low complexity repeats were found using ‘Match=2, Mismatch=7, Delta=7, PM=80, PI=10, Minscore=50, and MaxPeriod=12’ parameters.

A file representing the coordinates of all identified repeat and transposable-element structures in AaegL5 can be found at https://github.com/VosshallLab/AGWG-AaegL5.

### Generation of RefSeq gene set annotation

The AaegL5 assembly was deposited at NCBI in June 2017 and annotated using the NCBI RefSeq Eukaryotic gene annotation pipeline^56^. Evidence to support the gene predictions came from over 9 billion Illumina RNA-seq reads, 67,000 Pacific Biosciences IsoSeq transcripts, 300,000 expressed sequence tags and well-supported proteins from *D. melanogaster* and other insects. Annotation Release 101 was made public in July 2017, and specific gene families were subjected to manual annotation and curation. Detailed descriptions of the manual annotation and curation of multigene families (*Hox* genes, proteases, opsins and biogenic amine receptors, chemosensory receptors and LGICs) can be found in the Supplementary Methods and Supplementary Discussion. See also https://www.ncbi.nlm.nih.gov/genome/annotation_euk/Aedes_aegypti/101/.

### Alignment of RNA-seq data to AaegL5 and quantification of gene expression

Published RNA-seq reads^13,57^ and unpublished RNA-seq reads from tissue-specific libraries produced by Verily Life Sciences were mapped to the RefSeq assembly GCF_002204515.2_AaegL5.0 with STAR aligner (v.2.5.3a)^58^ using the two-pass approach. Reads were first aligned in the absence of gene annotations using the following parameters: --outFilterType BySJout; --alignIntronMax 1000000; --alignMatesGapMax 1000000; --outFilterMismatchNmax 999; --outFilterMismatchNoverReadLmax 0.04; --clip3pNbases 1; --outSAMstrandField intronMotif; --outSAMattrIHstart 0; --outFilterMultimapNmax 20; --outSAMattributes NH HI AS NM MD; --outSAMattrRGline; --outSAMtype BAM SortedByCoordinate. Splice junctions identified during the first pass mapping of individual libraries were combined and supplied to STAR using the --sjdbFileChrStartEnd option for the second pass. Reads mapping to gene models defined by the NCBI annotation pipeline (GCF_002204515.2_AaegL5.0_genomic.gff) were quantified using featureCounts^59^ with default parameters. Count data were transformed to transcripts per million values using a custom Perl script. Details on libraries, alignment statistics and gene expression estimates (expressed in transcripts per million) are provided as Supplementary Data 4–8.

### Identification of ‘collapsed’ and ‘merged’ gene models from AaegL3.5 to AaegL5.0

VectorBase annotation AaegL3.5 was compared to NCBI *Ae. aegypti* annotation release 101 on AaegL5.0 using custom code developed at NCBI as part of NCBI’s eukaryotic genome annotation pipeline. First, assembly–assembly alignments were generated for AaegL3 (GCA_000004015.3) × AaegL5.0 (GCF_002204515.2) as part of NCBI’s Remap coordinate remapping service, as described at https://www.ncbi.nlm.nih.gov/genome/tools/remap/docs/alignments. The alignments are publicly available in NCBI’s Genome Data Viewer (https://www.ncbi.nlm.nih.gov/genome/gdv/), the Remap interface, and by FTP in either ASN.1 or GFF3 format (ftp://ftp.ncbi.nlm.nih.gov/pub/remap/Aedes_aegypti/2.1/). Alignments are categorized as either ‘first pass’ (reciprocity = 3) or ‘second pass’ (reciprocity = 1 or 2). First pass alignments are reciprocal best alignments, and are used to identify regions on the two assemblies that can be considered equivalent. Second pass alignments are cases in which two regions of one assembly have their best alignment to the same region on the other assembly. These are interpreted to represent regions in which two paralogous regions in AaegL3 have been collapsed into a single region in AaegL5, or vice versa.

For comparing the two annotations, both annotations were converted to ASN.1 format and compared using an internal NCBI program that identifies regions of overlap between gene, mRNA and coding sequence (CDS) features projected through the assembly–assembly alignments. The comparison was performed twice, first using only the first pass alignments, and again using only the second pass alignments corresponding to regions in which duplication in the AaegL3 assembly had been collapsed. Gene features were compared, requiring at least some overlapping CDS in both the old and new annotation to avoid noise from overlapping genes and comparisons between coding and non-coding genes. AaegL5.0 genes that matched to two or more VectorBase AaegL3.5 genes were identified. Matches were further classified as collapsed paralogues if one or more of the matches was through the second pass alignments, or as improvements due to increased contiguity or annotation refinement if the matches were through first pass alignments (for example, two AaegL3.5 genes represent the 5′ and 3′ ends of a single gene on AaegL5.0, such as *sex peptide receptor*. Detailed lists of merged genes are in Supplementary Data 10, 11.

### Comparison of alignment to AaegL3.4 and AaegL5.0

The sequences comprising transcripts from the AaegL5.0 gene set annotation were extracted from coordinates provided in GCF_002204515.2_AaegL5.0_genomic.gtf. Sequences corresponding to AaegL3.4 gene set annotations were downloaded from Vectorbase (https://www.vectorbase.org/download/aedes-aegypti-liverpooltranscriptsaaegl34fagz). Salmon (v.0.8.2)^60^ indices were generated with default parameters, and all libraries described in Supplementary Data 4 were mapped to both AaegL3.4 and AaegL5 sequences using ‘quant’ mode with default parameters. Mapping results are presented as Supplementary Data 9 and Fig. 1h.

### ATAC-seq

The previously described ATAC-seq protocol^61^ was adapted for *Ae. aegypti* brains. Individual brains from LVP_MR4 non-blood-fed females (Extended Data Fig. 2c, d) or females 48 h or 96 h after taking a human blood meal (data not shown) were dissected in 1× PBS, immediately placed in 100 μl ice-cold ATAC lysis buffer (10 mM Tris-Hcl, pH 7.4, 10 mM NaCl, 3 mM MgCl_2_, 0.1% IGEPAL CA-630), and homogenized in a 1.5-ml Eppendorf tube using 50 strokes of a Wheaton 1-ml PTFE-tapered tissue grinder. Animals at 96 h after the blood meal were deprived of access to a water oviposition site and were considered gravid at the time of dissection. Lysed brains were centrifuged at 400*g* for 20 min at 4 °C and the supernatant was discarded. Nuclei were resuspended in 52.5 μl 1× Tagmentation buffer (provided in the Illumina Nextera DNA Library Prep Kit) and 5 μl were removed to count nuclei on a haemocytometer. In total, 50,000 nuclei were used for each transposition reaction. The concentration of nuclei in Tagmentation buffer was adjusted to 50,000 nuclei in 47.5 μl Tagmentation buffer and 2.5 μl Tn5 enzyme was added (provided in the Illumina Nextera DNA Library Prep Kit). The remainder of the ATAC-seq protocol was performed as described^61^. The final library was purified and size-selected using double-sided AMPure XP beads (0.6×, 0.7×). The library was checked on an Agilent Bioanalyzer 2100 and quantified using the Qubit dsDNA HS Assay Kit. Resulting libraries were sequenced as 75-bp paired-end reads on an Illumina NextSeq500 platform at an average read depth of 30.5 million reads per sample. Raw fastq reads were checked for nucleotide distribution and read quality using FASTQC (http://www.bioinformatics.babraham.ac.uk/projects/fastqc) and mapped to the AaegL5 and AaegL3 versions of the *Ae. aegypti* genome using Bowtie v.2.2.9^62^. Aligned reads were processed using Samtools 1.3.1^63^ and Picard 2.6.0 (http://broadinstitute.github.io/picard/index.html) and only uniquely mapped and non-redundant reads were used for downstream analyses. To compare the annotation and assembly of the *sex peptide receptor* gene in AaegL3 and AaegL5, we used NCBI BLAST^64^ to identify AAEL007405 and AAEL010313 as gene fragments in AaegL3.4 annotation that map to *sex peptide receptor* in the AaegL5.0 genome (BLAST *E* values for both queries mapping to *sex peptide receptor* were 0.0). Next, we used GMAP^65^ to align AAEL007405 and AAEL010313 fasta sequences to AaegL5. The resulting GFF3 annotation file was used by Gviz^66^ to plot RNA-seq reads and sashimi plots as well as ATAC-seq reads in the region containing *sex peptide receptor*. Transcription start site analysis was performed using HOMER v.4.9^67^. In brief, databases containing 2-kb windows flanking transcription start sites genome-wide were generated using the ‘parseGTF.pl’ HOMER script from AaegL3.4 and AaegL5.0 GFF3 annotation files. Duplicate transcription start sites and transcription start sites that were within 20 bp from each other were merged using the ‘mergePeaks’ HOMER script. Coverage of ATAC-seq fragments in predicted transcription start site regions was calculated with the ‘annotatePeaks.pl’ script. Fold change in predicted transcription site regions was calculated by dividing the ATAC fragments per base pair per predicted transcription start site in the AaegL5.0 genome by ATAC fragments per base pair per predicted transcription start site in the AaegL3.4 genome at the 0 base pair point in each predicted transcription start site. Coverage histograms were plotted using ggplot v.2 2.2.1 in RStudio v.1.1.383, R v.3.4.2^42^.

### M locus analysis

#### Aligning chromosome assemblies and Bionano scaffolds

The boundaries of the M locus were identified by comparing the current AaegL5 assembly and the AaegL4 assembly^4^ using a program called LAST^68^ (data not shown). To overcome the challenges of repetitive hits, both AaegL5 and AaegL4 assemblies were twice repeat-masked^53^ against a combined repeat library of TEfam-annotated transposable elements (https://tefam.biochem.vt.edu/tefam/)^2^ and a RepeatModeler output^54^ from the *Anopheles* 16 Genomes project^69^. The masked sequences were then compared using BLASTn^64^ and we then set a filter for downstream analysis to include only alignment with ≥98% identity over 1,000 bp. After the identification of the approximate boundaries of the M locus (and m locus), which contains two male-specific genes, *myo-sex*^18^ and *Nix*^17^, we zoomed in by performing the same analysis on regions of the M locus and m locus plus 2 Mb flanking regions without repeatmasking. In this and subsequent analyses, only alignment with ≥98% identity over 500 bp were included. Consequently, approximate coordinates of the M locus and m locus were obtained on chromosome 1 of the AaegL5 and AaegL4 assemblies, respectively. Super-scaffold_63 in the Bionano optical map assembly was identified by BLASTN^64^ that spans the entire M locus and extends beyond its two borders.

#### Chromosome quotient analysis

The chromosome quotient (CQ)^20^ was calculated for each 1,000-bp window across all AaegL5 chromosomes. To calculate the CQ, Illumina reads were generated from two paired sibling female and male sequencing libraries. To generate libraries for CQ analysis, we performed two separate crosses of a single LVP_AGWG male to 10 virgin females. Eggs from this cross were hatched, and virgin male and female adults collected within 12 h of eclosion to verify their non-mated status. We generated genomic DNA from five males and five females from each of these crosses. Sheared genomic DNA was used to generate libraries for Illumina sequencing with the Illumina TruSeq Nano kit and sequencing performed on one lane of 150-bp paired-end sequencing on an Illumina NextSeq 500 in high-output mode.

For a given sequence *S*_*i*_ of a 1,000-bp window, ${{\rm{CQ}}}_{{S}_{i}}={F}_{{S}_{i}}/{M}_{{S}_{i}}$, where ${F}_{{S}_{i}}$ is the number of female Illumina reads aligned to *S*_*i*_, and ${M}_{{S}_{i}}$ is the number of male Illumina reads aligned to *S*_*i*_. Normalization was not necessary for these datasets because the mean and median CQs of the autosomes (chromosomes 2 and 3) are all near 1. A CQ value lower than the 0.05 indicates that the sequences within the corresponding 1,000-bp window had at least 20-fold more hits to the male Illumina data than to the female Illumina data. Not every 1,000-bp window produces a CQ value because many were completely masked by RepeatMasker^53^. To ensure that each CQ value represents a meaningful data point obtained with sufficient alignments, only sequences with more than 20 male hits were included in the calculation. The CQ values were then plotted against the chromosome location of the 1,000-bp window (Fig. 3d). Under these conditions, there is not a single 1,000-bp fragment on chromosomes 2 and 3 that showed CQ = 0.05 or lower.

#### Chromosome FISH

Slides of mitotic chromosomes were prepared from imaginal discs of fourth instar larvae following published protocols^3,70,71^. BAC clones were obtained from the University of Liverpool^19^ or from a previously described BAC library^72^. BACs were plated on agar plates (Thermo Fisher) and a single bacterial colony was used to grow an overnight bacterial culture in LB broth plates (Thermo Fisher) at 37 °C. DNA from the BACs was extracted using Sigma PhasePrep TM BAC DNA Kit (Sigma-Aldrich, NA-0100). BAC DNA for hybridization was labelled by nick translation with Cy3-, Cy5-dUTP (Enzo Life Sciences) or Fluorescein 12-dUTP (Thermo Fisher). Chromosomes were counterstained with DAPI in Prolong Gold Antifade (Thermo Fisher). Slides were analysed using a Zeiss LSM 880 Laser Scanning Microscope at 1,000× magnification. We note that localization of the M-locus to 1p11 is supported by both FISH and genomic analyses, but is contrary to a previously published placement at 1q21^17^.

### Identification and analysis of *Ae. aegypti* GST and P450 genes and validation of the repeat structure of the GSTe cluster

Genes were initially extracted from the AaegL5.0 genome annotation (NCBI release 101) by text search and filtered to remove ‘off target’ matches (for example, ‘cytochrome P450 reductase’), then predicted protein sequences of a small number of representative transcripts were used to search the protein set using BLASTp, to identify by sequence similarity sequences not captured by the text search (resulting in two additional P450s, no GSTs). For each gene family, predicted protein sequences were used to search the proteins of the AaegL3.4 gene set using BLASTp. All best matches, and additional matches with amino acid identity >90% were tabulated for each gene family (Supplementary Data 23) to identify both closely related paralogues and alleles annotated as paralogues in AaegL3.4. On the basis of a BLASTp search against the AaegL3.4 protein set, the two putative P450 genes not annotated as such in AaegL5.0 (encoding proteins XP_001649103.2 and XP_021694388.1) appear to be incorrect gene models in the AaegL5.0 annotation, which should in fact be two adjacent genes (*CYP9J20* and *CYP9J21* for XP_001649103.2; *CYP6P12* and *CYP6BZ1* for XP_021694388.1). Compared to AaegL3.4, which predicts a single copy each of *GSTe2*, *GSTe5* and *GSTe7*, the NCBI annotation of AaegL5.0 predicts three copies each of *GSTe2* and *GSTe5*, and four copies of *GSTe7*, arranged in a repeat structure. BLASTn searches revealed one additional copy each of *GSTe2* and *GSTe5 *in the third duplicated unit. Both contain premature termination codons owing to frameshifts, but these could be owing to uncorrected errors in the assembly. Error correction of all duplicated units was not possible owing to the inability to unequivocally align reads to units not ‘anchored’ to adjacent single-copy sequence.

To validate these tandem duplications, two lanes of Illumina whole-genome sequence data from a single pupa of the LVP_AGWG strain (H2NJHADXY) were aligned to a hard-masked version of the AaegL3 reference genome using Bowtie2 v.2.2.4^73^, with ‘--very-fast-local’ alignment parameters, an expected fragment size between 0 and 1,500 bp and relative orientation ‘forward–reverse’ (‘-I 0 -X 1500 –fr’). Aligned reads with a mapping quality less than 10 were removed using Samtools^63^. ‘featureCounts’, part of the ‘Subread’ v.1.5.0-p2 package^74^, was used to assign read pairs or reads (‘tags’) aligned to either DNA strand (‘-s 0’) and overlapping the coding regions of a gene by at least 100 bp (‘-t CDS–minOverlap 100’) to genes as an estimate of representation in the genome. Gene-wise tag counts were normalized by calculating the fragments per kilobase of gene length per million mapped reads (FPKM), using the following equation: (tag count/gene length in kb)/(sum of tag counts for all genes in genome/1,000,000).

Median FPKM for all genes in the genome was calculated (48.22), allowing FPKM of GSTe genes to be expressed relative to this. To examine strain differences in coverage at this cluster, we repeated this analysis for the four laboratory colonies analysed in Extended Data Fig. 9a, b. Median FPKM values across all genes ranged from 47.68 to 48.46 and gene-wise FPKM values normalized relative to these medians are plotted in Fig. 4d.

To visualize the sequence identity of the repeat structure in the GSTe cluster (Fig. 4b), we extracted the region spanning the cluster from AaegL5 chromosome 2 (351,597,324–351,719,186 bp) and performed alignment of Pacific Biosciences reads using minimap2 v2.1.1 (minimap2 -DP -k7 -w1 -B2 -r200 -g100 -m1 L5_gst.fa L5_gst.fa)^75^ and visualized these alignments using D-GENIES v1.2.0^92^ with minimum identity set to 0.15 and ‘Strong Precision’ enabled. To validate this repeat structure, we aligned two de novo optical maps created by Bionano using linearized DNA labelled with Nt.BspQI or Nb.BssSI. Single molecules from both maps span the entire region and the predicted restriction pattern provides support for the repeat structure as presented in AaegL5 (Fig. 4c).

### QTL mapping of DENV vector competence

In theory, a good-quality genome assembly is not necessary for QTL mapping procedures, because it relies on a linkage map that can be generated de novo from empirical recombination fractions. This typically involves three steps: (i) marker selection based on the Mendelian segregation ratios, (ii) marker assignment to linkage groups and (iii) marker ordering within each linkage group. However, if a high-quality reference genome assembly is available, the physical position of each marker can be determined and this prior information greatly facilitates steps (ii) and (iii), as shown below.

To demonstrate the improvement enabled by our new genome, we generated two linkage maps using the same Illumina sequence data that were aligned either to AaegL3 or AaegL5 genome assemblies. Although the initial number of markers was 616 in both cases, the final linkage map was 3.3-fold denser with AaegL5 than with AaegL3, as shown in Extended Data Fig. 10b. The difference in marker density between the two linkage maps is because many markers were filtered out from the AaegL3 data. Because the AaegL3 assembly is highly fragmented (>4,700 scaffolds), the position of each marker within the linkage groups is primarily determined from the recombination fractions. This ordering step is performed by creating a backbone with a subset of informative markers using a two-point algorithm, followed by the positioning of the remaining markers one at a time using a multi-point method. Only markers that are unambiguously positioned are kept in the final linkage map for QTL mapping. We note that AaegL4, which de-duplicated and scaffolded AaegL3 onto chromosomes^4^, would probably yield a similar improvement in mapping resolution.

Another complication arises for the chromosome 1 in *Ae. aegypti*, because recombination is strongly reduced in the region containing the sex-determining M locus. This leads to the severely biased segregation ratios for markers anchored to this linkage group. In our F_2_ intercross design, the fully sex-linked markers lacked the F_0_ paternal genotype in F_2_ females and segregated in the same manner as a backcross design. No linkage analysis method is readily available to deal with a chromosome that behaves like a mixture of intercross and backcross designs. Therefore, AaegL3-guided linkage analysis and QTL mapping for chromosome 1 were restricted to the fully sex-linked region based on a backcross design. By contrast, AaegL5-guided linkage analysis and QTL mapping for chromosome 1 made use of all markers regardless of their segregation ratios, allowing chromosome-wide coverage. As mentioned in the present manuscript, the only caveat is that our analytical procedure assumes autosomal Mendelian proportions, which may have resulted in over- or underestimation of linkage distances between markers on chromosome 1. The linkage map was iteratively refined by checking for misplaced markers based on visual inspection of the LOD/rf matrix.

Ultimately, AaegL5 has a markedly improved QTL mapping resolution over AaegL3. For instance, we mapped the same QTL underlying systemic DENV dissemination at the extremity of chromosome 2 with both AaegL3 and AaegL5. The 1.5 LOD support interval was much larger for the AaegL3-guided linkage map (0–50 cM, 74% of the linkage group) than for the AaegL5-guided linkage map (0–17 cM, 9% of the linkage group). We present this analysis in Extended Data Fig. 10b.

#### Mosquito crosses

A large F_2_ intercross was created from a single mating pair of field-collected F_0_ founders. Wild mosquito eggs were collected in Kamphaeng Phet Province, Thailand in February 2011 as previously described^35^. In brief, F_0_ eggs were allowed to hatch in filtered tap water and the larvae were reared until the pupae emerged in individual vials. *Ae. aegypti* adults were identified by visual inspection and maintained in an insectary under controlled conditions (28 ± 1 °C, 75 ± 5% relative humidity and 12:12-h light:dark cycle) with access to 10% sucrose. The F_0_ male and female initiating the cross were chosen from different collection sites to avoid creating a parental pair with siblings from the same wild mother^76,77^. Their F_1_ offspring were allowed to mass-mate and collectively oviposit to produce the F_2_ progeny (Extended Data Fig. 10a). A total of 197 females of the F_2_ progeny were used as a mapping population to generate a linkage map and detect QTLs underlying vector competence for DENV.

#### Vector competence

Four low-passage DENV isolates were used to orally challenge the F_2_ females as previously described^35^. In brief, four random groups of females from the F_2_ progeny were experimentally exposed to two virus isolates belonging to DENV serotype 1 (KDH0026A and KDH0030A) and two virus isolates belonging to DENV serotype 3 (KDH0010A and KDH0014A), respectively. All four virus isolates were derived from human serum specimens collected in 2010 from clinically ill patients who were infected with DENV at the Kamphaeng Phet Provincial Hospital^35^. Because the viruses were isolated in the laboratory cell culture, informed consent of the patients was not necessary for the present study. Complete viral genome sequences were previously deposited into GenBank (accession numbers HG316481, HG316582, HG316483, and HG316484)^35^. Phylogenetic analysis assigned the viruses to known viral lineages that were circulating in southeast Asia in the previous years^35^. Each isolate was amplified twice in C6/36 (*Aedes albopictus*) cell lines (maintained at AFRIMS in Bangkok, Thailand; used only to grow virus, not explicitly authenticated or checked for mycoplasma contamination) before vector competence assays. Four- to seven-day-old F_2_ females were starved for 24 h and offered an infectious blood meal for 30 min. Viral titres in the blood meals ranged from 2.0 × 10^4^ to 2.5 × 10^5^ plaque-forming units per ml across all isolates. Fully engorged females were incubated under the conditions described above. Vector competence was scored 14 days after the infectious blood meal according to two conventional phenotypes: (i) midgut infection and (ii) viral dissemination from the midgut. These binary phenotypes were scored based on the presence or absence of infectious particles in body and head homogenates, respectively. Infectious viruses were detected by plaque assay performed in LLC-MK2 (rhesus monkey kidney epithelial) cells as previously described^35,78^.

#### Genotyping

Mosquito gDNA was extracted using the NucleoSpin 96 Tissue Core Kit (Macherey-Nagel). For the F_0_ male, it was necessary to perform whole-genome amplification using the Repli-g Mini kit (Qiagen) to obtain a sufficient amount of DNA. F_0_ parents and females of the F_2_ progeny were genotyped using a modified version of the original double-digest restriction site-associated DNA (RAD) sequencing protocol^79^, as previously described^80^. The final libraries were spiked with 15% PhiX and sequenced on an Illumina NextSeq 500 platform using a 150-cycle paired-end chemistry (Illumina). A previously developed bash script pipeline^80^ was used to process the raw sequence reads. High-quality reads (Phred scores >25) trimmed to the 140-bp length were aligned to the AaegL5 reference genome (July 2017) using Bowtie v.0.12.7^62^. Parameters for the ungapped alignment included ≤3 mismatches in the seed, suppression of alignments with >1 best reported alignment under a ‘try-hard’ option. Variant and genotype calling was performed from a catalogue of RAD loci created with the ref_map.pl pipeline in Stacks v.1.19^81,82^. Downstream analyses only used high-quality genotypes at informative markers that were homozygous for alternative alleles in the F_0_ parents (for example, AA in the F_0_ male and BB in the F_0_ female), had a sequencing depth ≥10× and were present in ≥60% of the mapping population.

#### Linkage map

A comprehensive linkage map based on recombination fractions among RAD markers in the F_2_ generation was constructed using the R package OneMap v.2.0-3^83^. Every informative autosomal marker is expected to segregate in the F_2_ mapping population at a frequency of 25% for homozygous (AA and BB) genotypes and 50% for heterozygous (AB) genotypes. Autosomal markers that significantly deviated from these Mendelian segregation ratios (*P* < 0.05) were filtered out using a *χ*^2^ test. Owing to the presence of a dominant male-determining locus on chromosome 1, fully sex-linked markers on chromosome 1 are expected to segregate in F_2_ females with equal frequencies (50%) of heterozygous (AB) and F_0_ maternal (BB) genotypes, because the F_0_ paternal (AA) genotype only occurs in F_2_ males. As previously reported^21^, strong deviations from the expected Mendelian segregation ratios were observed for a large proportion of markers assigned to chromosome 1 in the female F_2_ progeny. Markers on chromosome 1 were included if they had heterozygous (AB) genotype frequencies inside the 40–60% range and F_0_ maternal (BB) genotype frequencies inside the 5%–65% range. These arbitrary boundaries for marker selection were largely permissive for partially or fully sex-linked markers on chromosome 1. Owing to a lack of linkage analysis methods that deal with sex-linked markers when only one sex is genotyped, the recombination fractions between all pairs of selected markers were estimated using the rf.2pts function with default parameters for all three chromosomes. The rf.2pts function, which implements the expectation–maximization (EM) algorithm, was used to estimate haplotype frequencies and recombination rates between markers^11^ under the assumption of autosomal Hardy–Weinberg proportions. Owing to this analytical assumption, the estimates of cM distances could be over- or underestimated for markers on chromosome 1. Markers linked with a LOD score ≥11 were assigned to the same linkage group. Linkage groups were assigned to the three distinct *Ae. aegypti* chromosomes based on the physical coordinates of the AaegL5 assembly. Recombination fractions were converted into genetic distances in cM using the Kosambi mapping function^84^. Linkage maps were exported in the R/qtl environment^85^ in which they were corrected for tight double crossing-overs with the calc.errorlod function based on a LOD cut-off threshold of 4. Duplicate markers with identical genotypes were removed with the findDupMarkers function. To remove markers located in highly repetitive sequences, RAD sequences were blasted against the AaegL5 assembly using BLASTn v.2.6.0. Markers with >1 blast hit on chromosomes over their 140-bp length and 100% identity were excluded from linkage analysis. Reported RAD markers were distributed as follows: chromosome 1, *n* = 76; chromosome 2, *n* = 80; chromosome 3, *n* = 99.

#### QTL mapping

The newly developed linkage map was used to detect and locate QTLs that underlie the DENV vector competence indices described above. Midgut infection was analysed in all F_2_ females whereas viral dissemination was analysed only in midgut-infected females. The four different DENV isolates were included as a covariate to detect QTL–isolate interactions. Single QTL detection was performed in the R/qtl environment^85^ using the expectation–maximization algorithm of the scanone function using a binary trait model. Genome-wide statistical significance was determined by empirical permutation tests, with 1,000 genotype–phenotype permutations of the entire dataset.

#### Comparison between AaegL5 and AaegL3

To assess the improvement obtained in AaegL5 to perform QTL mapping, a linkage map was built by aligning RAD markers to the AaegL3 assembly. The AaegL3-guided linkage map was built by assigning markers to chromosomes and by ordering them within each linkage group only on the basis of their recombination fractions. Markers were initially filtered based on their segregation ratios as described above and assigned to the same linkage group based on a LOD score threshold of ≥14. Linkage groups were assigned to the three *Ae. aegypti* chromosomes using supercontigs that were previously mapped to the chromosomes^22^. For each linkage group, a backbone was created with a small subset of informative markers (*n* = 6) using the rf.2pts two-point algorithm of the OneMap package. The remaining markers were positioned one at a time using the OneMap order.seq multi-point method, which compares all maps including the new marker at all possible positions keeping the original linkage map unchanged. This procedure produces both a ‘safe’ and a ‘forced’ marker order. The ‘forced’ marker map indicates the most likely position for each marker, whereas the ‘safe’ marker map only displays the unambiguously positioned markers. The AaegL3-guided QTL mapping was performed with the ‘safe’ marker map. Strong bias in Mendelian segregation ratios of markers anchored to chromosome 1 impeded their ordering. Fully sex-linked markers lacked the F_0_ paternal (AA) genotype in F_2_ females, and segregated analogously to a backcross design in which F_1_ AB heterozygotes are backcrossed to F_0_ BB homozygotes. No linkage analysis method is readily available to deal with a chromosome that behaves like a mixture of intercross and backcross designs. Therefore, AaegL3-guided linkage analysis and QTL mapping for chromosome 1 were restricted to the fully sex-linked region based on a backcross design. A new OneMap input file only including markers lacking the F_0_ paternal (AA) genotype was made by setting the population type to ‘backcross’ instead of ‘F2 intercross’. Markers were ordered using the order.seq function of the OneMap package as described above. A table summarizing this comparison is available as Extended Data Fig. 10b.

### Mapping insecticide resistance and VGSC

The mosquito population Viva Caucel from Yucatán State in Southern Mexico (longitude −89.71827, latitude 20.99827), was collected in 2011 through the Universidad Autónoma de Yucatán. We identified up to 25 larval breeding sites from 3–4 city blocks and collected around 1,000 larvae. Larvae were allowed to eclose, and twice a day we aspirated the adults from the cartons, discarding anything other than *Ae. aegypti*. Then, 300–400 *Ae. aegypti* were released into a 2-foot (61-cm) cubic cage where they were allowed to mate for up to five days with ad libitum access to sucrose, after which they were blood fed to collect eggs for the next generation. Then, 390 adult mosquitoes were phenotyped for deltamethrin resistance. We exposed groups of 50 mosquitoes (3–4 days old) to 3 μg of deltamethrin-coated bottles for 1 h. After this time, active mosquitoes were transferred to cardboard cups and placed into an incubator (28 °C and 70% humidity) for 4 h; these mosquitoes were referred to as the resistant group. Immobile mosquitoes were transferred to a second cardboard cup. After 4 h, newly recovered mosquitoes were aspirated, frozen and labelled as recovered; these were excluded from the current study. The mosquitoes that were immobilized and remained inactive at 4 h post-treatment were scored as susceptible. DNA was isolated from individual mosquitoes by the salt extraction method^86^ and resuspended in 150 μl of TE buffer (10 mM Tris-HCl, 1 mM EDTA pH 8.0). We constructed a total of four gDNA libraries. Two groups were pooled from DNA of 25 individual females that survived 1 h of deltamethrin exposure (resistant replicates 1 and 2). The second set of two libraries was obtained by pooling DNA from 25 females that were immobilized and inactive at 4 h post-treatment (susceptible replicates 1 and 2). Before pooling, DNA from each individual mosquito was quantified using the Quant-IT Pico Green kit (Life Technologies, Thermo Fisher Scientific) and around 40 ng from each individual DNA sample (25 individuals per library) was used for a final DNA pool of 1 μg. Pooled DNA was sheared and fragmented by sonication to obtain fragments between 300 and 500 bp (Covaris). We prepared one library for each of the four DNA pools following the Low Sample protocol from the Illumina TrueSeqDNA PCR-Free Sample preparation guide (Illumina). Because 65% of the *Ae. aegypti* genome consists of repetitive DNA, we performed an exome-capture hybridization to enrich for coding sequences using custom SeqCap EZ Developer probes (NimbleGen, Roche). Probes covered protein-coding sequences (not including untranslated regions) in the AaegL1.3 genebuild using previously specified exonic coordinates^87^. In total, 26.7 Mb of the genome (2%) was targeted for enrichment. TruSeq libraries were hybridized to the probes using the xGenLockDown recommendations (Integrated DNA Technologies). The targeted DNA was eluted and amplified (10–15 cycles) before being sequenced on one flow cell of a 100-bp HiSeq Rapid-duo paired-end sequencing run (Illumina) performed by the Centers for Disease Control (Atlanta, GA, USA).

The raw sequence files (FASTQ) for each pair-ended gDNA library were aligned to a custom reference physical map generated from the assembly AaegL5. Nucleotide counts were loaded into a contingency table with four rows corresponding to ‘Alive Rep1’, ‘Alive Rep2’, ‘Dead Rep1’ and ‘Dead Rep2’. The numbers of columns (*c*) corresponded to the number of alternative nucleotides at a SNP locus. The maximum value for *c* is 6, corresponding to A, C, G, T, insert or deletion. Three (2 × *c*) contingency tables were subjected to *χ*^2^ analyses (*c* − 1 degrees of freedom) to determine whether there are significant (*P* ≤ 0.05) differences between (1) Alive replicates, (2) Dead replicates and (3) Alive versus Dead. If analysis (1) or (2) was significant, then that SNP locus was discarded. Otherwise the third contingency table consisted of two rows corresponding to Alive (sum of replicates 1 and 2), Dead (replicates 1 and 2 summed), and *c* columns. The *χ*^2^ value from the (2 × *c*) contingency *χ*^2^ analysis with (*c* − 1) degrees of freedom was loaded into Excel to calculate the one-tailed probability of the *χ*^2^ distribution probability (*P*). This value was transformed with −log_10_(*P*). The experiment-wise error rate was then calculated following the method of Benjamini and Hochberg^88^ to lower the number of type I errors (false positives).

### Reporting summary

Further information on research design is available in the Nature Research Reporting Summary linked to this paper.

### Code availability

The overview of the Hi-C workflow, as well as modifications to 3D-DNA associated with AaegL5, is shared on GitHub at https://github.com/theaidenlab/AGWG-merge. The source code and executable version of Juicebox Assembly Tools are available at http://aidenlab.org/assembly. Data files and scripts used for the final polishing of scaffolded, gap-filled assembly are available at https://github.com/skingan/AaegL5_FinalPolish.

### Data availability

All raw data have been deposited at NCBI under the following BioProject accession numbers: PRJNA318737 (primary Pacific Biosciences data, Hi-C sequencing primary data and processed contact maps, whole-genome sequencing data from a single male (Fig. 4d) and pools of male and females (Fig. 3d), Bionano optical mapping data (Figs. 3c, 4c) and 10X linked-read sequences (Extended Data Fig. 8a and Supplementary Data 21)); PRJNA236239 (RNA-seq reads and de novo transcriptome assembly^13^ (Extended Data Fig. 2c and Supplementary Data 4, 5, 7, 9)); PRJNA209388 (RNA-seq reads for developmental time points^57^ (Fig. 1h and Supplementary Data 4–6, 9)); PRJNA419241 (RNA-seq reads from adult reproductive tissues and developmental time points, Verily Life Sciences (Fig. 1h and Supplementary Data 4, 5, 8, 9)); PRJNA393466 (full-length Pacific Biosciences Iso-Seq transcript sequencing); PRJNA418406 (ATAC-seq data from adult female brains at three points in the gonotrophic cycle (Extended Data Fig. 2c, d and data not shown)); PRJNA419379 (whole-genome sequencing data from four colonies (Fig. 4d and Extended Data Fig. 9a, b)); PRJNA399617 (restriction-site-associated DNA-sequencing data (Fig. 5a–d)); PRJNA393171 (exome-sequencing data (Fig. 5e-g)). Intermediate results related to the AaegL5 assembly are also available via GitHub (http://github.com/theaidenlab/AGWG-merge) and have been uploaded to GEO (GSE113256). The Hi-C maps are available via http://aidenlab.org/juicebox. The complete mitochondrial genome is available as Genbank accession MF194022.1, RefSeq accession NC_035159.1. The final genome assembly and annotation are available from the NCBI Assembly Resource under accession GCF_002204515.2.

## Online content

Any methods, additional references, Nature Research reporting summaries, source data, statements of data availability and associated accession codes are available at 10.1038/s41586-018-0692-z.

## Supplementary information

Supplementary Information This file contains the Supplementary Methods and Discussion

Reporting Summary

Supplementary Data This file contains Supplementary Data 1-24 and a detailed guide for the datasets
