# Supplementary material for: Improved reference genome of Aedes aegypti informs arbovirus vector control
Source: Nature. 2018 Nov 14;563(7732):501–7. doi: 10.1038/s41586-018-0692-z (PMC6421076; doi:10.1038/s41586-018-0692-z)
Supplement: Supplementary file 1 — This file contains the Supplementary Methods and Discussion [file 41586_2018_692_MOESM1_ESM.pdf]

nature

SUPPLEMENTARY INFORMATION

https://doi.org/10.1038/s41586-018-0692-z

In the format provided by the authors and unedited.

Improved reference genome of *Aedes aegypti* informs arbovirus vector control

Benjamin J. Matthews[1,2,3,49]*, Olga Dudchenko[4,5,6,7,49], Sarah B. Kingan[8,49], Sergey Koren[9], Igor Antoshechkin[10], Jacob E. Crawford[11], William J. Glassford[12], Margaret Herre[1,3], Seth N. Redmond[13,14], Noah H. Rose[15,16], Gareth D. Weedall[17,18], Yang Wu[19,20,21], Sanjit S. Batra[4,5,6], Carlos A. Brito-Sierra[22,23], Steven D. Buckingham[24], Corey L. Campbell[25], Saki Chan[26], Eric Cox[27], Benjamin R. Evans[28], Thanyalak Fansiri[29], Igor Filipović[30], Albin Fontaine[31,32,33,34], Andrea Gloria-Soria[28,35], Richard Hall[8], Vinita S. Joardar[27], Andrew K. Jones[36], Raissa G. G. Kay[37], Vamsi K. Kodali[27], Joyce Lee[26], Gareth J. Lycett[17], Sara N. Mitchell[11], Jill Muehling[8], Michael R. Murphy[27], Arina D. Omer[4,5,6], Frederick A. Partridge[24], Paul Peluso[8], Aviva Presser Aiden[4,5,38,39], Vidya Ramasamy[36], Gordana Rašić[30], Sourav Roy[40], Karla Saavedra-Rodriguez[25], Shruti Sharan[22,23], Atashi Sharma[21,41], Melissa Laird Smith[8], Joe Turner[42], Allison M. Weakley[11], Zhilei Zhao[15,16], Omar S. Akbari[43,44], William C. Black IV[25], Han Cao[26], Alistair C. Darby[42], Catherine A. Hill[22,23], J. Spencer Johnston[45], Terence D. Murphy[27], Alexander S. Raikhel[40], David B. Sattelle[24], Igor V. Sharakhov[21,41,46], Bradley J. White[11], Li Zhao[47], Erez Lieberman Aiden[4,5,6,7,13], Richard S. Mann[12], Louis Lambrechts[31,33], Jeffrey R. Powell[28], Maria V. Sharakhova[21,41,46], Zhijian Tu[20,21], Hugh M. Robertson[48], Carolyn S. McBride[15,16], Alex R. Hastie[26], Jonas Korlach[8], Daniel E. Neafsey[13,14], Adam M. Phillippy[9] & Leslie B. Vosshall[1,2,3]

[1]Laboratory of Neurogenetics and Behavior, The Rockefeller University, New York, NY, USA. [2]Howard Hughes Medical Institute, New York, NY, USA. [3]Kavli Neural Systems Institute, New York, NY, USA. [4]The Center for Genome Architecture, Baylor College of Medicine, Houston, TX, USA. [5]Department of Molecular and Human Genetics, Baylor College of Medicine, Houston, TX, USA. [6]Department of Computer Science, Rice University, Houston, TX, USA. [7]Center for Theoretical and Biological Physics, Rice University, Houston, TX, USA. [8]Pacific Biosciences, Menlo Park, CA, USA. [9]National Human Genome Research Institute, National Institutes of Health, Bethesda, MD, USA. [10]Division of Biology and Biological Engineering, California Institute of Technology, Pasadena, CA, USA. [11]Verily Life Sciences, South San Francisco, CA, USA. [12]Mortimer B. Zuckerman Mind Brain Behavior Institute, Department of Biochemistry and Molecular Biophysics, Columbia University, New York, NY, USA. [13]Broad Institute of MIT and Harvard, Cambridge, MA, USA. [14]Department of Immunology and Infectious Disease, Harvard T. H. Chan School of Public Health, Boston, MA, USA. [15]Department of Ecology and Evolutionary Biology, Princeton University, Princeton, NJ, USA. [16]Princeton Neuroscience Institute, Princeton University, Princeton, NJ, USA. [17]Vector Biology Department, Liverpool School of Tropical Medicine, Liverpool, UK. [18]Liverpool John Moores University, Liverpool, UK. [19]Department of Pathogen Biology, School of Public Health, Southern Medical University, Guangzhou, China. [20]Department of Biochemistry, Virginia Tech, Blacksburg, VA, USA. [21]Fralin Life Science Institute, Virginia Tech, Blacksburg, VA, USA. [22]Department of Entomology, Purdue University, West Lafayette, IN, USA. [23]Purdue Institute for Inflammation, Immunology and Infectious Disease, Purdue University, West Lafayette, IN, USA. [24]Centre for Respiratory Biology, UCL Respiratory, University College London, London, UK. [25]Department of Microbiology, Immunology and Pathology, Colorado State University, Fort Collins, CO, USA. [26]Bionano Genomics, San Diego, CA, USA. [27]National Center for Biotechnology Information, National Library of Medicine, National Institutes of Health, Bethesda, MD, USA. [28]Department of Ecology and Evolutionary Biology, Yale University, New Haven, CT, USA. [29]Vector Biology and Control Section, Department of Entomology, Armed Forces Research Institute of Medical Sciences (AFRIMS), Bangkok, Thailand. [30]Mosquito Control Laboratory, QIMR Berghofer Medical Research Institute, Brisbane, Queensland, Australia. [31]Insect-Virus Interactions Group, Department of Genomes and Genetics, Institut Pasteur, Paris, France. [32]Unité de Parasitologie et Entomologie, Département des Maladies Infectieuses, Institut de Recherche Biomédicale des Armées, Marseille, France. [33]Centre National de la Recherche Scientifique, Unité Mixte de Recherche 2000, Paris, France. [34]Aix Marseille Université, IRD, AP-HM, SSA, UMR Vecteurs – Infections Tropicales et Méditerranéennes (VITROME), IHU - Méditerranée Infection, Marseille, France. [35]The Connecticut Agricultural Experiment Station, New Haven, CT, USA. [36]Department of Biological and Medical Sciences, Faculty of Health and Life Sciences, Oxford Brookes University, Oxford, UK. [37]Department of Entomology, University of California Riverside, Riverside, CA, USA. [38]Department of Bioengineering, Rice University, Houston, TX, USA. [39]Department of Pediatrics, Texas Children's Hospital, Houston, TX, USA. [40]Department of Entomology, Center for Disease Vector Research and Institute for Integrative Genome Biology, University of California, Riverside, CA, USA. [41]Department of Entomology, Virginia Tech, Blacksburg, VA, USA. [42]Institute of Integrative Biology, University of Liverpool, Liverpool, UK. [43]Division of Biological Sciences, University of California, San Diego, La Jolla, CA, USA. [44]Tata Institute for Genetics and Society, University of California, San Diego, La Jolla, CA, USA. [45]Department of Entomology, Texas A&M University, College Station, TX, USA. [46]Laboratory of Ecology, Genetics and Environmental Protection, Tomsk State University, Tomsk, Russia. [47]Laboratory of Evolutionary Genetics and Genomics, The Rockefeller University, New York, NY, USA. [48]Department of Entomology, University of Illinois at Urbana-Champaign, Urbana, IL, USA. [49]These authors contributed equally: Benjamin J. Matthews, Olga Dudchenko, Sarah B. Kingan. *e-mail: bnmtthws@gmail.com

© 2018 Springer Nature Limited. All rights reserved.

SUPPLEMENTARY METHODS AND DISCUSSION

FALCON configuration file (‘Protocol.xml’)

[General]
input_fofn = input.fofn
input_type = raw
length_cutoff = -1
genome_size = 1800000000
seed_coverage = 30
length_cutoff_pr = 1000
sge_option_da = -pe smp 5 -q bigmem
sge_option_la = -pe smp 20 -q bigmem
sge_option_pda = -pe smp 6 -q bigmem
sge_option_pla = -pe smp 16 -q bigmem
sge_option_fc = -pe smp 24 -q bigmem
sge_option_cns = -pe smp 12 -q bigmem
pa_concurrent_jobs = 96
cns_concurrent_jobs = 96
ovlp_concurrent_jobs = 96
pa_HPCdaligner_option = -v -B128 -t16 -M32 -e.70 -l6400 -s100 -k18 -h480 -w8
ovlp_HPCdaligner_option = -v -B128 -M32 -h1024 -e.96 -l2400 -s100 -k24
pa_DBsplit_option = -x500 -s400
ovlp_DBsplit_option = -s400
falcon_sense_option = --output_multi --min_idt 0.70 --min_cov 2 --max_n_read 200 --n_core 8
falcon_sense_skip_contained = True
overlap_filtering_setting = --max_diff 100 --max_cov 100 --min_cov 2 --n_core 12

Comparison of assemblies and ‘treemap’ plots

The AaegL3 genome assembly (contigs) was downloaded from Vectorbase. AaegL4 was from NCBI GEO (accession GSE95797). Contig lengths were plotted using the ‘treemap’ package in R[1].

FALCON-Unzip assembly details

Half of the retained data were in reads of 16 kb or longer, with an average raw read length of 11.7 kb. We used raw reads 19 kb or longer as “seed reads” for error correction and generated 30.7 Gb (25.6X) of pre-assembled reads (preads) for contig assembly[2,3]. The resulting contig assembly contained primary contigs, comprising the backbone of the genome, and associated haplotigs, which represent phased, alternate haplotypes.

Hi-C scaffolding and de-duplication

The Hi-C scaffolding procedure used both primary contigs and haplotigs from the FALCON-Unzip assembly as input. Here undercollapsed heterozygosity was apparent. In fact, most genomic intervals were repeated, with variations, on 2 or more unmerged contigs, resulting in the ‘true’ genome length as measured by flow cytometry being far shorter than the total length of the FALCON-Unzip contigs (2,047 Mb).

The workflow for scaffolding and alternative haplotype removal was based on 3D *De Novo* Assembly (3D-DNA) pipeline introduced in Dudchenko et al.[4] with algorithmic modifications and manual curation via Juicebox Assembly Tools[5] to address the difficulties associated with exceptionally high levels of undercollapsed heterozygosity in the draft.

The overview of the workflow, as well as modifications to 3D-DNA associated with AaegL5, is shared on GitHub at https://github.com/theaidenlab/AGWG-merge. The source code and executable version of Juicebox Assembly Tools are available at http://aidenlab.org/assembly. Intermediate results relating to each assembly step are also available at AGWG-merge project on GitHub and have been uploaded to GEO (BioProject PRJNA318737, GEO Record GSE113256). All shared files can be viewed via Juicebox Assembly Tools[5]. In the GitHub overview, we also include interactive links to the shared files for examination in the cloud-based, installation-free visualization system Juicebox.js[6].

In brief, the workflow started with the draft FALCON-Unzip assembly fasta (AGWG.draft.fasta.gz) and the duplicate-free list of paired alignments of Hi-C reads to the draft (AGWG.draft.mnd.txt.gz) as generated by the Juicer pipeline[7]. The input files as well as contact maps and 2D annotation files associated with the FALCON-Unzip output fasta are shared at https://github.com/theaidenlab/AGWG-merge#step-0-draft-contigs.

The draft was subject to preliminary filtration, in which a set of contigs less than 20,000 bases long is removed from the draft. Due to their small size, these contigs have relatively few Hi-C contacts, making them more difficult to reliably analyse. These ‘tiny’ contigs (smaller than 20,000 bases) are set aside and kept without any modification. The remaining contigs are then ordered and oriented using automatic 3D-DNA scaffolding algorithm[4]. The code, *.assembly*, *.hic* and 2D annotation files associated with this step are shared at https://github.com/theaidenlab/AGWG-merge#step-1-preliminary-scaffolding.

Analogous to the 3D-DNA workflow, the resulting contact map was examined for evidence of misjoins. The 3D-DNA automatic method for misjoin detection using Hi-C typically relies on the fact that sequences that have been erroneously concatenated form fewer contacts with one another than correctly joined sequences. In the case of AaegL5 however this signal is confounded by alignment biases associated with the presence of multiple haplotypes. As a result, misassembly detection was performed with manual curation. The manually curated list of inconsistent regions is shared at https://github.com/theaidenlab/AGWG-merge#step-2-curated-misjoin-correction. Inconsistent regions were excised from draft contigs and labelled ‘debris’, much like in the case of automatic misassembly detection in 3D-DNA.

The edited scaffolds were then again ordered and oriented automatically using 3D-DNA scaffold module, see https://github.com/theaidenlab/AGWG-merge#step-3-automatic-scaffolding-of-misjoin-corrected-contigs for command details and intermediate files associated with this step.

The resulting assembly was manually polished using Juicebox Assembly Tools[5], to remove false positive scaffold joins associated with telomere clustering characteristic of *Ae. aegypti*[4]. At the same time, telomere-to-telomere signal was exploited to identify chromosome boundaries[4]. For convenience, chromosomes were ordered according to convention, with chromosome 1 the shortest, chromosome 2 the longest, and chromosome 3 of medium length, and oriented based on linkage data and comparison with AaegL4[4]. The review file associated with Juicebox Assembly Tools modifications as well as other intermediate files associated with this step are shared at https://github.com/theaidenlab/AGWG-merge#step-4-curated-polishing-

and-chromosome-splitting. The modifications encoded in AGWG.1.review.assembly can be reviewed using the 'Load modified assembly' menu option in Juicebox Assembly Tools.

As a result of the preceding steps most of the consistent, misjoin-free contigs longer than 20 kb have been 'resolved' i.e. placed into one of the three 'raw chromosomal scaffolds.' The remainder of the Hi-C-based procedure aims to remove long duplicate sequences associated with undercollapsed heterozygosity, i.e. when two or more contigs correspond to a single locus in the haploid genome.

The main premise for undercollapsed heterozygosity error-correction using Hi-C is that, when multiple contigs correspond to alternative haplotypes, the contigs will, in addition to long stretches of high sequence identity, display extremely similar contact patterns genome-wide, leading to their incorporation to nearby positions in the assembly based on their 3D contact signal[4].

Hence, just as in prior work[4], we searched for undercollapsed loci by running a sliding window of fixed width along the raw chromosomal scaffolds. We then used LASTZ[8] to do pairwise alignment of all resolved contigs that fall in the sliding window. The alignment score, overlap length and sequence identity, and the location of the overlap relative to the boundaries of the input contigs are used as filtering criteria to distinguish between alternative haplotypes and false positive sequence similarity[4].

The parameters for running the merge in the case of AaegL5 had to be considerably more permissive as compared to those used for AaegL4[4] to allow for identification of more divergent overlaps (due to incomplete inbreeding) separated by larger genomic distances (due to longer contig sizes). We found that running the merge pipeline with such permissive parameters occasionally results in false positives and merges contigs that do not overlap.

To avoid this, we added a manual review step into the procedure. Specifically, the alternative haplotype removal process can be thought of as consisting of three consecutive steps: (i) pairwise alignment of nearby contigs; (ii) identification of alignment chains, and ordering and orientation of contigs within chains based on alignment data (we refer to this procedure as tiling); and (iii) merging of chained sequences into haploid contigs. The review consisted of surveying the alignment chains from step (ii).

In practice, this entailed loading Hi-C maps (mapq≥0) into Juicebox Assembly Tools while overlaying alignment data in the form of 2D annotations constructed from LASTZ output. Note that reviewing the map and 2D annotations in Juicebox Assembly Tools allows for two independent sources for confirming high sequence identity at two genomic intervals: one from short-read (mis)alignment and coverage, and another one from pairwise contig sequence similarity. The commands and intermediate files associated with pairwise alignment and automatic tiling are shared at https://github.com/theaidenlab/AGWG-merge#step-5-automatic-pairwise-alignment-of-nearby-contigs.

The automatically identified chains of overlapping contigs – merge blocks – have been manually curated using Assembly Tools to break down chains from false positive alignments, remove ambiguously aligning contigs and review ordering and orientation of merge blocks as decided by the component majority vote[4]. Note that merge is allowed between contig sequences in the same blocks but not between contigs belonging to different blocks. For files associated with this step see https://github.com/theaidenlab/AGWG-merge#step-6-curation-of-merge-groups, in particular AGWG.rawchrom_tiled.review.assembly that can be surveyed using the 'Load modified assembly' menu option in Juicebox Assembly Tools.

Merging of chained sequences based on alignment data was performed iteratively as previously described[4], and the commands and results associated with this step are available at https://github.com/theaidenlab/AGWG-merge#step-7-automatic-merging-of-overlapping-contigs. A new script has been added to the merge portion of the 3D-DNA pipeline in order to facilitate comparison of iterative and pairwise alignments as well as classify individual contig contributions to the final haploid reference (lift-merged-annotations-to-unmerged-map-overlaps.awk, available via AGWG-merge GitHub project).

In addition to the files referenced above we also share Hi-C contact maps generated using the final AaegL5 assembly as a reference. On top of Hi-C based anchoring, ordering, orientation and removal of long stretches of undercollapsed heterozygosity AaegL5 includes additional polishing and gap filling steps, see below. The heatmaps, shared at https://github.com/theaidenlab/AGWG-merge#step-8-final-ncbi-submission, indicate the frequency of contacts between pairs of loci in AaegL5 at multiple resolutions as measured by three Hi-C experiments conducted for this study (NCBI Accession Numbers SRX3395766, SRX3395767, SRX3395768), both separately and combined.

Gap filling protocol

After Hi-C scaffolding and de-duplication, all 527 Pacific Biosciences subread .fastq files were used as input to PBJelly for final polishing and gap-filling. The format for each input is denoted by the bold and italicized lines below (replace ***XXX_N.subreads.fastq*** with the full name of each file).

```
<jellyProtocol>
 <reference>asm.fasta</reference>
 <outputDir>./</outputDir>
 <cluster>
 <command notes="For SGE">echo '${CMD}' | qsub -V -N "${JOBNAME}" -cwd -pe thread 8
-l mem_free=4G -o ${STDOUT} -e ${STDERR}</command>
 <nJobs>100</nJobs>
 </cluster>
 <blasr>--minMatch 8 --minPctIdentity 70 --bestn 1 --nCandidates 20 --maxScore -500 --nproc 8
--noSplitSubreads</blasr>
 <input baseDir="/seq/a_aegypti/pacbio/">
        <job>XXX_1.subreads.fastq</job>
        …
        <job>XXX_527.subreads.fastq</job>
 </input>
</jellyProtocol>
```

BUSCO completeness

BUSCO, a benchmark based on single-copy universal orthologues[9] was used to confirm that multiple haplotypes were present in the initial assembly and to evaluate the success of our de-duplication. BUSCO contains a database of genes that are thought to be present in single-copy in all species below a given taxonomic level. Thus, any complete assembly should include all or close to all of these genes. Since all these genes are also single-copy there should not be any duplicated genes in an assembly. Duplicate genes indicate potential alternate haplotypes present

in the assembly result. AaegL2 and L3 are community updates derived from the original LVP assembly[10] and were downloaded from VectorBase, AaegL4[ref. 4] was obtained from NCBI.

BUSCO v3.0.2 was run with default parameters and the dipteran geneset with the command:

```
run_BUSCO.py -c 16 --blast_single_core -f --in asm.fasta -o SAMPLE -l diptera_odb9 -m
```

which yielded the following results for complete (C), single-copy (S), duplicated (D), fragmented (F), and missing (M) BUSCO genes (n=2799):

Falcon	C: 97.7% [S:46.3%, D:51.4%], F:1.1%, M:1.2%
AaegL2	C: 96.4% [S:91.1%, D:5.3%], F:2.0%, M:1.6%
AaegL3	C: 96.4% [S:91.1%, D:5.3%], F:2.0%, M:1.6%
AaegL4	C: 95.4% [S:93.1%, D:2.3%], F:2.4%, M:2.2%
AaegL5	C: 96.7% [S:93.0%, D:3.7%], F:1.8%, M:1.5%

Due to its comprehensive representation of alternate alleles, the initial FALCON assembly had the highest fraction of complete BUSCO genes. However, as expected, this assembly also had a high rate of duplicated genes (51.4%). These duplications were effectively removed via de-duplication to a rate comparable to the previous reference genomes (3.7%). After de-duplication, the L5 assembly still scored slightly higher than the prior references in the per cent of complete BUSCO genes (96.7%) and the numbers of fragmented (1.8%) and missing (1.5%) genes were also reduced.

Challenges in addressing structural and base-level accuracy of AaegL5

Precise calculation of base-level and structural accuracy in AaegL5 was challenging because the assembly was generated from material gathered from 80 siblings with an unknown level of residual heterozygosity. Comparing AaegL5 to the existing reference genome (AaegL4) to assess quality is not relevant because these genomes derive from different strains, and there is a high degree of natural diversity between *Ae. aegypti* strains. In fact, only 70% of the older AaegL4 reference aligns to the new AaegL5 assembly with >95% identity.

As for QV estimation using short-read Illumina data from a single individual (see Methods), we would predict it to underestimate quality, not overestimate, for the following reasons. First, the Illumina data were not used in the assembly and represent an independent quality measurement. Second, the vast majority (>95%) of assembled bases were covered by at least 3x Illumina coverage and thus included in the QV estimate. Third, we used a conservative strategy for calling variants between the assembly and the Illumina data. In short, any alternate allele called by FreeBayes was considered an “error” even if there was Illumina support for the assembled allele. Thus, we view the base-level accuracy as calculated (QV 34.75) as a lower bound on the consensus quality because it considers all allelic discrepancies between the PacBio assembly and independent Illumina data as errors. As for possible enrichment of errors in certain sequence contexts, it has also been shown that heterozygous sequence can confuse the PacBio polishing process and introduce indel errors. Thus, we would expect allelic differences to be the primary source of error, but the vast majority of these errors should be captured by the Illumina variant detection described above. The improved BUSCO gene representation and gene set

annotation further support the high base-level accuracy of the assembly, especially when viewed in comparison to the highly fragmented and incomplete prior reference assemblies.

Calculating structural accuracy of the AaegL5 assembly is similarly difficult, due to the heterogeneous input material from multiple individuals used to generate the primary assembly, the 10X linked-read libraries, and Bionano optical maps. The mapping of BAC clones by FISH shows that the assembly is highly accurate at a gross scale. Bionano data and 10X linked-reads support the accuracy of specific loci examined in detail, such as the M-locus (Fig. 3) and the GSTe gene cluster (Fig. 4), but also suggests the presence of structural variants in the population of mosquitoes used to generate these data (Extended Data Fig. 2a and Extended Data Fig. 8a). Future studies investigating natural variation of *Ae. aegypti* at the structural and sequence level, within and between strains, will be extremely important and AaegL5 will provide the foundation for this work.

Analysis of transposable elements

We ran RepeatMasker using the TEfam and Repbase databases, and found transposable elements represent 54.85% (excluding the 3.02% unclassified TEs) of the new assembled genome. Moreover, 25.48% of the total base pairs identified as TEs were DNA elements, 28.92% were RNA elements, and 0.45% were Penelope (Supplementary Data 2-3). Simple and tandem repeats occupy 3.3% of the genome, and the additional 7% consists of unclassified repetitive sequences. Similar to previous annotation, *Juan-A* in the Jockey family of non-long terminal repeat (non-LTR) retrotransposons is the most enriched TE type, accounting for ~3.4% of the genome. In general, the percentage of previously identified TEs is consistent with the 2007[10] genome, except that P Instability Factor (PIF), a DNA transposable element, increased from 1.19% to 2.85%. Using Tandem Repeat Finder, we found that 6.9% of genome sequences are repeat sequences, while 1% of the genome is simple repeat sequences. Since a subset of the tandem repeat sequences overlap with TE regions, we then used tandem repeat finder to search for repeatmasked genome sequences and found that the whole genome contains 3.3% non-TE tandem repeat sequences.

We identified a significant positive correlation between GC content and the total lengths of TEs (Pearson's $r = 0.37$, $p < 0.001$) of each chromosome or scaffold. However, there is not a significant correlation between the number of TEs and GC content (Pearson's $r = -0.02$, $p > 0.05$). Compared to previous TEfam annotation, new transposon elements such as CMC-Chapaev, CMC-Transib, sola, and Crypton showed relatively high copy numbers. Overall, a greater proportion of TE sequences belong to DNA elements compared to the previous annotation. Our results of TE identification using different libraries suggest that novel TE types are the main contributor to the higher proportion of DNA elements (Supplementary Data 2-3). However, it is difficult to directly compare these results with AaegL3 (ref. [10]), since different TE elements may have different lengths and numbers of insertions, and many different element types have high sequence similarities. A file representing the coordinates of all identified repeat and transposable-element structures in AaegL5 can be found at https://github.com/VosshallLab/AGWG-AaegL5.

Definitive identification of transcription start sites

Future work to further improve the AaegL5.0 genome may include cap-analysis gene expression (CAGE)[11] or RNA ligase-mediated rapid amplification of 5′ cDNA ends (5′-RACE)[12] sequencing experiments to definitively identify transcription start sites.

10X genomics library preparation and Illumina sequencing for analysis of structural variants (SV)

Two individual pupae, one male and one female, were selected from the first generation of inbreeding of the LVP_AGWG strain (Extended Data Fig. 1a). High molecular weight (HMW) genomic DNA was extracted using the Qiagen MagAttract kit according to manufacturer's instructions with minor modifications (rapid vortexing was replaced by inversion and wide-bore pipette tips were used – both to prevent excessive shearing of DNA). DNA extracted from each individual pupa was loaded into a separate lane of a 10X Chromium instrument for barcode tagging of the amplicons and an Illumina sequencing library was prepared. Each library was sequenced in duplicate on two lanes of an Illumina HiSeq 2500. Due to the potential for transposable elements to give false positive SV calls, the AaegL5 genome was hard masked using RepeatMasker 4.05[13] using the Aaeg-Liverpool V1 repeat library. Unplaced primary scaffolds and secondary haplotypes (i.e. any scaffolds or contigs except chromosomes 1, 2 and 3) were not used for alignment. Sequences were aligned to the reference using BWA via the LongRanger-Align function. Variants were called using GATK HaplotypeCaller (GATK version 3.5.0)[14] and filtered for quality (QD > 5), strand bias (FS < 60) and read position (RankSum < 8). Only biallelic SNPs were used for phasing and subsequent analyses. The full Longranger-WGS pipeline was run on each sample (Longranger v.2.1.5) with memory overrides for both the SNP/INDEL phasing and SV calling stages required due to the high heterozygosity found in these samples. The pipeline was run with the pre-called VCF from the prior variant calling ensuring that the same sites were genotyped and phased in all samples. A second SV calling pipeline, GROCSVs, was run on the BWA alignments generated for variant calling. Repeat regions detected by RepeatMasker were blacklisted ensuring that no SVs would be called within these regions. SVs were compared between each pair of technical replicates and both methods; SVs under 30 kb were not reported due to limited sensitivity of SV callers below the mean molecule size. SVs were compared based on position and merged if they showed a 95% pairwise overlap. Only structural variants that were found in both technical replicates for a sample were reported (Extended Data Fig. 8a and Supplementary Data 21).

Identification of *Ae. aegypti Hox* genes and *Hox* sequence alignment

Ae. aegypti Hox cluster (*HOXC*) genes were identified by utilizing BLASTP2.6.1+[15] to search the *Ae. aegypti* genome for genes with high similarity to *D. melanogaster HOXC* genes. The identity of *Aedes HOXC* genes was further resolved by comparing the relative position of candidate genes within the *HOXC*. The sequences of *HOXC* genes in *D. melanogaster* (annotation version R6.17) and *D. virilis* (annotation version R.106) were retrieved from Flybase, www.flybase.org[16]. The sequences of *HOXC* genes in *An. gambiae* (PEST annotation, version AgamP4.4) were retrieved from VectorBase, www.vectorbase.org[17]. Predicted coding exons for all *Hox* genes were aligned with the full *HOXC* genomic region using GenePalette www.genepalette.org[18], then each species' *HOXC* were proportionally adjusted to scale in Adobe Illustrator. The tandem repeats adjacent to *pb* were identified using GenePalette to search for regularly-spaced sequences. Six identical 749 bp tandem repeats were discovered on the end of Chromosome 1q in *Ae. aegypti* AaegL5, related to telomere-associated sequences in species without telomeres[19]. Similar repeats of 556 bp were found at the same position at the tip of chromosome 1q in *Cx. quinquefasciatus* genome assembly CpipJ3[4] and on the tip of chromosome 3p of *Ae. aegypti* AaegL5. To compare the Hox-Extradenticle (Exd[21]) interaction

motifs, Hox protein sequences were aligned using Clustal-Omega[20] (Extended Data Fig, 8b-d and Supplementary Data 22).

Evidence supporting the split of *proboscipedia* (*pb*) and *labial* (*lb*) in *Ae. aegypti* is the presence of long tandem repetitive sequences neighbouring *pb* in both *Ae. aegypti* and *Cx. quinquefasciatus*, reminiscent of telomere-associated sequences in species that lack telomerase[19] (Extended Data Fig. 8c). We examined the presence of motifs known to mediate protein-protein interactions with the Hox cofactor Exd[21]. Most Hox proteins bind Exd using the canonical "YPWM" motif, but in *D. melanogaster* the abdominal Hox proteins Ultrabithorax (Ubx) and Abdominal-A (Abd-A) have additional "W" motifs that may be utilized in a context-dependent manner[21]. The *Ae. aegypti* Hox proteins have all previously described "W" motifs (Extended Data Fig. 8d). In all three species of mosquito analysed here, Abdominal-B (AbdB) has as an additional putative Exd interaction motif, "YPWG", which closely resembles the canonical "YPWM" motif in other *D. melanogaster* Hox proteins (Extended Data Fig. 8d).

Curation of proteases

First, we identified 404 genes annotated as serine proteases (proteases, proteinases, peptidases, trypsins and chymotrypsins) and metalloproteases (metalloproteases, metalloproteinases and metametallopeptidases) in AaegL3.4, based on conserved domains. The UniProt database was searched to confirm serine protease/metalloprotease molecular function. We mapped these transcripts against the AaegL5.0 geneset annotation by taking the longest transcript and using the reciprocal best BLAST method. We extracted the coding sequence (CDS) lengths and corresponding peptide lengths for each of the transcripts for each of the 404 genes, from both AaegL3.4 and AaegL5.0. This comparison showed that over 50% of the gene models corresponding to the two protease subclasses have been changed in AaegL5.0 (Supplementary Data 13). This does not include the change in the UTRs. Twenty-one of the previous models have been discontinued. We also analysed 49 more gene models that are annotated as serine proteases or metalloproteases in AaegL5.0 but not in AaegL3.4 and were able to map all of these back to AaegL3.4 gene models by reciprocal best BLAST. These genes were either not annotated or not identified as proteases in AaegL3.4.

Curation of opsins and biogenic amine binding G protein-coupled receptors

Genes for the opsin and biogenic amine binding Class A G protein-coupled receptor (GPCR) superfamily were identified by TBLASTN searches against the *Ae. aegypti* AaegL5 genome assembly and manually annotated as previously described using multiple online databases and software [10,17,20,22-27]. The resulting gene models were assigned to putative functional classes on the basis of sequence homology to multiple vertebrate and invertebrate GPCRs that have been functionally characterized. Results are summarized in Extended Data Fig. 2f and Supplementary Data 14-16. Notably, the AaegL5 assembly enabled the prediction of the first full-length gene models for *GPRop10* and *GPRop12*, and 14 biogenic amine-binding receptors. The majority of curations involved the addition of 5' sequence, and the consolidation of models for biogenic amine binding receptors from 26 to 17 via collapse and resolution of AaegL3-derived gene models. In all, genes for 10 opsin and 17 biogenic amine-binding receptors were annotated (3 dopamine; 8 serotonin: 2 muscarinic acetylcholine; 3 octopamine/tyramine receptors; 1 "unclassified" Class A biogenic amine binding). The AaegL5 assembly enabled the first full-length gene model predictions for two opsin (*GPRop10* and *12*) and 14 biogenic amine binding (*GPRdop1* and *2*, *GPR5HT1A*, *1B* and *2*, *putative 5HT receptor 1-3*, *GPRmac1* and *2*, *GPRoar 1*,

2 and *4*, and *GPRnna19*) GPCRs, with consolidation of dopamine receptors from six to three, serotonin receptors from 11 to eight, muscarinic acetylcholine receptors from three to two, and octopamine/tyramine receptors from six to three by fusion and resolution of the AaegL3-derived models described in Nene et al., 2007[10]. We discovered two isoforms of the *GPRdop1* (X1 and X2) dopamine and *GPRoar1* (X1 and X2) receptors that respectively possess N-terminal and internal regions unique from that predicted for the AaegL3 models. The chromosome-level resolution of the AaegL5 assembly confirms the previously reported *GPRop1-5* cluster on chromosome 3[27], which has been suggested to be a duplication event associated with adaptation of mosquitoes to new visual environments.

We identified and manually annotated three dopamine receptors (*GPRdop1-3*, previously reported[10] and subsequently characterized[25]), eight putative serotonin receptors (*GPR5HT*, with one, *GPR5HT7A*, previously characterized[22]), two muscarinic acetylcholine receptors (*GPRmac1* and *2*) and three previously reported octopamine/tyramine receptors (*GPRoar1, GPRoar2*, and *GPRoar4*)[10,28] in the AaegL5 assembly. We made a considerable revision to *GPRdop3* with the addition of 241 amino acids to the 5' region of the model and the inclusion of a short 11th exon (33 amino acids), increasing the total number of exons for this gene model from eight to 12. We discovered two isoforms of *GPRdop1* (X1 and X2) and *GPRoar1* (X1 and X2) that respectively possess N-terminal and internal regions unique from that predicted for the AaegL3 models.

The AaegL5 assembly enabled greater resolution of the serotonin or 5-hydroxytryptamine (5HT) receptor subfamily, comprising eight members (*GPR5HT1A*, *GPR5HT1B, GPR5HT2, GPR5HT7A, GPR5HT7B* and *putative 5HT receptors 1-3*) with prediction of the first full-length gene model for *GPR5HT1A* (80 amino acids added to the 5' region) and substantial revision to *GPR5HT1B* (addition of 86 amino acids, representing revision of sequence corresponding to exons 2, 5 and 6). The AaegL5.0 annotation also enabled major revision of *GPR5HT2* with the addition of 292 and 115 amino acids to the 5' and internal regions of the model, respectively.

The remaining three receptors designated as *putative 5HT receptor 1-3* possess some sequence homology to vertebrate and invertebrate serotonin receptors. The AaegL3.4 gene models corresponding to these receptors comprise one or more exons with high amino acid similarity to vertebrate and invertebrate serotonin receptors, but lack 5' and 3' sequence and are considered incomplete. The revised AaegL5.0 gene models incorporated additional 5' and/or 3' sequence and each model comprises critical features inclusive of an initiation methionine, stop codon, seven transmembrane spanning domains, and canonical GPCR motifs such as N-terminal glycosylation and C-terminal palmitoylation sites. These three models are supported by RNA-seq data and possess homology to orthologous serotonin receptors identified in vertebrate and invertebrate species. However, some ambiguity remains. The predicted protein sequence of the putative *GPR5HT receptor 2* is considerably longer than that of many GPCRs, for example. These models will require resolution via molecular analyses.

Two muscarinic acetylcholine receptors (*GPRmac1* and *GPRmac2*) were identified using the AaegL5 assembly and possessed high amino acid identity to the AaegL3-derived models. The AaegL5 assembly also enabled greater resolution of the *Ae. aegypti* octopamine receptor subfamily (four complete gene models for *GPRoar1, GPRoar2*, and *GPRoar4*). Key advances include the prediction of two isoforms for *GPRoar1* (X1 and X2; addition of 149 and 141 amino acids to the 5' region of X1 and X2, respectively) and the addition of a total 141 and 211 amino acids to the models for *GPRoar 2* and *4*, plus the deletion of 19 amino acids from *GPRoar4*.

Our analyses revealed several gene models for receptors that had been renamed between the AaegL3.4 and AaegL5.0 genesets; specifically, *GPR5HT2* to a muscarinic M3 receptor (the

original gene name was retained here) and *AaGPR5HT8* to an "uncharacterized protein" (subsequently renamed here as "*putative 5HT receptor 2*"). In the interest of consistency, the current analyses attempted to resolve these discrepancies (Supplementary Data 14) based on multiple lines of evidence, including RNA-seq data, manual annotation, and sequence homology to functionally characterized GPCRs from vertebrates and invertebrates. Several instances of gene model collapse were identified between the AaegL3 and AaegL5 genesets (AAEL014373 and AAEL017166 into LOC5564275 for *GPRdop3*; AAEL09573 and AAEL016993 into LOC5572158 for *GPR5HT7A*; AAEL015553 and AAEL002717 into LOC5575783 for *putative 5HT receptor 2*). We also note that multiple transcript variants were detected for *GPR5HT2* (LOC23687582; 6 variants), *GPR5HT7A* (LOC5572158; 4 variants) and *putative 5HT receptor 2* (LOC5575783; 14 variants). These variants were predicted to produce gene products with identical amino acid sequence and their status as haplotype sequence is yet to be resolved. Finally, one previously reported sequence (*GPRnna19*) identified as a putative biogenic amine binding receptor in the AaegL3.4 geneset was renamed in the AaegL5.0 geneset as a "putative tyramine/octopamine receptor". The AaegL5.0 model includes an additional 142 amino acids of 5' sequence, and is supported by RNA-seq data and sequence homology to biogenic amine binding GPCRs. However, membrane prediction software suggests that this model comprises only three or four transmembrane spanning domains and it lacks amino acid motifs considered critical to GPCR function. The gene was not assigned to subfamily in the present analysis and the model was designated as "unclassified" (Supplementary Data 14). Molecular studies will be needed to confirm the model. Finally, we note that the majority of GPCRs identified in the present analyses should be considered "orphan" receptors. Functional studies will be required for all but *GPRdop1, dop2,* and *5HT7A* to establish receptor activity and interaction with the cognate ligand(s).

Ten previously reported opsins (*GPRop1-5, GPRop7-10,* and *GPRop12*)[10,27], were identified in the AaegL5 assembly, and sequence was confirmed via manual annotation. The opsins represent a gene family (typically 3-7 receptors in arthropods) of UV-, short- and long-wavelength sensitive receptors and have been annotated in many arthropods. Ten, 11 and 13 *opsin* genes were identified in the mosquitoes *Ae. aegypti, An. gambiae*, and *Cx. quinquefasciatus*, respectively[27] and thus provide an opportunity to benchmark the AaegL5.0 annotation. All AaegL5.0 *opsins* were full-length and the predicted gene products possessed features indicative of functional GPCRs, including an initiation methionine, a stop codon, seven transmembrane domains, three extracellular and three intracellular loops, as well as conserved motifs associated with GPCR and opsin function (except for *GPRop10* which contained six transmembrane domains). Non-synonymous substitutions were identified in *AaegGPRop2, AaegGPRop7,* and *AaegGPRop10* in regions not typically considered critical for functions such as photon interaction, amine binding or G protein-coupling. AaegL5.0 enabled prediction of the 5' coding region for *AaGPRop12* and the development of a potentially full-length gene model, representing a major advance over the AaegL3.4 annotation. The chromosome-level resolution of the AaegL5 assembly confirms the previously reported *GPRop1-5* cluster on chromosome 3[27], which has been suggested to be a duplication event associated with adaptation of mosquitoes to new visual environments. Visualizations of the gene models described above are presented as Extended Data Fig. 2f.

Curation of chemosensory receptors

Annotation of previously identified genes We used previously published *Ae. aegypti* (hereafter *Aaeg*) odorant receptor (*OR*), gustatory receptor (*GR*), and ionotropic receptor (*IR*) sequences[29-31] as queries to locate these genes in the new assembly. TBLASTN[32] was used for protein sequences and discontinuous MegaBLAST[33] followed by GMAP[34] was used for coding sequences. New gene models were built, or NCBI RefSeq models accepted/modified, in the corresponding locations in an Apollo v2 browser[26]. Most modifications of RefSeq or previously published models were based on supporting RNA-seq data[28]. These RNA-seq data were derived from key chemosensory tissues in adult males and females and were loaded into Apollo as short read alignments for each tissue (raw data available in the NCBI SRA database) and as alignments of *de novo* assembled transcripts prefiltered for those with TBLASTN homology to published chemoreceptors (TBLASTN e-value $<10^{-10}$; *de novo* transcriptome available in the NCBI TSA database). We paid particular attention to pre-existing genes that were recognized by previous authors as fragments or were simply outside the normal length range for receptors in each of the three families. In most cases, we were able to extend these genes to full-length using GENEWISE[35] with closely related receptor proteins as queries or by manual assessment of TBLASTN homology of flanking sequences to related receptors. For *ORs* and *IRs*, we used a reciprocal discontinuous MegaBLAST[33] to verify that the coding sequences we annotated in AaegL5 corresponded to specific previously identified genes. This was necessary due to varying levels of sequence divergence between alleles found in AaegL3 and AaegL5. Moreover, we found many cases where two previously identified genes from AaegL3 mapped to the same locus in AaegL5, likely representing alternative haplotypes erroneously included on separate contigs in AaegL3 (classified as 'merged' genes in Fig. 2b and Supplementary Data 17). We note that although no geneset annotation exists, AaegL4[4], which de-duplicated and scaffolded AaegL3 onto chromosomes, could be used to confirm these 'merges' as well.

Search for new genes in AaegL5 We also searched AaegL5 for new genes using the same TBLASTN results used to locate previously known genes (searches of AaegL5 with known chemoreceptor proteins). For new *ORs*, we manually examined all TBLASTN hits with an e-value cutoff below 10^{-10} after filtering for overlap with previously annotated genes using BedTOOLS[36]. For new *IRs*, we did the same but lowered the e-value cutoff to 10^{-50} to exclude ionotropic glutamate receptors (*iGluRs*), which have high homology to *IRs*[31]. This approach identified a handful of new *IR* genes that were then used to query the *An. gambiae* genome (AgamP4) with a much more liberal TBLASTN e-value threshold of 1000 (*iGluRs* were ignored). Resulting discoveries in *An. gambiae* were then used to requery AaegL5 with the same liberal threshold and so forth in an iterative process until no new hits were identified. For *GRs*, we used proteins from *An. gambiae*[37,38] and *D. melanogaster*[39,40] as TBLASTN queries in addition to pre-existing *Ae. aegypti* proteins, and manually examined any hits with e-values below 1000. For all three families, instances of apparent loss of an *Ae. aegypti* chemoreceptor suggested by the tree analyses were checked by searching the NCBI transcriptome shotgun assembly database for *Ae. aegypti* with TBLASTN using the relevant *Cx. quinquefasciatus*, *An. gambiae*, or *D. melanogaster* protein as query. For many chemoreceptors, these searches of the transcripts should allow detection of more divergent proteins because they are longer than the shorter exons in the genome and independent of the genome assembly. However, no new genes were discovered by searches of the transcripts that had not been found in the genome, indicating that our compilations of these three chemoreceptor families are likely exhaustive. We checked whether newly identified genes were missing in the old assembly or present but simply not recognized as receptors/genes. We used BLASTN[32] to query the AaegL3 assembly with the new

receptor genes and BedTOOLS[36] to exclude hits to previously identified receptors. We considered a gene to be present, fragmented, or missing if this approach revealed full-length homologous sequences (every exon in order on same contig), partial homologous sequences (only some exons or exons on two different contigs), or no homologous sequences, respectively.

Search for new genes in *An. gambiae* genome Our search for new *IRs* in AaegL5 involved an iterative process that resulted in the discovery of ~60 new *IR* genes in *An. gambiae*. We also used new *GR* genes in AaegL5 to uncover 4 new *GRs* in *An. gambiae*. These models were built in the Apollo instance at VectorBase and will be available in future updates to the *An. gambiae* geneset. Putative phylogenetic relationships and protein sequences for these new *An. gambiae* genes can be found in Extended Data Fig. 4-6 and Supplementary Data 20.

'Corrected' and 'fixed' genes A substantial minority of genes in the AaegL5 assembly contained loss-of-function (LOF) mutations that we inferred to be the result of either sequence/assembly errors or segregating polymorphism within the genome reference strain. In these cases, we chose to incorporate the intact alleles into our annotation set and analyses and refer to the genes as 'corrected' (minor updates to in-frame stop codons or small indels) or 'fixed' (major updates such as removal of large insertions of repetitive DNA or addition of a missing N-terminus). Sequence/assembly errors were inferred when both (1) LOF mutations occurred in regions where alignments of short-read Illumina data from the reference strain to AaegL5 were unusually spotty or showed sudden drops in coverage, and (2) we were able to find intact transcripts in a *de novo* transcriptome[28]. The short-read Illumina data were included as a supporting track in the Apollo instance used for annotation. Simple polymorphic LOF mutations such as in-frame stop codons and small frameshifting indels were also obvious in the aligned short-read Illumina data. Large polymorphic insertions of repetitive DNA were harder to detect, but were also 'fixed' when we were able to find intact alleles in either the NCBI TSA database or the previous AaegL3 genome assembly. Details on the types of LOF mutations corrected and the source of the intact sequences can be found in Supplementary Data 17.

Chemosensory receptor gene naming We chose to retain previously published names for all *ORs* and *GRs*, simply dropping one of the two pre-existing names for gene pairs that were merged into a single locus in AaegL5, and starting the numbering for new genes where the previous genesets left off. The only exceptions were a handful of *GR* isoforms that were renamed to maintain the standard of increasing lower case letter suffixes for sequentially ordered sets of private exons while accommodating new isoforms. The result for the *OR* and *GR* families is a set of non-sequential gene numbers with limited phylogenetic meaning but increased stability – a priority given the large number of previous publications on *OR* genes in particular. In contrast to the *ORs* and *GRs*, however, we chose to rename the majority of *IR* genes in *Ae. aegypti*. We made this decision because our annotation efforts doubled the size of the family for this mosquito and produced what we expect to be a nearly exhaustive compilation. We used the following set of four rules for renaming *IR* genes. The first two rules maintain pre-existing names, while the last two result in substantial changes.

(1) We retained *D. melanogaster* names for highly conserved *IRs* with clear 1-to-1 orthology across insects. These include *AaegIr8a, AaegIr21a, AaegIr25a, AaegIr40a, AaegIr60a, AaegIr68a, AaegIr76b,* and *AaegIr93a*.

(2) We retained *D. melanogaster* names for relatively conserved *IRs* with clear 1-to-2 orthology in *Ae. aegypti*, adding a '1' or '2' suffix for the two genes in *Ae. aegypti*. These include *Ir87a* (*AaegIr87a1* and *AaegIr87a2*) and *Ir31a* (*AaegIr31a1* and *AaegIr31a2*). We note that *DmelIr87a* does not cluster with its mosquito orthologues because it does not align well in the N-terminal

half. Nevertheless, orthology is supported by the fact that this gene is microsyntenic with neighbouring genes between flies and mosquitoes.
(3) We retained *D. melanogaster* roots for *IR* clades that are clearly related to specific *D. melanogaster* genes but have undergone more extensive species-specific expansion. These include genes in the *DmelIr75a-d* clade, the *DmelIr7a-g* clade, the *DmelIr41a* clade, and the *Dmel100a* clade. The corresponding members of each clade in *Ae. aegypti* were given the *D. melanogaster* number root with a single lower-case letter suffix (i.e. *AaegIr75a-l*, *AaegIr7a-r*, *AaegIr41a-q*, and *AaegIr100a-d*). Note that the specific suffix given in *Ae. aegypti* does not imply orthology with the *D. melanogaster* gene of the same suffix.
(4) We renamed all remaining genes in *Ae. aegypti* starting with *AaegIr101* and increasing by single integers up to *AaegIr172*. The vast majority of these genes (all but *AaegIr101-AaegIr104*) fall into massive species-specific expansions loosely related to taste receptors in the *DmelIr20a* clade[41]. Only 25 of these 72 genes had been previously identified and all had names in the range of Ir101 to Ir120). We similarly added many new *IR* genes to the previously described *An. gambiae* genesets[31,38], and renamed the entire family in that species according to the same rules. Old names for all *Ae. aegypti* and *An. gambiae IRs* are in Supplementary Data 17 and in parentheses on the ID line of the sequence fasta file (Supplementary Data 20).

Tree building The *Ae. aegypti* chemoreceptors in each family were aligned with those from *An. gambiae*[38,42] (incorporating updates to *Agam IR* and *GR* families from the current work) and *D. melanogaster*[39,40]using ClustalX v2[43] Chemoreceptors annotated from another Culicine mosquito with a publicly-available genome sequence, *Cx. quinquefasciatus*[44], have multiple near identical sequences that in light of our experience with *Ae. aegypti* are almost certainly the result of misassembly of alternative haplotypes. We therefore chose not to include receptors from that species in the trees, though we note that although no geneset annotation currently exists, CpipJ3[4] has de-duplicated and scaffolded the existing *Cx. quinquefasciatus* genome assembly onto chromosomes, and will likely be useful in resolving the chemoreceptor gene families in *Cx. quinquefasciatus*. For *ORs* and *GRs*, poorly aligned regions were trimmed using TrimAl v1.4[45] with the "gappyout" option that removes most poorly aligned or represented sequences. The *IR* family contains proteins that vary in the length and sequence of their N-terminal regions, so for this family the "strict" option was employed in TrimAl, which removed much of their N-terminal alignment. Maximum likelihood phylogenetic analysis was conducted using PhyML v3.0[46] with default parameters. In Extended Data Fig. 4-6, support levels for nodes are indicated by the size of black circles, reflecting approximate Log Ratio Tests (aLRT values ranging from 0-1 from PhyML v3.0 run with default parameters). Trees were arranged and coloured with FigTree v1.4.2 (http://tree.bio.ed.ac.uk/software/figtree/). We note that subsequent analysis revealed small changes to gene models for *AaegOr34*, *AaegOr37*, *AaegOr82*, and *AaegOr97*, which are not reflected in this tree.

Expression analyses We reanalysed published RNA-seq data[28] to quantify chemoreceptor expression in neural tissues using the new geneset (for details of alignment see methods section entitled "Alignment of RNA-seq data to AaegL5 and quantification of gene expression"). We converted our Apollo-generated GFF3 file into the GTF format and provided this GTF file as input to featureCounts[47]. We counted reads across coding regions only since RNA-seq evidence for UTRs was inconsistent across genes and some UTRs appeared to contain repetitive sequences that introduced mapping artefacts into inferred expression levels. We excluded UTRs by specifying the CDS flag (-t CDS) for each gene (-g gene_id) with an intact open-reading frame. We did not annotate UTRs for pseudogenes and were therefore able to count reads across

all exons (-t exon) for genes whose coding regions were disrupted by loss-of-function mutations. We pooled reads across replicate libraries derived from the same tissue and time point and used the previously computed normalization factors to calculate TPM-normalized expression levels for each chemoreceptor in each tissue. For visualization, we log10-transformed TPM expression levels using a pseudocount of 1. Expression values (clustered by the R function 'hclust') are presented in Extended Data Fig. 7 (more detailed versions of each gene family with gene names are available at https://github.com/VosshallLab/AGWG-AaegL5). We note that subsequent analysis revealed small changes to gene models for *AaegOr34*, *AaegOr37*, *AaegOr82*, and *AaegOr97*, which are not reflected in this expression analysis.

Chemosensory receptor overview We identified a total of 117 odorant receptors (*ORs*), 72 gustatory receptors (*GRs*; encoding 107 transcripts), and 135 ionotropic receptors (*IRs*) in the AaegL5 assembly. Our new genesets include all previously recognized genes within each family[29-31]. However, we found that 20-30% of previously recognized receptors comprised closely-related pairs that were merged into a single locus in AaegL5 (Fig. 2b and Supplementary Data 17). These were presumably found in regions of the AaegL3 assembly where divergent haplotypes were erroneously represented on separate contigs. We note that the AaegL4 assembly[4], although not annotated, would likely have resolved many of the same issues. Previous experimental work showed that one pair of similar AaegL3.4 genes (*AaegOr4*, *AaegOr5)* do indeed segregate at a single locus in *Ae. aegypti*[48].

As described in the main text, we also identified new (e.g. previously unannotated) receptor genes, including 2 new *ORs*, 2 new *GRs*, 8 new isoforms of previously identified *GRs*, and a surprising 54 new *IRs* (Supplementary Data 17). Six of the new genes and 4 of the new isoforms (private exons only) were missing or fragmented in AaegL3 (Supplementary Data 17). The rest were present but not recognized. The large number of new *IRs* nearly doubled the size of the family in *Ae. aegypti* and led to the discovery of a similarly sized group of 64 new *IRs* in the African malaria mosquito *An. gambiae*. As described above, we therefore decided to revise the naming scheme for *IRs* in both mosquito species. In contrast, we left *OR* and *GR* names intact (with the exception of a handful of *GR* isoforms), dropping names for merged genes, and beginning the numbering for new genes where the old genesets left off. Old names for all genes and transcripts are listed in (Supplementary Data 17).

One of the two new *OR* genes, *AaegOr133*, has a 1-to-1 orthologue in the malaria mosquito *An. gambiae* (*AgamOr80*) and two relatively close paralogues in *D. melanogaster* (Extended Data Fig. 4), and is one of the most highly expressed *ORs* on female, and particularly male, antennae (Extended Data Fig. 7). Several of the new *IR* genes also fall in clades with clear relatives in *D. melanogaster* (Extended Data Fig. 6), and these tend to be expressed in antenna (*AaegIr75l* and *AaegIr31a2*) or proboscis (*AaegIr7p, AaegIr7q, AaegIr7r*) or both (*AaegIr41m*) (Extended Data Fig. 7). However, the majority of new *IRs* in both mosquito species fall into a large mosquito specific clade loosely related to the *IR20a* clade of taste receptors in *Drosophila*[41] (Extended Data Fig. 6). *Ae. aegypti* members of this clade tend to be expressed in adult forelegs and midlegs of both sexes, or females only, suggesting a role in contact chemosensation (Extended Data Fig. 7).

In addition to adding new genes, we also updated the models of many previously recognized genes. Notably, we extended or added exons to 60% of all previously recognized *IRs* (49 of 81 genes, Supplementary Data 17), resulting in an average protein length increase of over 200 amino acids and greatly narrowing the length distribution for the *IR* family as a whole (data not shown). We also completed the models for 5 *OR* genes that were designated 'partial' in

AaegL3.4 (ref. [29]), made major changes to the N-terminus of 8 *GR* genes and 1 *GR* isoform, and made minor changes to the start sites and splice junctions of numerous genes in all three families (Supplementary Data 17). These changes were made manually based on extensive RNA-seq data[28] and careful search of flanking sequences for homology to other receptors.

The AaegL5.0 sequences for 62 of a total of 359 receptor transcripts in our new annotation set include loss-of-function mutations that should render them pseudogenes. However, we infer that 20 of these cases likely reflect within-species polymorphism and another 9 result from sequencing/assembly errors. We chose to include updated, intact alleles for these receptors in our genesets (Supplementary Data 17-20) and refer to these sequences as either 'corrected' (minor difference between AaegL5 and updated allele) or 'fixed' (major difference between AaegL5 and updated allele) ('C' and 'F' suffixes in Extended Data Fig. 4-6 and Supplementary Data 17). After accounting for these updates, we are left with 33 receptor transcripts that we consider pseudogenes – 10 of 117 *ORs*, 12 of 107 *GRs*, and 11 of 135 *IRs* ('P' suffixes in Extended Data Fig. 4-6 and Supplementary Data 17).

The new assembly allowed us to describe the distribution of chemoreceptors across the three chromosomes of *Ae. aegypti* (Fig. 2a and Extended Data Fig. 3). We also inferred new phylogenetic trees for receptors in each family (Extended Data Fig. 4-6) and revised expression estimates for various neural tissues of adult males and females using previously published ultra-deep RNAseq data[28] (Extended Data Fig. 7; see https://github.com/VosshallLab/AGWG-AaegL5 for versions with gene names for each of the three chemosensory receptor families). We hope these analyses will serve as a resource for the community.

Large increase of *AaegIRs* and reannotation of *AgamIRs* In light of our recognition of many new *IR* genes in *Ae. aegypti*, we re-examined the *An. gambiae* genome and discovered 64 new *AgamIR* genes to add to the 46 previously described *AgamIRs*[31,38], bringing the total to 110. Because 6 of these are pseudogenic, the functional *IR* repertoire in *An. gambiae* appears to be 104 proteins. Some of these new genes are related to conserved *IRs* in *D. melanogaster*, for example, *AgamIr87a* (an intronless gene on a 52 kb scaffold in the original PEST strain assembly that was not included in the PEST chromosomal assembly). *AgamIr31a* has a divergent relative immediately downstream of it in chromosome 3R, so the original was renamed *AgamIr31a1*, and the newly recognized gene *AgamIr31a2*. But the vast majority of the new genes, as with *Ae. aegypti*, are related to the genes originally named *AgamIr133-139*, *AgamIr140.1/2*, and *AgamIr142*. In our tree (Extended Data Fig. 6) these and the large number of new *Ae. aegypti IRs* are confidently related to the clade of divergent *IRs* in *D. melanogaster* that have been demonstrated to be candidate gustatory receptors and called the *Ir20a* clade (apparently for the lowest numbered *IR* in the clade)[41,49]. Like the *Ir7* clade, which are also candidate gustatory receptors in *D. melanogaster*[31], this clade appears to have expanded independently in *D. melanogaster* and mosquitoes. Even comparing these two mosquitoes, multiple expansions of sublineages of the clade have occurred in the anopheline versus culicine lineages, suggesting that gene duplicates have been retained to perceive different chemicals relevant to the chemical ecology of each species. It is noteworthy that all six pseudogenic *AgamIRs*, 9 of the 10 pseudogenic *AaegIRs*, and all 4 of the pseudogenic *DmelIRs* belong to this rapidly-evolving clade, supporting the idea that this clade has undergone rapid gene family evolution, with some receptors being lost to pseudogenization or lost from each genome completely, as, for example *Ae. aegypti* has lost the relative of *AgamIr105*.

Previously recognized *GR* genes Most of the 114 *GRs* previously described[30] were present in the new assembly, however 16 *AaegGR* protein names have been dropped as they

were nearly identical duplicates of other genes and are not present in the new genome assembly (*AaegGr12P*, *AaegGr24P*, *AaegGr28*, *AaegGr38P*, *AaegGr40a-h*, *AaegGr51*, *AaegGr52P*, *AaegGr70*, and *AaegGr71*). They were all on separately assembled scaffolds, presumably assemblies of alternative haplotypes. The departure of these models disrupts the naming conventions employed earlier[30]. Furthermore, now that the arrangement of these genes on the chromosomes is known, the names are often "chromosomally" and "phylogenetically" jumbled. Nevertheless, this is a problem shared with many arthropod draft genomes, e.g. *An. gambiae*[37], and even the *D. melanogaster* chemoreceptors, which were named for their cytological locations and hence have some "chromosomal" rationale, are "phylogenetically" jumbled[39]. The original *AaegGR* names have been employed in studies of phylogenetic comparison[44] and expression[28,50,51]. We therefore chose to retain the original gene numbers, dropping the departed duplicates with higher number and not replacing them, and adding newly recognized genes with the next number in order.

Two new *AaegGRs* Two previously unrecognized divergent *AaegGR* genes were discovered. *AaegGr80* was discovered as an apparently co-transcribed gene with *AaegGr72* (there are just 98 bp between the stop codon of *AaegGr72* and the start codon of *AaegGr80*). This locus was previously modelled in NCBI as LOC110680332. The ancestral final short exon of *GR* genes contains a conserved TYhhhhhQF motif, where h is any hydrophobic amino acid, except in the sugar receptors where the motif is TYEhhhhQF[52], precisely six codons after a nearly universally present phase-0 intron[53,54]. TBLASTN searches of the genome seeking additional new *GRs* were therefore performed using the amino acids encoded by this final exon from representative *GRs*, with LQ before them to represent the consensus bases of a phase-0 intron 3' acceptor site (TTGCAG). To increase sensitivity for these searches the default parameters were modified, raising the Expect Threshold from 10 to 1000, reducing the Word Size from 6 to 2, and removing the Low Complexity Filter. These searches revealed one more new gene, *AaegGr81*, discovered with *AaegGr80* as query.

Four new *AgamGRs* There is an unannotated relative of *AaegGr81* in the *An. gambiae* genome, on chromosome 2R from 457,227-458,928 bp, which is a neighbour of a cluster of annotated *GRs*, including *AgamGr58-60*, and so was named *AgamGr61*, the next available number. *Cx. quinquefasciatus* also has a previously unrecognized relative of this gene, here named *CquiGr78*. *An. gambiae* also has an unannotated relative of *AaegGr80*, on chromosome 2R from 54,382,599-54,383,860 bp and immediately downstream of *AgamGr54*, which we name *AgamGr62*, but *Cx. quinquefasciatus* has apparently lost this gene. Furthermore, an unannotated relative of the highly divergent *AaegGr79* was recognized in the *An. gambiae* genome, on chromosome 3R from 44,045,062-44,046,334 and named *AgamGr63* (*Cx. quinquefasciatus* again has no orthologue). It has no *GR* neighbours and is partially modelled as AGAP028572. Finally, a fourth previously unrecognized *An. gambiae GR* was communicated by Xiaofan Zhou (personal communication), having been discovered (along with independent discovery of *AgamGr61-63*) as part of the 16 *Anopheles* species genome project[42] and is named *AgamGr64*. It is located on chromosome 3R from 43,704,508-43,705,788bp and is near the *AgamGr9-12* genes (the culicines have no orthologue). These four new *An. gambiae GR* gene models have been communicated to VectorBase to be incorporated in future *An. gambiae* genesets.

Cleaning-up and renumbering alternate *GR* isoforms An additional complication to this improvement of the chemoreceptor gene models in the new genome assembly arises in the *GR* family, which has eight alternatively-spliced loci, a phenomenon recognized with the description of the family in *D. melanogaster*[39,53]and present in many other insects including *An. gambiae*[37]

and *Cx. quinquefasciatus*[44]. These isoforms consist of one or more exons encoding the N-terminus of a *GR* spliced to a single set of exons encoding the C-terminus, and the deep RNA-seq coverage[28] provides support for most of them. Unfortunately, some of these alternatively-spliced exons were separately assembled in the original assembly, and hence were not associated with the relevant locus, while the large and near identical *AaegGr39/40* loci were duplicates that are now resolved into one locus with eight isoforms. These and other issues require renumbering of the isoforms for several such loci.

<u>Updated *GR* gene models</u> As described above, *AaegGr80* and *Aaeg81* have been added to the family. Three genes (*AaegGr48*, *AaegGr50*, and *AaegGr75*) are now intact in the new assembly, versus being pseudogenes in the original, and one gene model (*AaegGr45*) is newly recognized as a pseudogene (an intron interrupting the first exon was previously incorrectly modelled to remove a stop codon). Another five gene models were improved (*AaegGr4*, *AaegGr5*, *AaegGr7*, *AaegGr35*, and *AaegGr37*), largely in light of the transcript and RNA-seq alignments, while the assembly provided two exons that were missing from *AaegGr30* (although those were built from raw genome reads previously). In addition, several proteins resulting from alternative splicing of loci have been modified or added. The *AaegGr39a-h* and *AaegGr40a-h* loci were near identical in the original assembly, but differed in having different isoforms pseudogenized. The new assembly has only one version of this locus, which retains the *AaegGr39a-h* name, with three intact isoforms (a, c, and e) and five pseudogenic ones (b, d, f-h). Finally, *Ae. aegypti* has a complicated set of alternatively-spliced genes (*AaegGr20a-m*, *AaegGr60a-d*, *AaegGr 61a-c*, and *AaegGr62*) related to the alternatively-spliced *AgamGr37a-f* gene. *AaegGr60-62* were single isoform genes in the original annotation, and while *AaegGr62* remains that way, three additional isoforms are now recognized for *AaegGr60a-d* and two more for *AaegGr61a-c*. Furthermore, the neighbouring *AaegGr20* locus has acquired two more isoforms for a total of 13 (it had isoforms a-k and now has isoforms a-m with the identification of a new isoform after h, now called i, that was so divergent it was not recognized previously, and an ignored pseudogenic fragment before k that is now intact and named l - the other isoforms are renamed to accommodate these).

<u>Fixed/corrected *GR* genes</u> An additional complication is that for six genes the new assembly does not accurately reflect the genome, as indicated both by comparison with the original assembly, and with a lane of Illumina reads from a single individual and/or available transcript sequences in the TSA[28]. One of these is a base change of an intron 3' acceptor site from CAG to CAT (*AaegGr17*), and four are single-base frameshifting indels in homopolymers in exons (*AaegGr53*, *AaegGr55*, *AaegGr66*, and *AaegGr72*) (the single individual is heterozygous for most of these mutations). Instead of treating these genes as pseudogenes, their sequences were corrected to encode an intact protein. Another problem is presented by *AaegGr25*, which is intact in the original assembly and the *de novo* transcriptome, but has suffered an insertion of a 500 bp repeat present widely in the genome, so the intact version is employed herein. A particularly difficult situation is presented by *AaegGr63*, for which the new assembly is seriously compromised by numerous single-base indels (presumably because it is covered by one or very few Pacific Biosciences reads). This gene was therefore modelled based on the original genome assembly. The available transcripts for this gene and its head-to-head neighbour, *AaegGr64*, also suggest models that have major length differences from all other *GRs*, so again the original gene models and proteins were employed for them. Finally, while some genes in the new assembly have identical sequences to the old assembly, others have up to

several percentage sequence difference, and with the exceptions noted above, the new gene sequences were employed.

Summary of *GR* analysis The final result is that we annotate a total of 72 genes potentially encoding 107 proteins through alternative splicing of 8 loci, but 12 of these are pseudogenic, leaving 95 apparently functional *GRs*. The *An. gambiae GRs* total 93 proteins from 64 genes, none of which are obviously pseudogenic. All of our *AaegGR* proteins, as well as the four new *AgamGRs* and *CquiGr78*, are provided in fasta format in Supplementary Data 19-20.

Relationships of *AaegGRs* to *DmelGRs* and *AgamGRs* including biological roles Phylogenetic analysis of these *AaegGRs* along with those of *An. gambiae* and *D. melanogaster* reveals diverse aspects of the evolution of this gene family in these mosquitoes (Extended Data Fig. 5). While the three carbon dioxide receptors are highly conserved single orthologues in each mosquito[55,56], there has been considerable evolution of the sugar receptors[52], including pseudogenization of two genes in *Ae. aegypti* (*AaegGr8P* and *AaegGr13P*) and loss of a gene lineage from *An. gambiae*. Four other clades of mosquito *GRs* have clear relatives in *D. melanogaster* that likely inform their biological roles. First, *AaegGr34* along with *AgamGr25* are highly conserved orthologues of *DmelGr43a*, a fructose receptor expressed in both peripheral gustatory neurons and within the brain[57]. Second, *AaegGr19a-c* is an alternatively-spliced locus encoding three quite similar proteins with single orthologues in *An. gambiae* (*AgamGr33*) and this lineage is related to *DmelGr28a* and the alternatively-spliced *DmelGr28bA-E*, genes that also have unusual expression patterns beyond peripheral gustatory neurons[58], and *DmelGr28bD* is involved in temperature sensing in flies[59]. Third, *AaegGr37* and the alternatively-spliced *AaegGr39a-h* locus, along with *AgamGr9a-n*, *AgamGr10-AgamGr12*, and *AgamGr64*, are related to *DmelGr32*, *DmelGr68*, and *DmelGr39aA-D*, proteins implicated in contact pheromone perception in flies and regulation of mating and aggression[60-62]. The complex evolution of these often alternatively-spliced loci mirrors that of the *DmelGr39a* locus within the *Drosophila* genus[63]. Another *D. melanogaster GR* implicated in mating behaviour, *DmelGr33a*[64,65] has a convincing *An. gambiae* relative in *AgamGr43*, but has been lost from the culicines. Fourth, *AaegGr14* and *AgamGr2* are highly conserved orthologues of *DmelGr66a*, a well-known bitter taste receptor[66]. Most of the remaining *DmelGRs* are implicated in perception of bitter tastants[67-69], and the same is likely true of many of the remaining mosquito *GRs*, some of which have complicated relationships with *DmelGR* lineages, for example, these mosquitoes each have three *GRs* (*AaegGr16-18, AGamGr3/4* and *AgamGr7*) that cluster with *DmelGr8a*, which participates in perception of a plant-derived insecticide, L-canavanine[66]. Surprisingly, *Ae. aegypti* and *Cx. quinquefasciatus* have lost the orthologue of *AgamGr43*, which is apparently related to *DmelGr33a* (but does not share microsynteny with it), a well-known bitter taste receptor that is also involved in courtship behaviour[64,65].

The remaining relationships of these mosquito *GRs* are typical of insect chemoreceptors (Extended Data Fig. 5), ranging from: 1) highly conserved single orthologues comparable to the carbon dioxide or fructose receptors (e.g. *AaegGr73*/*AgamGr53* or *AaegGr30*/*AgamGr47*, whose ligands and biological roles are likely to be shared across these mosquitoes but which were apparently lost from drosophilids), to 2) instances of loss from one or more lineages (e.g. the *Ae. aegypti* orthologues of *AgamGr1*, *AgamGr34*, *AgamGr58*, *AgamGr59*, and *AgamGr60* were lost), to 3) major gene-lineage-specific expansions in each species. The three most prominent of the latter are the independent expansions of *AgamGr55*/*AaegGr74a-e* and *AgamGr56a-f*/*AaegGr67a-e*/ *AaegGr68*/ *AaegGr69*, an expansion of 15 *AegGRs* related to *AgamGr40*, and the clade that includes *AaegGr20a-m*, *AaegGr60a-d*, *AaegGr61a-c*, and *AaegGr62*, all of which

are neighbours in chromosome 3, and related to *AgamGr37a-f*. This last expansion of 6 proteins in *An. gambiae* to 21 in *Ae. aegypti*, and an even larger number in *Cx. quinquefasciatus*[44], likely indicates an important involvement in idiosyncratic aspects of the chemical ecology of culicine mosquitoes.

Curation of ligand-gated ion channels and larvacidal activity of agricultural and veterinary insecticides

Putative *Ae. aegypti* cys-loop ligand-gated ion channel subunits were initially identified by searching the 2007 *Ae. aegypti* AaegL3 genome with TBLASTn[70] using protein sequences of every member of the *D. melanogaster* cys-loop ligand-gated ion channel superfamily. In many cases the subunit coding sequences were incomplete due to regions showing low levels of homology, in particular the N-terminal signal peptide sequence and the hyper-variable intracellular region between the third and fourth transmembrane domains. These subunit sequences were used to search the latest AaegL5 RefSeq annotation through BLAST analysis, which in many cases completed missing sequence information (Supplementary Data 24). The neighbour-joining method [71] and bootstrap resampling, implemented in ClustalX[43], was used to construct a phylogenetic tree which was then viewed using TreeView[72] (Extended Data Fig. 10d).

To measure the effect of insecticides on *Ae. aegypti* larval behaviour, 3-10 larvae were dispensed manually into each well of a 96-well plate. Insecticides (imidacloprid, triflumezopyrim (targeting nAChRs[73]), abamectin (targeting primarily GluCls[74,75]) and fipronil (targeting primarily GABARs[74,75]) were added at a range of concentrations from 10^{-11} to 10^{-4} M. Larvae were incubated in compounds for 4 hr. The plate was then transferred to a video monitoring system (Extended Data Fig. 10c) which consisted of an LED light source backlighting the 96-well plate and an Andor Neo camera and Pentax YF3528 lens. Images of the whole plate were acquired using a MATLAB script. The normalised movement index is plotted against the concentration of each compound. The movement index was derived by calculating the variance of a movie of each well in a 96-well plate and counting the number of pixels whose variance exceeds the mean variance by more than 4 standard deviations. Motility was estimated using the following algorithm: 1) a pair of images was acquired, separated by 10 ms. 2) the first image is subtracted from the second image to obtain a difference image. 3) pixels in the difference image with a value less than zero are set to zero. 4) pixels whose value is greater than 3 standard deviations above the mean of the image are set to 1, the rest are set to 0. 5) The mean of the pixels in each well is calculated to give a movement index. 6) The movement indices for the entire plate were divided by the maximum value to yield a normalized movement index. Ying Song of DowDuPont Inc. provided triflumezopyrim for the experiments in Extended Data Fig. 10c.

References cited

1 R Core Team. R: A language and environment for statistical computing. *R Foundation for Statistical Computing, Vienna, Austria.* https://www.r-project.org/ (2017).

2 Chin, C. S. *et al.* Nonhybrid, finished microbial genome assemblies from long-read SMRT sequencing data. *Nat Methods* **10**, 563-569 (2013).

3 Chin, C. S. *et al.* Phased diploid genome assembly with single-molecule real-time sequencing. *Nat Methods* **13**, 1050-1054 (2016).

4 Dudchenko, O. *et al. De novo* assembly of the *Aedes aegypti* genome using Hi-C yields chromosome-length scaffolds. *Science* **356**, 92-95 (2017).
5 Dudchenko, O. *et al.* The Juicebox Assembly Tools module facilitates de novo assembly of mammalian genomes with chromosome-length scaffolds for under $1000. *bioRxiv*, https://www.biorxiv.org/content/early/2018/01/28/254797 (2018).
6 Robinson, J. T. *et al.* Juicebox.js Provides a Cloud-Based Visualization System for Hi-C Data. *Cell Syst* **6**, 256-258.e251 (2018).
7 Durand, N. C. *et al.* Juicer provides a one-click system for analyzing loop-resolution Hi-C experiments. *Cell Syst* **3**, 95-98 (2016).
8 Harris, R. S. *Improved pairwise alignment of genomic DNA*, Ph.D. Thesis, The Pennsylvania State University, (2007).
9 Waterhouse, R. M. et al. BUSCO applications from quality assessments to gene prediction and phylogenomics. *Mol Biol Evol* **35**, 543–548 (2017).
10 Nene, V. *et al.* Genome sequence of *Aedes aegypti*, a major arbovirus vector. *Science* **316**, 1718-1723 (2007).
11 Takahashi, H., Kato, S., Murata, M. & Carninci, P. CAGE (cap analysis of gene expression): a protocol for the detection of promoter and transcriptional networks. *Methods Mol Biol* **786**, 181-200 (2012).
12 Leenen, F. A. *et al.* Where does transcription start? 5'-RACE adapted to next-generation sequencing. *Nucleic Acids Res* **44**, 2628-2645 (2016).
13 Smit, A. F. A., Hubley, R. & Green, P. RepeatMasker Open-4.0 http://www.repeatmasker.org/ (2013-2015).
14 Van der Auwera, G. A. *et al.* From FastQ data to high confidence variant calls: the Genome Analysis Toolkit best practices pipeline. *Curr Protoc Bioinformatics* **43**, 11.10.11-33 (2013).
15 Altschul, S. F. *et al.* Protein database searches using compositionally adjusted substitution matrices. *FEBS J* **272**, 5101-5109 (2005).
16 Gramates, L. S. *et al.* FlyBase at 25: looking to the future. *Nucleic Acids Res* **45**, D663-d671 (2017).
17 Giraldo-Calderon, G. I. *et al.* VectorBase: an updated bioinformatics resource for invertebrate vectors and other organisms related with human diseases. *Nucleic Acids Res* **43**, D707-713 (2015).
18 Smith, A. F., Posakony, J. W. & Rebeiz, M. Automated tools for comparative sequence analysis of genic regions using the GenePalette application. *Dev Biol* **429**, 158-164 (2017).
19 Biessmann, H., Donath, J. & Walter, M. F. Molecular characterization of the *Anopheles gambiae* 2L telomeric region via an integrated transgene. *Insect Mol Biol* **5**, 11-20 (1996).
20 Sievers, F. *et al.* Fast, scalable generation of high-quality protein multiple sequence alignments using Clustal Omega. *Mol Syst Biol* **7**, 539 (2011).
21 Merabet, S. & Mann, R. S. To be specific or not: The critical relationship between Hox and TALE proteins. *Trends Genet* **32**, 334-347 (2016).
22 Chen, A., Holmes, S. P. & Pietrantonio, P. V. Molecular cloning and functional expression of a serotonin receptor from the Southern cattle tick, *Boophilus microplus* (Acari: Ixodidae). *Insect Mol Biol* **13**, 45-54 (2004).

23 Goujon, M. *et al.* A new bioinformatics analysis tools framework at EMBL-EBI. *Nucleic Acids Res* **38**, W695-699 (2010).

24 Meyer, J. M., Ejendal, K. F., Watts, V. J. & Hill, C. A. Molecular and pharmacological characterization of two D(1)-like dopamine receptors in the Lyme disease vector, *Ixodes scapularis*. *Insect Biochem Mol Biol* **41**, 563-571 (2011).

25 Meyer, J. M. *et al.* A "genome-to-lead" approach for insecticide discovery: pharmacological characterization and screening of *Aedes aegypti* D(1)-like dopamine receptors. *PLoS Negl Trop Dis* **6**, e1478 (2012).

26 Lee, E. *et al.* Web Apollo: a web-based genomic annotation editing platform. *Genome Biol* **14**, R93 (2013).

27 Giraldo-Calderon, G. I., Zanis, M. J. & Hill, C. A. Retention of duplicated long-wavelength opsins in mosquito lineages by positive selection and differential expression. *BMC Evol Biol* **17**, 84 (2017).

28 Matthews, B. J., McBride, C. S., DeGennaro, M., Despo, O. & Vosshall, L. B. The neurotranscriptome of the *Aedes aegypti* mosquito. *BMC Genomics* **17**, 32 (2016).

29 Bohbot, J. *et al.* Molecular characterization of the *Aedes aegypti* odorant receptor gene family. *Insect Mol Biol* **16**, 525-537 (2007).

30 Kent, L. B., Walden, K. K. & Robertson, H. M. The *Gr* family of candidate gustatory and olfactory receptors in the yellow-fever mosquito *Aedes aegypti*. *Chem Senses* **33**, 79-93 (2008).

31 Croset, V. *et al.* Ancient protostome origin of chemosensory ionotropic glutamate receptors and the evolution of insect taste and olfaction. *PLoS Genet* **6**, e1001064 (2010).

32 Altschul, S. F. *et al.* Gapped BLAST and PSI-BLAST: a new generation of protein database search programs. *Nucleic Acids Res* **25**, 3389-3402 (1997).

33 Morgulis, A. *et al.* Database indexing for production MegaBLAST searches. *Bioinformatics* **24**, 1757-1764 (2008).

34 Wu, T. D. & Watanabe, C. K. GMAP: a genomic mapping and alignment program for mRNA and EST sequences. *Bioinformatics* **21**, 1859-1875 (2005).

35 Birney, E., Clamp, M. & Durbin, R. GeneWise and Genomewise. *Genome Res* **14**, 988-995 (2004).

36 Quinlan, A. R. & Hall, I. M. BEDTools: a flexible suite of utilities for comparing genomic features. *Bioinformatics* **26**, 841-842 (2010).

37 Hill, C. A. *et al.* G protein-coupled receptors in *Anopheles gambiae*. *Science* **298**, 176-178 (2002).

38 Liu, C. *et al.* Distinct olfactory signaling mechanisms in the malaria vector mosquito *Anopheles gambiae*. *PLoS Biol* **8** (2010).

39 Robertson, H. M., Warr, C. G. & Carlson, J. R. Molecular evolution of the insect chemoreceptor gene superfamily in *Drosophila melanogaster*. *Proc Natl Acad Sci U S A* **100 Suppl 2**, 14537-14542 (2003).

40 Benton, R., Vannice, K. S., Gomez-Diaz, C. & Vosshall, L. B. Variant ionotropic glutamate receptors as chemosensory receptors in *Drosophila*. *Cell* **136**, 149-162 (2009).

41 Koh, T. W. *et al.* The *Drosophila IR20a* clade of ionotropic receptors are candidate taste and pheromone receptors. *Neuron* **83**, 850-865 (2014).

42 Neafsey, D. E. *et al.* Mosquito genomics. Highly evolvable malaria vectors: the genomes of 16 *Anopheles* mosquitoes. *Science* **347**, 1258522 (2015).

43 Larkin, M. A. *et al.* Clustal W and Clustal X version 2.0. *Bioinformatics* **23**, 2947-2948 (2007).
44 Arensburger, P. *et al.* Sequencing of *Culex quinquefasciatus* establishes a platform for mosquito comparative genomics. *Science* **330**, 86-88 (2010).
45 Capella-Gutierrez, S., Silla-Martinez, J. M. & Gabaldon, T. trimAl: a tool for automated alignment trimming in large-scale phylogenetic analyses. *Bioinformatics* **25**, 1972-1973 (2009).
46 Guindon, S. *et al.* New algorithms and methods to estimate maximum-likelihood phylogenies: assessing the performance of PhyML 3.0. *Syst Biol* **59**, 307-321 (2010).
47 Liao, Y., Smyth, G. K. & Shi, W. featureCounts: an efficient general purpose program for assigning sequence reads to genomic features. *Bioinformatics* **30**, 923-930 (2014).
48 McBride, C. S. *et al.* Evolution of mosquito preference for humans linked to an odorant receptor. *Nature* **515**, 222-227 (2014).
49 Stewart, S., Koh, T. W., Ghosh, A. C. & Carlson, J. R. Candidate ionotropic taste receptors in the *Drosophila* larva. *Proc Natl Acad Sci U S A* **112**, 4195-4201 (2015).
50 Sparks, J. T., Vinyard, B. T. & Dickens, J. C. Gustatory receptor expression in the labella and tarsi of *Aedes aegypti*. *Insect Biochem Mol Biol* **43**, 1161-1171 (2013).
51 Sparks, J. T., Bohbot, J. D. & Dickens, J. C. The genetics of chemoreception in the labella and tarsi of *Aedes aegypti*. *Insect Biochem Mol Biol* **48**, 8-16 (2014).
52 Kent, L. B. & Robertson, H. M. Evolution of the sugar receptors in insects. *BMC Evol Biol* **9**, 41 (2009).
53 Clyne, P. J., Warr, C. G. & Carlson, J. R. Candidate taste receptors in *Drosophila*. *Science* **287**, 1830-1834 (2000).
54 Robertson, H. M. The insect chemoreceptor superfamily is ancient in animals. *Chem Senses* **40**, 609-614 (2015).
55 Jones, W. D., Cayirlioglu, P., Kadow, I. G. & Vosshall, L. B. Two chemosensory receptors together mediate carbon dioxide detection in *Drosophila*. *Nature* **445**, 86-90 (2007).
56 Robertson, H. M. & Kent, L. B. Evolution of the gene lineage encoding the carbon dioxide receptor in insects. *J Insect Sci* **9**, 19 (2009).
57 Miyamoto, T., Slone, J., Song, X. & Amrein, H. A fructose receptor functions as a nutrient sensor in the *Drosophila* brain. *Cell* **151**, 1113-1125 (2012).
58 Thorne, N. & Amrein, H. Atypical expression of *Drosophila* gustatory receptor genes in sensory and central neurons. *J Comp Neurol* **506**, 548-568 (2008).
59 Ni, L. *et al.* A gustatory receptor paralogue controls rapid warmth avoidance in *Drosophila*. *Nature* **500**, 580-584 (2013).
60 Bray, S. & Amrein, H. A putative *Drosophila* pheromone receptor expressed in male-specific taste neurons is required for efficient courtship. *Neuron* **39**, 1019-1029 (2003).
61 Miyamoto, T. & Amrein, H. Suppression of male courtship by a *Drosophila* pheromone receptor. *Nat Neurosci* (2008).
62 Koganezawa, M., Haba, D., Matsuo, T. & Yamamoto, D. The shaping of male courtship posture by lateralized gustatory inputs to male-specific interneurons. *Curr Biol* **20**, 1-8 (2010).
63 Gardiner, A., Barker, D., Butlin, R. K., Jordan, W. C. & Ritchie, M. G. Evolution of a complex locus: exon gain, loss and divergence at the *Gr39a* locus in *Drosophila*. *PLoS One* **3**, e1513 (2008).

64 Moon, S. J., Lee, Y., Jiao, Y. & Montell, C. A *Drosophila* gustatory receptor essential for aversive taste and inhibiting male-to-male courtship. *Curr Biol* **19**, 1623-1627 (2009).

65 Hu, Y. *et al. Gr33a* modulates *Drosophila* male courtship preference. *Sci Rep* **5**, 7777 (2015).

66 Shim, J. *et al.* The full repertoire of *Drosophila* gustatory receptors for detecting an aversive compound. *Nat Commun* **6**, 8867 (2015).

67 Thorne, N., Chromey, C., Bray, S. & Amrein, H. Taste perception and coding in *Drosophila*. *Curr Biol* **14**, 1065-1079 (2004).

68 Weiss, L. A., Dahanukar, A., Kwon, J. Y., Banerjee, D. & Carlson, J. R. The molecular and cellular basis of bitter taste in *Drosophila*. *Neuron* **69**, 258-272 (2011).

69 Delventhal, R. & Carlson, J. R. Bitter taste receptors confer diverse functions to neurons. *Elife* **5** (2016).

70 Altschul, S. F., Gish, W., Miller, W., Myers, E. W. & Lipman, D. J. Basic local alignment search tool. *J Mol Biol* **215**, 403-410 (1990).

71 Saitou, N. & Nei, M. The neighbor-joining method: a new method for reconstructing phylogenetic trees. *Mol Biol Evol* **4**, 406-425 (1987).

72 Page, R. D. TreeView: an application to display phylogenetic trees on personal computers. *Comput Appl Biosci* **12**, 357-358 (1996).

73 Ihara, M., Buckingham, S. D., Matsuda, K. & Sattelle, D. B. Modes of action, resistance and toxicity of insecticides targeting nicotinic acetylcholine receptors. *Curr Med Chem* **24**, 2925-2934 (2017).

74 Buckingham, S. D., Pym, L. & Sattelle, D. B. Oocytes as an expression system for studying receptor/channel targets of drugs and pesticides. *Methods Mol Biol* **322**, 331-345 (2006).

75 Wolstenholme, A. J. Glutamate-gated chloride channels. *J Biol Chem* **287**, 40232-40238 (2012).
