# Supplementary material for: Improved reference genome of Aedes aegypti informs arbovirus vector control
Source: Nature. 2018 Nov 14;563(7732):501–7. doi: 10.1038/s41586-018-0692-z (PMC6421076; doi:10.1038/s41586-018-0692-z)
Supplement: Supplementary file 2 — Reporting Summary [file 41586_2018_692_MOESM2_ESM.pdf]

nature research

Corresponding author(s): *Double-blind peer review submissions: write DBPR and your manuscript number here instead of author names.*

Reporting Summary

Nature Research wishes to improve the reproducibility of the work that we publish. This form provides structure for consistency and transparency in reporting. For further information on Nature Research policies, see Authors & Referees and the Editorial Policy Checklist.

Statistical parameters

When statistical analyses are reported, confirm that the following items are present in the relevant location (e.g. figure legend, table legend, main text, or Methods section).

n/a	Confirmed	
☐	☒	The exact sample size (*n*) for each experimental group/condition, given as a discrete number and unit of measurement
☐	☒	An indication of whether measurements were taken from distinct samples or whether the same sample was measured repeatedly
☐	☒	The statistical test(s) used AND whether they are one- or two-sided<br>*Only common tests should be described solely by name; describe more complex techniques in the Methods section.*
☐	☒	A description of all covariates tested
☐	☒	A description of any assumptions or corrections, such as tests of normality and adjustment for multiple comparisons
☐	☒	A full description of the statistics including central tendency (e.g. means) or other basic estimates (e.g. regression coefficient) AND variation (e.g. standard deviation) or associated estimates of uncertainty (e.g. confidence intervals)
☐	☒	For null hypothesis testing, the test statistic (e.g. *F*, *t*, *r*) with confidence intervals, effect sizes, degrees of freedom and *P* value noted<br>*Give P values as exact values whenever suitable.*
☒	☐	For Bayesian analysis, information on the choice of priors and Markov chain Monte Carlo settings
☒	☐	For hierarchical and complex designs, identification of the appropriate level for tests and full reporting of outcomes
☒	☐	Estimates of effect sizes (e.g. Cohen's *d*, Pearson's *r*), indicating how they were calculated
☐	☒	Clearly defined error bars<br>*State explicitly what error bars represent (e.g. SD, SE, CI)*

Our web collection on statistics for biologists may be useful.

Software and code

Policy information about availability of computer code

Data collection	Flow cytometry data were collected and scored using CytExpert software version 1.2.8.0 (supplied with a Beckman Coulter CytoFlex flow cytometer).
Data analysis	Common bioinformatic and statistical analysis software packages were used, including: R, NCBI BLAST, Samtools, Picard, GATK, FALCON, freebayes BLASR, Quiver, arrow, PBJelly, RepeatMasker, RepeatModeler, RepeatScout, Tandem Repeats Finder, Salmon, gmap, HOMER, bowtie, Juicebox, Assembly Tools. Version numbers and specific parameters used during run-time are provided in the methods when appropriate. All custom software related to the Hi-C assembly is open source and available on the Aiden Lab GitHub page, as indicated in the methods.

For manuscripts utilizing custom algorithms or software that are central to the research but not yet described in published literature, software must be made available to editors/reviewers upon request. We strongly encourage code deposition in a community repository (e.g. GitHub). See the Nature Research guidelines for submitting code & software for further information.

Data

Policy information about availability of data

All manuscripts must include a data availability statement. This statement should provide the following information, where applicable:

- Accession codes, unique identifiers, or web links for publicly available datasets
- A list of figures that have associated raw data
- A description of any restrictions on data availability

Data availability statement. All raw data have been deposited at NCBI under the following BioProject Accession numbers: PRJNA318737 (Primary Pacific Biosciences data, Hi-C sequencing primary data and processed contact maps, whole-genome sequencing data from a single male (Fig. 4d), and pools of male and females (Fig. 3d), Bionano optical mapping data (Fig. 3c and Fig. 4c), and 10X linked-read sequences (Extended Data Fig. 8a and Supplementary Data 21)); PRJNA236239 (RNA-seq reads and de novo transcriptome assembly, Extended Data Fig. 2c, d and Supplementary Data 4, 5, 7, 9); PRJNA209388 (RNA-seq reads for developmental time points, Fig. 1h and Supplementary Data 4–6, 9); PRJNA419241 (RNA-Seq reads from adult reproductive tissues and developmental time points, Verily Life Sciences Fig. 1h and Supplementary Data 4, 5, 8, 9); PRJNA393466 (full-length Pacific Biosciences Iso-Seq transcript sequencing); PRJNA418406 (ATAC-Seq data from adult female brains at three points in the gonotrophic cycle, Extended Data Fig. 2c, d and data not shown); PRJNA419379 (whole-genome sequencing data from colonies Fig. 4d and Extended Data Fig. 9a, b); PRJNA399617 (RAD-Seq data Fig. 5a-d); PRJNA393171 (exome sequencing data Fig. 5e-g). Intermediate results related to the AaegL5 assembly are also available via GitHub (http://github.com/theaidenlab/AGWG-merge) and have been uploaded to GEO (GEO Record: GSE113256). The Hi-C maps are available via http://aidenlab.org/juicebox. The complete mitochondrial genome is available as Genbank accession MF194022.1, RefSeq accession NC_035159.1. The final genome assembly and annotation are available from the NCBI Assembly Resource under accession GCF_002204515.2.

Field-specific reporting

Please select the best fit for your research. If you are not sure, read the appropriate sections before making your selection.

☒ Life sciences ☐ Behavioural & social sciences ☐ Ecological, evolutionary & environmental sciences

For a reference copy of the document with all sections, see nature.com/authors/policies/ReportingSummary-flat.pdf

Life sciences study design

All studies must disclose on these points even when the disclosure is negative.

Sample size	Sample sizes for genome variability analysis via SNP-chip (Fig. 1c) were determined according to previously published work (Evans et al., 2015 PMID 25721127). Sample sizes for genome size determination (Fig. 1d) were determined according to the standards of the field (see Hare and Johnston, 2011 PMID 22065429). Samples sizes for FISH were determined according to the standards of the field (see Timoshevskiy et al., 2012 PMID 23007640). Sample sizes for dengue virus competence (Fig. 5b-d and Extended Data Fig. 10a), pyrethroid resistance (Fig. 5e-g) and larval motility Ext. Data Figure 10c) were determined by the limited availability of animals, biological or chemical reagents. Bioinformatic analyses were performed with all available data.
Data exclusions	None
Replication	Replication does not apply to the primary results of this paper - it was not feasible to independently resequence/reassemble the genome twice within the scope of the funding available to us.
Randomization	Randomization was not performed in this study. Samples were divided into experimental groups based on species, strain or biological phenotype according to the criteria listed in the methods.
Blinding	Blinding was not performed for this study. The diversity of sourcing of samples and data precluded centralized collection and blinding of biological material or sequencing data.

Reporting for specific materials, systems and methods

Materials & experimental systems

n/a	Involved in the study
☒	☐ Unique biological materials
☒	☐ Antibodies
☒	☐ Eukaryotic cell lines
☒	☐ Palaeontology
☐	☒ Animals and other organisms
☐	☒ Human research participants

Methods

n/a	Involved in the study
☒	☐ ChIP-seq
☒	☐ Flow cytometry
☒	☐ MRI-based neuroimaging

Animals and other organisms

Policy information about studies involving animals; ARRIVE guidelines recommended for reporting animal research

Laboratory animals	Male mosquitoes at pupal stage (6-7 days post-hatching) were used to generate the high molecular weight DNA for the primary assembly, Hi-C data, Illumina sequencing data and Bionano optical mapping data. Male and female pupal or adult mosquitoes of various ages were used for all other data collection. Specific details are provided in the methods. Established laboratory strains used include: Aedes aegypti: LVP_AGWG (Rockefeller University), LVP_ib12 (Virginia Tech and Notre Dame), LVP_MR4 (Centers for Disease Control), Rockefeller (Johns Hopkins), Ho Chi Minh City Vietnam (Yale University), New Orleans USA (Yale University), Uganda (Princeton University), Kamphaeng Phet Province Thailand (Institut Pasteur), Viva Caucel Mexico (Colorado State), Clovis USA (Verily Life Sciences), Innisfail Australia (Verily Life Sciences), Puntarenas Costa Rica (Verily Life Sciences), Liverpool (Verily Life Sciences). Aedes mascarensis: Mauritius (Yale University).
Wild animals	Field-collected mosquitoes (Aedes aegypti) were obtained from locations in Australia, Cameroon, Florida, Gabon, Mexico, and Thailand. All appropriate local permits were in place to authorize such collections. Mosquitoes were trapped as adults in the field, or as eggs or larvae reared to adulthood in field laboratories, and euthanized by placement into 100% ethanol to preserve genomic DNA. These animals were shipped as dead samples to the investigators who carried out the analysis.
Field-collected samples	Field-collected mosquitoes (Aedes aegypti) were obtained from locations in Australia, Cameroon, Florida, Gabon, Mexico, and Thailand. All appropriate local permits were in place to authorize such collections. Mosquitoes were trapped as adults in the field, or as eggs or larvae reared to adulthood in field laboratories, and euthanized by placement into 100% ethanol to preserve genomic DNA. These animals were shipped as dead samples to the investigators who carried out the analysis.

Human research participants

Policy information about studies involving human research participants

Population characteristics	Work with human subjects was covered under Rockefeller IRB protocol LVO-0652 (Laboratory of Leslie Vosshall). Only one subject participated in this study as a source of blood for mosquitoes.
Recruitment	One of the authors was the subject for this work, and the subject's participation followed vetting by Rockefeller University administration officials that no coercion by the laboratory head, Leslie Vosshall, to participate in this study had taken place. Written informed consent was obtained prior to enrolling the subject in this study.
