# Supplementary material for: Improved reference genome of Aedes aegypti informs arbovirus vector control
Source: Nature. 2018 Nov 14;563(7732):501–7. doi: 10.1038/s41586-018-0692-z (PMC6421076; doi:10.1038/s41586-018-0692-z)
Supplement: Supplementary file 3 — This file contains Supplementary Data 1-24 and a detailed guide for the datasets [file 41586_2018_692_MOESM3_ESM.zip › 41586_2018_692_MOESM3_ESM/SI Guide.pdf]

Matthews et al.,
Improved reference genome of *Aedes aegypti* informs arbovirus vector control

SI Guide

Supplementary Data 1 - Ext Fig 2a - Bionano analysis of gap structure.xlsx
Estimates of gap sizes in the AaegL5 assembly from alignment of Bionano optical maps
Microsoft Excel format

Supplementary Data 2 - Fig 1 - TE - table of TE and repeat content.pdf
Data table listing the composition (in percent) of the AaegL5 genome assembly by class and family of transposable element and other repetitive sequences
Adobe PDF format

Supplementary Data 3 - Fig 1 - TE - repeat content by chromosome and scaffold.xlsx
Data table listing the percentage of repetitive sequence content for each chromosome and unplaced scaffold in the AaegL5 assembly
Microsoft Excel format

Supplementary Data 4 - Fig 1 - gene expression - library information.csv
Library metadata for all RNA-Seq libraries aligned to AaegL5 assembly
Plain text, comma-separated-values (csv)

Supplementary Data 5 - Fig 1 - gene expression table alignment statistics all libraries.xlsx
Alignment statistics for all RNA-Seq libraries aligned to AaegL5 assembly
Microsoft Excel format

Supplementary Data 6 - Fig 1 - gene expression TPM Akbari NCBI Gene ID.csv
Gene expression in transcripts per million (TPM) for RNA-Seq libraries from developmental timepoints[71]. Reads were aligned to the AaegL5 assembly and quantified using NCBI RefSeq Annotation version 101 (AaegL5.0)
Plain text, comma-separated-values (csv)

Supplementary Data 7 - Fig 1 - gene expression TPM Matthews NCBI Gene ID.csv
Gene expression in transcripts per million (TPM) for RNA-Seq libraries from adult tissues[18]. Reads were aligned to the AaegL5 assembly and quantified using NCBI RefSeq Annotation version 101 (AaegL5.0)
Plain text, comma-separated-values (csv)

Supplementary Data 8 - Fig 1 - gene expression TPM Verily NCBI Gene ID.csv
Gene expression in transcripts per million (TPM) for previously unpublished RNA-Seq libraries (Verily Life Sciences) from developmental timepoints and adult reproductive tissues. Reads were aligned to the AaegL5 assembly and quantified using NCBI RefSeq Annotation version 101 (AaegL5.0)
Plain text, comma-separated-values (csv)

Supplementary Data 9 - Fig 1 - L3 vs L5 table of alignment results.xlsx

Table of pseudoalignment results (percent of reads mapped to annotated transcripts) for all RNA-Seq libraries for the AaegL3.4 and AaegL5.0 (RefSeq version 101) geneset annotations
Microsoft Excel format

Supplementary Data 10 - Fig 1 - merged paralogs.tsv

NCBI GeneIDs and matched annotations from VectorBase AaegL3.5 for genes where multiple partially or fully redundant AaegL3.5 genes were collapsed into a single new gene annotation. Column 3 “is_best” designates the best matching VectorBase gene corresponding to each NCBI GeneID
Plain text, tab-separated-values (tsv)

Supplementary Data 11 - Fig 1 - merged partial.tsv

NCBI GeneIDs and matched annotations from VectorBase AaegL3.5 for genes where multiple non-overlapping AaegL3.5 genes were merged into a single new gene annotation. Column 3 “is_best” designates the best matching VectorBase gene corresponding to each NCBI GeneID
Plain text, tab-separated-values (tsv)

Supplementary Data 12 - Ext Fig 2e - BAC clone accessions and mapping locations.csv

Positions of 88 BAC-ends mapped to the AaegL5 assembly and the position of the clones when hybridized to chromosomes
Plain text, comma-separated-values (csv)

Supplementary Data 13 - Gene family annotations - Proteases.xlsx

Coordinates and summary of changes to metalloproteases and serine genes
Microsoft Excel format

Supplementary Data 14- Gene family annotations - Ext Fig 2f - Opsins and GPCRs Annotation Table.pdf

Coordinates and summary of changes to opsins and biogenic amine related genes
Adobe PDF format

Supplementary Data 15 - Gene family annotations - Ext Fig 2f - Biogenic amine-binding G protein-coupled receptor peptide sequences.pdf

Peptide sequences of biogenic amine-binding G protein-coupled receptor (GPCR) gene products. Non-synonymous substitutions between gene models predicted from the AaegL5 assembly compared to those predicted from the AaegL3 assembly (aqua shading); amino acid sequence unique to gene model predicted from the AaegL5 assembly (grey shading); amino acids associated with functional GPCRs in other species (olive shading)
Adobe PDF format

Supplementary Data 16 - Gene family annotations - Ext Fig 2f - opsin peptide sequences.pdf

Peptide sequences of opsin gene products. Non-synonymous substitutions between gene models predicted from the AaegL5 assembly compared to that predicted from the AaegL3 assembly (aqua shading); amino acid sequence unique to gene model predicted from the AaegL5 assembly

(gray shading); amino acids associated with functional G protein-coupled receptors in other species (olive shading)
Adobe PDF format

Supplementary Data 17 - Fig 2 - Chemoreceptor Annotations Metadata.xlsx

Metadata associated with each gene and alternatively spliced transcript in the AaegL5 geneset. Note that AaegL5 sequences for 'fixed' and 'corrected' genes have loss-of-function mutations that we inferred to be the result of within-strain polymorphism and/or sequence/assembly errors. We provide the coordinates for these genes in AaegL5 but used intact sequences (found in the NCBI TSA database, AaegL3, or apparent in short-read Illumina data from genome strain) in our analyses and fasta files. We also provide notes on the character and source of each LOF mutation for these genes. More details are available in the Supplemental Methods and Discussion
Microsoft Excel format

Supplementary Data 18 - Fig 2 - Chemoreceptor Annotation.gff3

Coordinates for gene models in the AaegL5 assembly representing manual annotation of all chemoreceptors in the *OR*, *IR*, and *GR* gene families. Note that some genes are classified as pseudogenes here due to loss-of-function mutations that reflect sequencing errors or within strain polymorphism. The sequences for these genes were ‘fixed’ or ‘corrected’ in our analyses and in Supplementary Data 21 and 22
Plain text, general feature format (gff3)

Supplementary Data 19 - Fig 2 - ChemoreceptorCodingSequences.fasta

Coding sequences for *Ae. aegypti* ORs, IRs, and GRs. Sequences modified relative to AaegL5 are indicated on the ID line as ‘corrected’ (minor assembly correction) or ‘fixed’ (major assembly correction). IRs and a few GR isoforms were renamed, and previously published names are included on the ID lines in parentheses
Plain text, fasta-formatted sequence file (fasta)

Supplementary Data 20 - Fig 2 - ChemoreceptorPeptides.fasta

Protein sequences for *Ae. aegypti* ORs, GRs, and IRs, and *An. gambiae* IRs (for which many new genes were identified). Sequences modified relative to AaegL5 are indicated on the ID line as ‘corrected’ (minor assembly correction), ‘fixed’ (major assembly correction), and ‘pseudogene’ (coding sequence repaired with stop codons coded as Z and other pseudogenizing mutations such as indels or splice site mutations coded as X). IRs and a few GR isoforms were renamed, and previously published names are included on the ID lines in parentheses
Plain text, fasta-formatted sequence file (fasta)

Supplementary Data 21 - Ext Fig 8a - Structural variants details.csv

Summary table of structural variants identified by linked-read sequencing of two individual mosquitoes with analysis performed using Long Ranger and GROC-SVs software. Note that alignments were performed prior to standardizing gap length for the assembly at 100N; coordinates were subsequently transformed to the final NCBI reference by BLAST of surrounding regions (reported in the columns labelled ‘AaegL5 final coordinates’)
Plain text, comma-separated-values (csv)

Supplementary Data 22 – Ext Fig 8 - AaegL5 *HOXC* gene table.xlsx

Coordinates and summary of changes to genes in the HOX Cluster

Microsoft Excel format

Supplementary Data 23 - Fig 4 - P450 and GST gene annotations.xlsx

Coordinates and summary of changes to cytochrome P450 and GST gene families

Microsoft Excel format

Supplementary Data 24 - Ext Fig 10d - LGIC sequences and notes.pdf

Revised peptide sequences for *Ae. aegypti* cysLGICs subunits based on the AaegL5 assembly. Lower case letters in the peptide sequences indicate the N-terminal signal peptide as predicted by SignalP 4.1

Adobe PDF format
