# Supplementary material for: Improved reference genome of Aedes aegypti informs arbovirus vector control
Source: Nature. 2018 Nov 14;563(7732):501–7. doi: 10.1038/s41586-018-0692-z (PMC6421076; doi:10.1038/s41586-018-0692-z)
Supplement: Supplementary file 3 — This file contains Supplementary Data 1-24 and a detailed guide for the datasets [file 41586_2018_692_MOESM3_ESM.zip › 41586_2018_692_MOESM3_ESM/Supplementary Data 14 - Gene family annotations - Opsins and GPCRs Annotation Table.pdf]

Matthews et al. Supplementary Data 14

Table 1. Comparative analyses of *opsin* genes predicted from the *Aedes aegypti* AaegL3 and AaegL5 assemblies.

Assembly	VB/NCBI Accession	Supercontig/ Chromosome	bp range	Length (nucleotides)	Length (amino acids)	Max No. exons	No. Splice variants/ Isoforms	% amino acid identity	Notes on the revised annotation
GPRop1 (opsin 1)									
AaegL3	AAEL006498	1.208	1715269-1716390	1122	373	1	1	100	-
AaegL5	LOC5568060 XP_001651997	3	15095953-15097396	1384	373	2	1		
GPRop2 (opsin2)									
AaegL3	AAEL006259	1.197	948069-949187	1119	372	1	1	99	1 non-synonymous SNP in ex2
AaegL5	LOC5567680 XP_001657619	3	63253679-63255160	1418	372	2	1		
GPRop3 (opsin 3)									
AaegL3	AAEL006484	1.208	1726735-1727928	1122	373	2	1	100	-
AaegL5	LOC5568061 XP_001651998	3	15084409-15085900	1420	373	2	1		
GPRop4 (opsin 4)									
AaegL3	AAEL005621	1.165	1775297-1776430	1134	377	1	1	100	-
AaegL5	LOC5566755 XP_001651166	3	19560417-19559287	1355	377	1	1		
GPRop5 (opsin 5)									
AaegL3	AAEL005625	1.165	1782454-1783587	1134	377	1	1	100	-
AaegL5	LOC5566757 XP_001651167	3	19551877-19553332	1356	377	1	1		
GPRop7 (opsin 7)									
AaegL3	AAEL007389	1.252	485115-486311	1140	379	2	1	99	2 non-synonymous SNPs, one in ex1 and the other in ex2
AaegL5	LOC5569125 XP_001652725	1	259865468-259866849	1325	379	2	1		
GPRop8 (opsin 8)									

AaegL3	AAEL009615	1.412	271590-320188	1146	381	5	1	100	-
AaegL5	LOC5572198 XP_021698798	2	169760185-169788904	1499	381	5	1		
GPRop9 (opsin 9)									
AaegL3	AAEL003035	1.75	1344859-1358612	1143; 237	380; 78	2; 1	2	100	-
AaegL5	LOC5576882 XP_001662982	1	130264248-130275937	1496	380	2	1		
GPRop10 (opsin 10)									
AaegL3	AAEL005322	1.151	1942356-1943586	1107	368	3	1	99	3 non-synonymous SNPs: 2 in ex3 and 1 in ex4. Deletion of 126 nucleotides (42 amino acids)
AaegL5	LOC5566350 XP_021702394	2	64921064-64923032	1719	326	4	1		
GPRop12 (opsin 12)									
AaegL3	AAEL005373	1.153	1048301-1134510	1239	412	5	1	92	Completion of 5’ CDS with prediction of an additional 342 nucleotides in ex3 (114 amino acids) and inclusion of a start codon.
AaegL5	LOC5566410 XP_001650802	3	28836549-29027093	2921	526	7	1		

NCBI, National Center Biotechnology Information; VB, VectorBase

Table 2. Comparative analyses of biogenic amine binding genes predicted from the *Aedes aegypti* AaegL3 and AaegL5 assemblies.

Assembly	VB/NCBI Accession	Scaffold/ Chromosome	bp range	Length (nucleotides)	Length (amino acids)	Max. No. Exons	No. Splice variants/ Isoforms	% Amino Acid Identity	Notes on the revised annotation
**Dopamine Receptors**									
GPRdop1 (Dopamine 1)									
AaegL3	AAEL003920	1.1	21431-279934	1239	412	4	1		37 non-synonymous substitutions: 3 in ex1, 29 in ex2, 1 in ex6 and 4 in ex7<br>X1: Deletion of 7 amino acids at the 5' of ex1. Addition of 31 amino acids: 23 in ex2 and 8 in ex7<br>X2: Deletion of 7 amino acids at the 5' end of ex1. Addition of 23 amino acids only in ex2
AaegL5	LOC5563724 (X1)[a] XP_021693210	1	249730382-250104240	1918	436	8	2[b]	96	
	LOC5563724 (X2)[a] XP_021693211	1		1566	428				
GPRdop2 (Dopamine 2)									
AaegL3	AAEL005834	1.176	1175027-1317675	1431	476	3	1	100	-
AaegL5	LOC5567099 XP_001651499	1	116933928-116935187	2416	476	5	1		
GPRdop3 (Dopamine 3)									
AaegL3	AAEL014373; AAEL017166	1.1093; 1.1134	117534-189099; 10324-10536	972; 213	323; 70	6; 1	1	100; 99	Addition of 275 amino acids: 161 in ex3, 63 in ex4, 17 in ex5 and 33 in ex11.<br>1 non-synonymous substitution in ex8
AaegL5	LOC5564275 XP_021705228	3	86037281-86791700	3714	668	12	1		
**Serotonin Receptors**									
GPR5HT1A (Serotonin 1A)									
AaegL3	AAEL008360	1.319	255496-306119	1506	501	7	1	98	Addition of 80 amino acids: 24 in ex1 and 56 in ex2.<br>2 non-synonymous substitutions in ex6
AaegL5	LOC5570464 XP_021701402	2	42581753-42638533	2402	580	8	1		
GPR5HT1B (Serotonin 1B)									
AaegL3	AAEL017272	1.771	225759-374236	993	330	3	1	100	Addition of 86 amino acids: 17 in ex2, 12 in ex5 and 57 in ex6
AaegL5	LOC23687692 XP_021704615	3	295879868-296086450	3542	416	6	1		
GPR5HT2 (Serotonin 2)									
AaegL3	AAEL017162	1.250	1371280-1525286	1557	519	7	1	99	Addition of 408 amino acids. Deletion of 77 amino acids. 2 non-

AaegL5	LOC23687582 XP_021705609-14	3	123997830-124107829	X1: 4432, X2: 4457, X3: 3807, X4: 3875, X5: 3515, X6: 3648	847	11	6		synonymous substitutions
GPR5HT7A (Serotonin 7A)									
AaegL3	AAEL009573; AAEL016993	1.1801; 1.408	13824-15218; 527553-528947	1395; 1395	464; 464	1;1	1;1	99	1 non-synonymous substitution
AaegL5	LOC5572158 XP_021693679-82	1	282109307-282443182	X1: 4273, X2: 4458, X3: 4249, X4: 4072	464	6	4		
GPR5HT7B (Serotonin 7B)									
AaegL3	AAEL011844	1.621	526913-528331	1419	472	1	1	99	3 non-synonymous substitutions in ex3
AaegL5	LOC5575465 XP_021693677	1	281559502-281884495	4410	472	3	1		
Putative 5HT receptor 1*									
AaegL3	AAEL000528	1.10	585688-586260	462	154	2	1	81	Addition of 412 amino acids to 3’: 25 in ex4, 133 in ex5, 43 in ex6, 42 in ex7 and 169 in ex8
AaegL5	LOC5563693 XP_001647960	3	388283407-388542853	3311	566	8	1		
Putative 5HT receptor 2*									
AaegL3	AAEL015553; AAEL002717	1.3220; 1.65	1465-2616; 2854937-2935086	1122; 1314	373; 437	2; 4	1; 1	97	Addition of 456 amino acids to 5’ and 10 amino acids to the 3’. 1 non-synonymous substitution
AaegL5	LOC5575783 XP_021711581-95	3	124129041-124465172	X1: 3811, X2:4061, X3: 3650, X4: 3719, X5: 3665, X6: 3953, X7: 3792, X8: 3524, X9: 3767, X10: 3517, X11: 3667, X12: 3149, X13: 3442, X14: 3666	839	9	14		
Putative 5HT receptor 3*									
AaegL3	AAEL007644	1.270	558479-559941	804	268	2	1	99	Addition of 258 amino acid residues: 99 in ex2, 44 in ex3 and 115 in ex5.<br>1 non-synonymous substitution in ex5
AaegL5	LOC5569455 XP_001658546	1	186894409-186942332	2059	526	5	1		
**Muscarinic Acetylcholine Receptors**									
GPRmac1									
AaegL3	AAEL017181	1.834	277986-372439	2061	686	4	1	97	Deletion of 17 amino acids at the 5’ end of ex5.<br>8 non-synonymous substitutions: 2 in ex2 and 6 in ex3
AaegL5	LOC23687601 XP_021708563.1	3	285466614-285544711	2478	669	5	1		

GPRmac2									
AaegL3	AAEL015037	1.1412	10253-13572	2955	984	3	1	100	Deletion of 142 amino acid residues at the 5' end of ex1. 6 non-synonymous substitutions in ex2
AaegL5	LOC5565991 XP_021693812	1	289865824-289869428	3542	842	2	1		
**Octopamine/Tyramine Receptors**									
GPRoar1									
AaegL3	AAEL014224	1.1043	81915-265813	1218	405	5	1	70	X1: Addition of 149 amino acid residues in ex6. 67 non-synonymous substitutions: 1 in ex4, 2 in ex5 and 64 in ex6 X2: Addition of 121 amino acid residues: 1 in ex5 and 120 in ex6. 2 non-synonymous substitutions: 1 in ex4 and 1 in ex5
AaegL5	LOC5563924 (X1) XP_021695040[a]	1	32884333-33609615	4932	598	7	2[b]		
	LOC5563924 (X2) XP_021695041[a]			4996	572				
GPRoar2									
AaegL3	AAEL004396	1.117	640125-789723	1740	579	5	1	99	Addition of 141 amino acids: 138 in ex3 and 3 in ex6. 1 non-synonymous substitution in ex3
AaegL5	LOC5564696 XP_021692997	1	236109203-236416433	3657	720	7	1		
GPRoar4									
AaegL3	AAEL005945	1.181	306823-327934	1062	353	4	1	99	Addition of 211 amino acid residues: 178 in ex2 and 33 in ex3. Deletion of 19 amino acid residues: 14 in ex5 and 5 in ex7. 2 non-synonymous substitutions: 1 in ex6 and 1 in ex7
AaegL5	LOC5567283 XP_021693342	1	258662498-259072990	3315	545	7	1		
**Unclassified Biogenic Amine Binding Receptors**									
GPRnna19*									
AaegL3	AAEL004245	1.111	833628-834518	891	296	1	1	100	Addition of 142 amino acid residues: 42 in ex1, 53 in ex2 and 47 in ex3. 1 non-synonymous substitution in ex3
AaegL5	LOC5564406 XP_021694244	1	105992061-106014836	1806	438	3	1		

*Indicates unresolved gene model requiring verification by molecular analyses
[a]indicates protein isoforms
[b]indicates transcript variants
