# Supplementary material for: Improved reference genome of Aedes aegypti informs arbovirus vector control
Source: Nature. 2018 Nov 14;563(7732):501–7. doi: 10.1038/s41586-018-0692-z (PMC6421076; doi:10.1038/s41586-018-0692-z)
Supplement: Supplementary file 3 — This file contains Supplementary Data 1-24 and a detailed guide for the datasets [file 41586_2018_692_MOESM3_ESM.zip › 41586_2018_692_MOESM3_ESM/Supplementary Data 15 - Gene family annotations - Biogenic amine-binding G protein-coupled receptor peptide sequences.pdf]

Matthews et al., Supplementary Data 15

Biogenic amine genes predicted from the *Aedes aegypti* L5 Genome Assembly

Protein sequences

Dopamine Receptors

GPRdop1

Isoform_X1

>XP_021693210.1 dopamine receptor 1 isoform X1 [Aedes aegypti]
MNYLVVSATTICSNLTAVANQTDGPGDGGGSGGGGGGGGTGHDDGDDDDVDALSPLSMSCLGVFLSIVIFLSVAGNILVCIAIYTE
RSLRRIGNLFLASLAIADLFVASLVMTFAGVNDLLGYWIFGAQFCDTWVAFDVMCSTASILNLCAISLDRYIHIKDPLRYGRWVTR
RVAIGTIVVIWLLAALVSFVPISLDLHRDKRDETDTSLIINGVKYETCALDLTPTYAVVSSCISFYVPCIVMIGIYCRLYCYAQKH
VKSIRAVTRPGEISEKRYRSIRRPKPSKNKLKLRQLAHHASSPYHVSDHKAAITVGVIMGVFLVCWVPFFCVNIIAAFCKTCIGPQ
TFKVLSWLGYSNSAFNPIIYSIFNTEFREAFKRILTTRSSWCCAQEMGNIYPRHSDRYVTDYAAKNVVMNSGRSSADLEQDEQEDQ
EYFSIF

Isoform_X2

>XP_021693211.1 dopamine receptor 1 isoform X2 [Aedes aegypti]
MNYLVVSATTICSNLTAVANQTDGPGDGGGSGGGGGGGGTGHDDGDDDDVDALSPLSMSCLGVFLSIVIFLSVAGNILVCIAIYTE
RSLRRIGNLFLASLAIADLFVASLVMTFAGVNDLLGYWIFGAQFCDTWVAFDVMCSTASILNLCAISLDRYIHIKDPLRYGRWVTR
RVAIGTIVVIWLLAALVSFVPISLDLHRDKRDETDTSLIINGVKYETCALDLTPTYAVVSSCISFYVPCIVMIGIYCRLYCYAQKH
VKSIRAVTRPGEISEKRYRSIRRPKPSKNKLKLRQLAHHASSPYHVSDHKAAITVGVIMGVFLVCWVPFFCVNIIAAFCKTCIGPQ
TFKVLSWLGYSNSAFNPIIYSIFNTEFREAFKRILTTRSSWCCAQEMGNIYPRHSDRYVTDYAAKNVVMNSGRSSADLEQNERT

GPRdop2

>XP_001651499.3 dopamine receptor 2 [Aedes aegypti]
MNNATDFYWELSQLIRFSNLSYYNVSNGGETSVTLDAVNCCTTEPNFSEYLDALPNDRAGLLAFLFLFSFATVFGNSLVILAVIRE
RYLHTATNYFVTSLAVADCLVGLVVMPFSALYEVLQNTWFFGEDWCDIWRSLDVLFSTASILNLCVISLDRYWAITDPFSYPMKMT
RKKAAALIAAVWICSSAISFPAIVWWRAAREGDMPPYKCTFTEHLGYLVFSSTISFYLPLLVMVFTYCRIYRAAAIQTRSLKLGTK
QVLMASGELQLTLRIHRGGTTRERPNHQQQQPPQHQTHQHQDHQQHTANSTPEEPDEEPLSALQNNGLGGRHRTHMGKNFSLSRKL
AKFAKEKKAAKTLGIVMGVFIVCWLPFFVVNLLSGFCMQCIAHEEIVSAVVTWLGWINSSMNPVIYACWSRDFRRAFVRILCVCCP
RKIRRKYQPTMRSKASQRFASRRCYSTCSLHGIQQVRQNSCEQTYI

GPRdop3

>XP_021705228.1 LOW QUALITY PROTEIN: dopamine D2-like receptor [Aedes aegypti]
MVTVNDSMVPGWDSPDYFRLFSEGSAASNESAVVRPGRSSPFGGLSPANQSGSNGGVVVVGGSSANSSNSSASPAAVANGVYQVSF
YGDRNETRLGAGAASGGDRERGGSNFMLLLEDFGEYFYNYNGTGPPNDTLGLGGTSAGFPINCTVANTTCDLPSEDHYNYWGLILV
LFPILTLFGNVLVILAVCRERTLQTVTNYFIVSLALADLLVAVVVMPFAVYVLVNGAWTLPNFVCDFYIAMDVICSTSSIFNLVAI
SIDRYIAVTQPIKYAKHKNSRRVCLTILLVWAISAAIGSPIVLGLNNTPDRVPDLCVFYNSDFIVYSSLSSFYIPCIIMVFLYWNI
FKALRTRAKKQRAARKPHISEITAGGSLIENIAQTKVATETHLDGSTKSAGSCKILPDEAPTNTASGSNEEDDDNAGSPDIDDCHV
IVNDKSTEFMLATVVEEAGNVMAQITTTQIPSDPNGNHDSGYAPSSIGDVLAANASPPGSPNVAATVIVNRNATLSSRNGSPRKEA
SVTLKPLSLVRCGVQQALSLNRNDSTLSTNSNSRDSTRKDKKSIQQPSRFTIYKVNKASKKKREKSSAKKERKATKTLAIVLGVFL
ICWVPFFTCNIMDAMCTKLDMTCQPGVTAFILTTWLGYMNSFVNPVIYTIFNPEFRKAFKKIMNIE

Serotonin Receptors

GPR5HT1A (Serotonin 1A)
>XP_021701402.1 5-hydroxytryptamine receptor [Aedes aegypti]
MDFHKAPPPRPPSLVGSALTKSNDVYDYIFIDSGSSPDSYLAGELGSFVSDDNISLLLNGTTTTLVNGSDLGNGTTGLGLDEPLAD
VIAMAVTSLILGLMILVTVIGNVFVIAAIILERNLQNVANYLVASLAVADLFVACLVMPLGAVYEISRGWILGPELCDIWTSCDVL

CCTASILHLVAIATDRYWAVTNIDYMHSRTSRRVFTMIFLVWFASVIVSLAPQFGWKDPDYLQRIEQQKCMVSQDIAYQIFATCCT
FYVPLLVILVLYWKIYQTARRRIHRRRPKQPVTGNNNQEETPKQTKSKIRFRLKKKFSNPAKSAASQLGLVEGNSTNTVNTVEDTE
GSSNAERKTGGVETAFSSDEAPTVAAVGNSQIPTVSHEVEHRQLSEPTAVGMEANNNNHNNNSSSNGNSSSGANTQQLSTQQQHQH
QHSSHLSLRPTSNNTLVPPIQHSPTMLSGSHTHIGSSQNIASTPNPHAQVSKRKETLEAKRERKAAKTLAIITGAFVVCWLPFFLT
ALLLPLCESCYINDTVASLFLWLGYFNSTLNPVIYTIFSPEFRQAFKRILFGTHRSTNYRRGKL

GPR5HT1B (Serotonin 1B)

>XP_021704615.1 5-hydroxytryptamine receptor [Aedes aegypti]
MVATAVVLGIVILATVIGNVFVIAAILLERNLQSVANHLILSLAVADLLVACLVMPLGAVYEVSKEWRLGADLCDMWTSSDVLCCT
ASILHLVAIALDRYWAVTDIDYAHQRTARRIGYMIIVIWTLSVLVSIAPLLGWKDPEWEARVYKDLQCIVSQDVGYQIFATASSFY
VPLLVILFLYWRIFLAARKRIRRRQQGKTIVQLIPKPLVTASQPTNAIGGPTANVVGGSGGIAAAVVAVIGRPLPTISETTTAFTN
VSSANTSPEKGSLGNGVERDRIEADPPTADFSTAYPSGQGGASSSSAKPSIMKKKSQSATDSKRERKAAKTLAIITGAFVCCWLPF
FIIAILLPTCTDCDISPLTMSVCLWLGYFNSTLNPIIYTIFSPEFRHAFKRILCGRRHSLRRTRHMGVRHMR

GPR5HT2 (Serotonin 2)

>XP_021705614.1 muscarinic acetylcholine receptor M3 [Aedes aegypti]
MENGQPWSDWCSGNYWSTVRYLLSCANYNNTWTSYWGPDIVCDYNRIALSPGEQPAEVVRNSLKGIAAINTVQSTASSPINASSSI
DHNSVLRVGIECYQNFKCILKSVTPSSGNSSSIVGNQSNSDAVRWTDALYECNPLMATDKQDNLLKECITSYNATIVARNIVKPVS
ELLLRLPKLSLEMLASRTGILSNDTSATATTAGQPCAPDITRVSYHHYQLICNNSAGRFTFYENDSFDLLNSSTSALLEPVQCLLN
VQYDDVINQLTGIVFPNGTTTTVTGGSSLMSFGTRYEWSFLFVILFIFAGGLGNILVCLAVALDRKLQNVTNYFLLSLAIADLLVS
LFVMPLGAIPGFLGYWPFGVTWCNIYVTCDVLACSASILHMCFISLGRYLGIRNPLGSRHHSTKRLTGIKIVLVWLLAMLVSSSIT
VLGIINKYNIMPGPQECVINNRAFFVFGSLVAFYIPMVMMVVTYALTVQLLRKKARFLEEHPEGELFRRLGGRLSKTKHPSAGNGG
NSGVGCQSDDSICLAMKNPLERRKNNINNNSRMLPFGGTAPWQLHGLNSDRIGTISASTQAVSTAGTYSNGGTDPAGIVSSSTLSG
RSNVGGRARRAQYHHNHHLPHHHHHQQHNHHHYHRSDLSTQSSMRTCDQSTQTPENIERETRRQRFCSFRLHLNSVPTPSINFNLK
FLASKKRTNLSANAVATEQKATKVLGLVFFTFVFCWAPFFILNIIFAAWPDLEVPDRIVNICLWLGYVSSTINPIIYTIFNKTFRA
AFIRLLRCRCERSRRPSRYRSVTDSRGAVSLCTPSALPLAISLQGAPLLTPTSTQVTPLSDFRGSYEITDDDC

GPR5HT7A (Serotonin 7A)

>XP_021693682.1 5-hydroxytryptamine receptor 1 [Aedes aegypti]
MDPTVFPLLSTLLQQSSAQVLPIGDGPTSTVASGVAEVAIINATATINFLEYLLTGNSSSASVSATAIATSLPALVDRLTPTSSTS
SLLDELGGTSESSPAEPVNVLTIQTIVISIVLLAVIIGTIVGNVLVCVAVCLVRKLRRPCNYLLVSLAISDLCVAVLVMPPALLYE
VLEEWKFGTVFCDIWVSFDVLSCTASILNLCAISVDRYWAITKPLEYGVKRTPRRMIACIVLVWLVAACISLPPLLILGNEHMTNG
QPSCSVCQNFFYQIYATLCAFYIPLAVMLFVYFQIFRAARRIVNEEKRAQKHLETAINGSATTPEKKLSAGGTVLVATPQHKRLRF
QLAKERKASTTLGIIMSAFTVCWLPFFILALVRPFLGEDHHLLSSLFLWLGYANSLLNPIIYATLNRDFRKPFQEILFFRCSSLNN
MMREDFYHSQYGDPGSQRLVMAANDGGGARESFL

GPR5HT7B (Serotonin 7B)

>XP_021693677.1 5-hydroxytryptamine receptor 1 [Aedes aegypti]
MDALALSRLVNEITIGSQEGLYAQRLQEQQSYSQVPDIEATAISAVPFALLTLNQTLSHSLAGAANGGFDGGLSPAVAAAAAVTST
STTTAGSIIASTLAGKALDHEPEIDTIRKVIICIVLLAVIFGTIVGNILVCVAVCLVRKLRRPCNYLLVSLAVSDLCVACLVMPPA
LMYEVLGEWNFGRVFCDIWVSFDVLSCTASILNLCAISVDRYWAITKPLEYGVKRTPRRMMLCVALVWLAAACISLPPLLILGNKH
TIGEGPDQRPFCAVCEDVGYQIYATLGSFYIPLAVMLFVYYQIFRAARRIVKDEKRAQTRLENSLAVDKNQAMLKPPEPLSSSVGS
PHQKKLRFQLAKERKASTTLGIIMSAFTICWLPFFILALVRPLMDDDYPTLSSFFLWLGYANSLLNPIIYATLNRDFRKPFQEILY
FRCSNLNILMREDFYHSQYGEPGSQRFVLDNEGQHTARESFL

Putative 5HT receptor 1

>XP_001647960.3 5-hydroxytryptamine receptor 1D [Aedes aegypti]
MDLKAGKHLVAPAINNISCSKQSFASGFFAQIGPLDVLQALFIVFLTFLVISANLMVIVVINSRRYAAYIHPQPRYLLTSLALNDL
AIGLLIIPFGALPALLHCWPYGEIFCQIQALLRGALSQQSAVILVCMAVDRYLCVLHPRIYHKRSSKKGCVAILSVTWILCLTCFG
LLVLPKGYYFNKSGLLACEPFYSKSSYRILSSCALYFPTTMVLMYCYGSSFHANRYRLTTPASVNAAAAALSSMQKQHQQQQQQGS
SEGNGGGHPGGDGGCGGATTTTTTEIGTINNSSCAGTPTAISVVTPSSLSFSEKLADHEQRLNGSTSRTMAAISLGFIVIITPWTI
QEIVATCTGSKIPPSIDFCVTWIALSSSFWNPFLYWLLNARFRRICKEMLTSKCTSSQTSLEEKCSISLEYDQHLTALPLPPGPPP
ATICRSANHTPRPDIECTERYWSGIPERTYSTGSINALYRSTSSSSTAYHQTTLHRHHHRHRHYHQNGSNGSNHHSSNSIHKTPMD
DEKDGDFSVIFPMTKSDPAMSCEHELHDINCAKNRAFFLGFNHHQALPDT

Putative 5HT receptor 2

>XP_021711581.1-95.1 uncharacterized protein LOC5575783 [Aedes aegypti]
MRPKLANPTSNSDLLEMWLWSVSKPINYFKFANQAANDYRAGYGWAPLRLNLSYHLSTTTVDSVLSVVDDNDRDGTAFDEDEDDDG
TNALNNYWALLAIVLVFGTAAGNILVCLAIVWERRLQNVTNYFLMSLAITDLMVALSVMPLGILTLVRGYFPLQSEYCLVWICLDV
LFCTASIMHLCTISVDRYLSLRYPMRFGRNKTRKRVVLKISFVWLLSIAMSLPLSLMYSQNHASVLVEGTCQIPDPVYKLVGSIVC
FYIPLCVMLITYTLTVRLLAQQSQNLCGAAGAVASATGSWSSGWLGQAPVFDRRNTWKRIIKLSLPSTPNHAHSAASTDTELSTLD
NHDLWLLESSIPEPTPSTMTALHQFGEEMLKLSRGLETVTNSTHNNNSNINNGNSNKNGNTIRNGEQQSRESGCPSLLLPTTIAHH
GTSEIRNGPGTDTDGKKFPLRKKSVSMATKPQPPKKRRSSTSAWINRRNSATPPSTAVRKRFKSLPFAVLPPEGVDPDIIVDEFYL
QEALLRNKENSHRHTDDDVTQAMTENDDSSRSYSETSEGTSVNLLKLPPPCKCPYFGESCSKQQYEGIRPAEIKIVKTTSNFSSIS
NFDTQIYSNISISSQPTRSTSSSLSTLPASSVVSPNKKGNHLKSVSVVTWDSRRHQRRGSSFGGARTSLLLTPTKPSTSSTSLRRS
ATLRHHNESTPSIVVSGVAPKKNSSSPCLIQRQATIRSHHSRNSSVISRNSSRHGRIILLEQKATKVLGVVFFTFVVLWAPFFVLN
LLPSVCKECEDSINHWVFEFVTWLGYASSMVNPIFYTIFNKAFRDAFKKVLLCRYGSKPAWQPSS

Putative 5HT receptor 3

>XP_001658546.3 octopamine receptor 1 [Aedes aegypti]
MTDVPQPPDYWQLRYFLYLESPSSIGSTSSNRQPYSHEPDKMLSSWDLSAPVPMSENMLSNAEQTQLLLYDILIPLLGSLIIVMNV
AVVTSSLLLLRKGQQPYTTYLFLGNVAASDLLTGFAVLYGQYAPKEIRGEDNCAILIGLIVSTTIVSVYSIGLIAIDRYLYIVYGL
QYQRYITPSRAKLLIAATWLIGLIIGFLPAFGWRGDTDGGRVCWFVRLAPPALIILTTVVGIIPLIVVIVLYSIILQKALRRVAQL
KKAGLEQQGALAGNLRLFRGGGTAAASAPVAAEQPLADEHRKPTKCFRCCKKRSPKTIDGRNTGKHPTKWKAIKVVMLTTGCFVVT
WLPYFIASTMFVLCDPKTNQDLCRGLQFAIASPLAILGFTNSLLNPLIYAWWHNGFRTSMKKLWRKMCSCCCQPCDNKSFSQDPAQ
ESSARTASSTTGSNRAVIGTTSDVVIGGNGASQQSRSSDATTSTTTNTSFTASPQYRISRSNNTSNRSMPTYNSNTSDLENRITDT
DIDRSVQTRL

Muscarinic acetylcholine receptor

GPRmac1

>XP_021708563.1 muscarinic acetylcholine receptor DM1 [Aedes aegypti]
MAYYDTRTTVDSFLLSRYSAATGIAGSTGTMGSVEDMFFNITPTSSTSLTTALSSSTIGSPGGNETESLAEIEPTYSLVQIIILGI
IATVLSILTVAGNVMVMISFKIDKQLQTISNYFLFSLAIADFAIGLISMPLFSVTTLLGYWPLGPHICDTWLALDYLASNASVLNL
LIISFDRYFSVTRPLTYRAKRTTTRAAIMIGAAWGISLLLWPPWIYSWPYIEGKRTVPEKECYIQFIETNHYITFGTAIAAFYVPV
TVMCFLYYRIWRETKKRQKDLKNLTGDRKKDSSKRSNSSDENTAVNHSGAIAQLTNAEPDGWRRPRSESSADAESVYMTNAVISDS
GYGHGMLSRRSSVISTFTLKKPTTFFGGIKEWCIAWWHSGREDSDEYGYDAEEPSDLGCTSLNVIRDPYGTTGRGNVVSMIPDISP
TPMRPTLPPMAHLQDMHGPRDSRSLPNSNRLGSRSVSQDSVYTILIRLPPEGGSGDERAPSIKMIQDDVPMSMTVPPRRPLPSRDS
DYIIPVGRRQSTATTDIRLPLTTKIIPKPLSKQQQIHQANMLQTARQVKKKKKSQEKRQETKAAKTLSAILLSFIITWTPYNILVL
LKPLTACTRCIPQELWDFFYALCYINSTINPVCYALCNASFRRTYVRILTCKWHTRNREAMTRGVYN

GPRmac2

>XP_021693812.1 probable muscarinic acetylcholine receptor gar-2 [Aedes aegypti]
MDMGDGIGPVLPPFELWQTVLIAICLAICIILTIGGNILVLLAFIVDRSIRQPSNYFIASLAATDMLIGTVSMPFYTVYVLMGYWD
LGPLLCDLWLSVDYTVCLVSQYTVLLITIDRFCSVKIAAKYRSWRTKNKVIWMVTITWIIPALLFFISIFGWEHFVGYRDLAPGEC
TVQFLKDPVFNTALIIGYYWTTLVVLFVLYGGIYKTAYDMQKKSEAKQRKMQSMVALGNAMTGLTGQAAGIGISKTQSTLLSQDKP
LPLPTTPASNMMHPTTLALASNQMQKEEAGSSRKSSMPKPKDEKVDEQRKSKEEKEGDKSERSSSPAFDSDDESSANTKQQQEEAR
VAKKRTSLAGLLVGASATVLSNRYNNGAVKTTTTPTSEQAKPKRSPSNPAKNEGLPKITESSIMDSDNTDGKPQVSPLKPTPVQSP
ISPVDIEPVVVSKSERAPEISHTILTPPYGFQGSPRTSESPPSKSDRPHSIISQGDSTVTYDAMVSMDGADLRFMDESSVVVPSPQ
YESPPSAFAIVQPRSPAHSAAQSIANPSLLQKALIRATAQSQQPPPTVLLRTIDVGPLSPTSQQNSIQPINKFSTTVTVVSQNPDK
PVTVLNNSYTNHSTVDQALPQPSQMDSPSLNSRLQSNNSTPVSSTPSNPKPSMSLAVVDQSTAATSTTTSSSTAAPAVTSPPPQTN
ALPSTTTVQTQVSSSTTNNTPSGATITINPCLERQTSSKRDFIRSIGKRLKGNKKSGTLGVGRQKSKSENRARKAFRTISFILGAF
VACWTPYHVLALVVGFCRNPPCINEHLFMFSYFLCYANSPMNPFCYALANQQFKKTFMRILRGDLHMT

Octopamine/Tyramine receptors

GPRoar1

Isoform_X1

>XP_021695040.1 octopamine receptor Oamb isoform X1 [Aedes aegypti]
MNATECNSLIASVEWTEPRKILLLAILIFIDILVIVGNTLVVAAVTSSHKLRSVTNFFIVSLAVADLLVGLAVLPFSATWEVFKVW
IFGDVWCRVWLAVDVWMCTASILNLCAISLDRYVAVTRPVTYPSIMSTRRAKSLIAGLWVLSFVICFPPLVGWKEQKVKENLLLPY
GNHTLVSMSPSPVTSTSSYSSSPSVSSISSSSSSPSTGTSSAFSTITSSSIAESWEVGGMGETSPNPPPCPWTCELTNDAGYVVYS
ALGSFYIPMFVMLFFYWRIYRAAERTTRAINQGFRTTKGMGTRFDDNRLTLQIHRGRGSTASAHGAPTIGGSAAGVLGSAHHESPH
SNGSTHSTTTSIGSASPERLSRYMTRCKNHEKIKISVSYPSTENLNQSSSAGEGSKLLYAVHYSSNGGREHNASHIFRRPSKEQSG
SGQYLTVDGQGRSLLSPRSSKRMGKRNIKAQVKRFRMETKAAKTLAIIVGLFILCWLPFFTMYLIRPFCDNCINDLLFSIVFWIGY
CNSAINPMIYALFSKDFRFAFKRLICRCFCSAEAIPRPASRRGSDMSQIRMHGARTPSISPSAAAQSIGDDSDPVGDLSDSR

Isoform_X2

>XP_021695041.1 octopamine receptor Oamb isoform X2 [Aedes aegypti]
MNATECNSLIASVEWTEPRKILLLAILIFIDILVIVGNTLVVAAVTSSHKLRSVTNFFIVSLAVADLLVGLAVLPFSATWEVFKVW
IFGDVWCRVWLAVDVWMCTASILNLCAISLDRYVAVTRPVTYPSIMSTRRAKSLIAGLWVLSFVICFPPLVGWKEQKVKENLLLPY
GNHTLVSMSPSPVTSTSSYSSSPSVSSISSSSSSPSTGTSSAFSTITSSSIAESWEVGGMGETSPNPPPCPWTCELTNDAGYVVYS
ALGSFYIPMFVMLFFYWRIYRAAERTTRAINQGFRTTKGGKKSNNFDDSLTLRMHRGKGVGALMASTMRTHNMLIEGSAVRPSSVS
NASSTANANPQISISCPSGNTSHLTSPTVSLGSSLQINLPSQHNGGSSSAGGSGSGRYPPSRPNSRRKSVFKEAPAGISRDLSPNS
SKKLGKKHLKVQVKRFRMETKAAKTLAIIVGGFIICWLPFFTMYLIRAFCDDCINDTLFSILFWLGYCNSAVNPFIYALFSRDFRQ
AFKRIIMRCFCWKRFKINLLKKSSTTQHTNNHSYSPSAFTPLASTPRTELDSTVFR

GPRoar2

>XP_021692997.1 octopamine receptor [Aedes aegypti]
METRLDDRCAALLLQLSADDGDYAQQLPEYPSVAANSSTSESHDQQSLLLVPTIATKLSKLLTDLVSVGGGTGGVAGSVNFSALLS
LNFSTSGNSSSIGVSATATTALAVAFDDHLPSSERTQNHSTDGRFNCDGRNLAELATSAFGSATGGGASGDGGFGPKLEVSKTMDV
IGNSGGVGDGTDSVGFGIGDGGAATQLLIANWQDCVVVILFCLLIVVTVIGNTLVILSVITTRRLRTVTNCFVMSLAVADWLVGIF
VMPPAVAVHLLGSWPLGWILCDIWISLDVLLCTASILSLCAISIDRYLAVTQPLNYSRRRRSKRLALLMILVVWVLALAITCPPIL
GWYEPGRRELTQCRYNQNEGYVVFSAMGSFFIPMAVMIYVYVRISCVVASRHDKMTEIEVHKKSHRTRDADEPCYHYASEMDPAQL
SPKRKRSSSQLSTATTTYVTQLGPGGGGGSSSFTALSGDCPGGDCLKLGGGGTRKNHKNGHYELVDINSMSKTTTSFVGNGGGTNG
AGLDEDHMDPGGQRESPGATGGIKRNSTIHYKLASTTELWSSANNVFNQQQKPQTANQSGGLRRTNTISNSITSSTATASASAVAA
GRNIRIHQKSLSSRILSMKRENKTTQTLSIVVGGFIACWLPFFIHYIITPFLPEELAAPKLGEFFTWLGWINSAINPFIYAFYSVD
FRAAFWRLTLRRFFRNSEKAPFANIHNMSMRR

GPRoar4

>XP_021693342.1 octopamine receptor beta-2R [Aedes aegypti]
MMNPSNDDLPKYMYSAAAAAVSSSFSLASSSGSAHSQEGHSSFGGGSNSTGGTSLFFNGSGAALASAAAKAAGSSTIFTVGASAVP
GRAGDPGGPGDPTGNHVIFQGYSTSSPTSLDMTTVGSDAVMDDCCGAAAASGEWLDVLLLVLKASIMMFIIVAAIFGNLLVIISVK
RHRKLRVITNYFVVSLAMADIMVAMMAMTFNFSVQITGSWKFGSFMCDVWNSLDVYFSTASILHLCCISVDRYYAIVKPLKYPISM
TKRVVAIMLLNTWISPALLSFVPIFAGWYTTDKHKEDVLNNPDSCLFIVNKPYAVISSSISFFIPCTIMVFTYFQIFREANRQEKQ
LAMRQGTAMLMHQHNAGGGGVGGTGSGSAGGGRTNGEALSGSGSSRTLTMHEVDAEQTPTKDRHLIKMKREHKAARTLGIIMGTFI
LCWLPFFLWYIITSLCDECPNPDIVVVLVFWVGYFNSTLNPLIYAYFNRDFREAFRNTLDCMFCAWWRRETSPLDINVRRSSLRYD
CRARSVYSENYLRSTTQNDRLNSEIGESL

Unassigned Biogenic Amine Receptors

GPRnna19

>XP_021694244.1 tyramine/octopamine receptor [Aedes aegypti]
MMMVVATIAALNERNGSSLQVIEKYIGGNYNCDVGKIARLAKLSQDIGYVLYSALGSFYIPSCIMVFVYIRIYYAAKARARRGIRK
KPLKPPSEQDTSFTRPANPMPSLPSASSMSAVAVAPASSVNGNGQYATANSNNNNNLSSGPNNPGQHQQQIATIEPPPRGLAGGAA
GGPTQQMSIPTVTCDFASDMSTSEAADHDHAAAVAEMKDTLKVGKPGIGLTSLPLLRGNNGSAASSSGAVISPGLARNRALSVGIE

TDMVSEFDPSSSDSGVISRCAVVKPLKFRLCQPIFGKRVGKGRNKNNNARGNCAGVGPAAMGGQAISISAKQNDSACSELEPALPK
PKPRDPEKEKRRIARKKEKRATLILGLIMGSFIACWLPFFFLYILVPVCKDCHIPDFAFSLAFWLGYMNSALNPAIYTIFNKDFRR
AFRRILFK

Non-synonymous(s) substitution between gene models predicted from the L5 assembly as compared to that predicted from the L3 assembly (aqua shading); amino acid sequence unique to gene model predicted from the L5 assembly (gray shading); amino acids associated with functional G protein-coupled receptors in other species (olive shading).
