# Supplementary material for: Improved reference genome of Aedes aegypti informs arbovirus vector control
Source: Nature. 2018 Nov 14;563(7732):501–7. doi: 10.1038/s41586-018-0692-z (PMC6421076; doi:10.1038/s41586-018-0692-z)
Supplement: Supplementary file 3 — This file contains Supplementary Data 1-24 and a detailed guide for the datasets [file 41586_2018_692_MOESM3_ESM.zip › 41586_2018_692_MOESM3_ESM/Supplementary Data 16 - Gene family annotations - Opsin peptide sequences.pdf]

Matthews et al., Supplementary Data 16

Opsin genes predicted from the *Aedes aegypti* L5 Genome Assembly

Protein sequences

GPRop1

>XP_001651997.1 opsin-1 [Aedes aegypti]
MAAFVAPHFDAWQSSGNMTVVDKVPPEMLHMVHPHWNQFPPMNPLWHSILGFAIFVLGVVSMLGNGCVIYIFTGTRSLRTPSNLLVVNLAFSDFFMMFT
MGPPMVINCWHETWVFGPFACELYAMFGSLFGCASIWTMTMIAFDRYNVIVKGLSAKPLTNNGALLRILGIWAFALFWTLAPFFGWNRYVPEGNMTACG
TDYLTTTWLNRSYIIVYAIFVYWTPLLTIIYSYTFILKAVSAHEKNMREQAKKMNVASLRSSEAQQTSAEIKLAKVALVTISLWFMAWTPYLVINFTGI
FKAAPISPLATIWGSLFAKANAVYNPIVYGISHPKYRAALTQKFPALSCTDAPAASNSDDNQSTVSGATTATDEKA

GPRop2

>XP_001657619.2 opsin-1 [Aedes aegypti]
MAAFVEPHFDAWQAAGGNLTVVDKVPPEMLHMIHPHWNQFPPMNPLWHSILGFAIFVLGMVSMIGNGFVMSIFTSTPSLRTPSNLLVVNLAFSDFLMMF
TMGPPMVINCWHETWVFGPFACEVYACLGSLFGCASIWTMTMIAFDRYNVIVKGLAAKPLTNNGAMLRILGIWAFALFWTLAPFFGWNRYVPEGNMTAC
GTDYLTQTWLSRSYIIIYAIFVYWTPLLTIIYSYTFILKAVSAHEAQMREQAKKMNVASLRSTEANQTSAEIKLAKVALVTISLWFMAWTPYLVINFTG
IFKAAPISPLATIWGSLFAKANAVYNPIVYGISHPKYRAALYQRHPWLSCQDAQESSHDNQSTVSGATTATEEKA

GPRop3

>XP_001651998.1 opsin-1 [Aedes aegypti]
MVALAEPHFQAWIQSAATNVSVVDKVPADMLHMVDAHWYQFPPMNPLWHSLLGFAIFVLCFISLIGNGMVIYIFTNTKTLRTPSNLLVVNLAFSDFLMM
FTMGPPMVYNCYHETWVLGPFACELYGMFGSLFGCVSIWTMTMIAFDRYNVIVKGLSAKPMGNNGALLRIFFVWGSSLAWTLAPFFGWNRYVPEGNMSA
CGTDYLTDTLLSRSYILVYSIFVYFAPLLLIIYSYIFIIKAVSAHEKNMREQAKKMNVASLRSSEAQSTSTEMKLAKVALVTISLWFMAWTPYLIINYT
GIFKAAPITPLATIWGSLFAKANAVYNPIVYGISHPKYRAALYQKFPSLSCTDAADDSQSMASGTTTVVQEEKPSA

GPRop4

>XP_001651166.1 opsin-1 [Aedes aegypti]
MASYGAWMAAQSAGHAVASNLTVVDRVPADMLHMVDAHWYQFPPMNPLWHSLLGFAIAVLCFISVVGNGMVMYIFTNTKTLRTPSNLLVVNLAFSDFLM
MFTMGPPMVINCYYETWVFGPFACEVYGMFGSLFGCVSIWTMTMIAFDRYNVIVNGLSGKPLTNNGALARICGVWVSTLAWTLAPFFGWNRYVPEGNMS
ACGTDYLTDTFSSRSYILVYSIFVYFAPLFLIIYSYTFIIKAVSAHEKNMREQAKKMNVASLRSSEAQNTSTEMKLAKVALVTISLWFLAWTPYLIINY
TGIFKASPISPLATIWGSLFAKANAVYNPIVYGISHPKYRAALYQKFPSLSCTDPADDTQSVASGTTTVVSEKSEKTESA

GPRop5

>XP_001651167.1 opsin-1 [Aedes aegypti]
MASYGAWMAAQSAGHAVASNLTVVDRVPADMLHMVDAHWYQFPPMNPLWHSLLGFAIAVLCFISVVGNGMVMYIFTNTKTLRTPSNLLVVNLAFSDFLM
MFTMGPPMVINCYYETWVFGPFACEVYGMFGSLFGCVSIWTMTMIAFDRYNVIVNGLSGKPLTNNGALARICGVWVSTLAWTLAPFFGWNRYVPEGNMS
ACGTDYLTDTFSSRSYILVYSIFVYFAPLFLIIYSYTFIIKAVSAHEKNMREQAKKMNVASLRSSEAQNTSTEMKLAKVALVTISLWFLAWTPYLIINY
TGIFKASPISPLATIWGSLFAKANAVYNPIVYGISHPKYRAALYQKFPSLSCTDPADDSQSVASGTTTVVSEKSEKTESA

GPRop7

>XP_001652725.2 rhodopsin [Aedes aegypti]
MAYYGPPPNLWGHSVTNLTVVDKVPPEIMHLVDPHWYQFPPMNPLWHSIIGFAIFMLGMISTVGNGVVIYIFSTEKSLRTPSNLFVVNLAVSDFLMMAT
NATTMVYNCWFETWSLGLLMCDLYAFTGSLFGCCSIWSMTMIAMDRYNVIVKGLSGKPLTNTGAIVRICVCWTIGIVWGSMPMLGWNRYVPEGNMTACG
TDYLTDDWFSKSYILCYSFCVYYIPLFTIIYCYVYIVKAVTVHERTMREQAKRMNIQSLRQGDDGKAAEMKLAKIALVTISLWFLAWTPYTIINYTGVF
KMASLTPLATIWGSVFAKCSSVYNPIVYGISHPKYRAALVRRFPALGCGDASGGADAKSMASEVSAVSGGGSAGAGMETTAA

GPRop8

>XP_021698798.1 opsin, ultraviolet-sensitive [Aedes aegypti]
MPFEEHLSDNFTAVLRPEARLSAETRYLGWNVAPEDLPHIPEHWLKYPEPEASLHYLLGLLYIAFTIFALVGNGLVIWVFSSAKSLRTPSNVFVVNLAI
CDFLMMVKTPIFIYNSFTKGFTTGFLGCQVFAFIGSLSGIGAGATNACIAYDRYNTIARPFEGKLTHTKAIFITCMVWTYTFPWAVLPLMELWGRFVPE
GYLTSCTFDYLTNTFDNRMFVGTIFTFSYVLPMSLIIYYYSQIVSHVVNHEKALREQAKKMNVDSLRSNQNQANTSVEVRIAKAAITVCFLFVASWTPY
AVLALIGAFGDKTLLTPGVTMFPACACKFVACLDPYVYAISHPRYRVELQKRLPWLAITESLPSDSASNVTDATTANTSAPASS

GPRop9

```
>XP_001662982.2 opsin-3 [Aedes aegypti]
MFLLNETDAAIFPMARTGDMPKMLGWNLPPEQQHLVHDHWKDFPAPPYYMHLLLAMLYFVLMSVSLIGNGIVVWIFSTSKSLRNGSNMFVVNLAIFDLL
MMCEMPMFLVNSFAGYLVGYETSCAVYAALGSLSGIGGSITNAVIAYDRYRTISNPLDGRLNRVQSGILIFITWLWAMPFTILPVFKIWGRYIPEGFLT
TCSFDYLTDDSDTRVFVGCIFAWAYAIPMVLICYYYGRLFGHVSKHELMLKNQARKMNVESLASNRNEKAQSVEIRIARAAFTIFFLFVCAWTPYAIVA
MIGAYGDRTLLTPFFTMIPAVCCKIVSCLDPWVYAISHPKYRQELERRLPWMGIREPADNVSTTDSKHTVVSESLPVGPNGID
```

GPRop10

```
>XP_021702394.1 opsin Rh4 [Aedes aegypti]
MRKWNAQFKSLRTPANYLVINLAIADFIIMLEAPLFVYNSYHQGPATGNVWCTIYALLGAVGGTVAIVTLTMISIDRYNVVVYPLNPKRSTTRLKVALM
IVFAWIYGLVFSVIPALDIGLSRYTPEGFLTACSFDYLERTWDARLFMFLYFIFAWVVPIIAITFCYIQILRVVIGANSIQSSKNKSKTEVKLAGVVIG
IIGLWFIAWTPYAIVAMMGVFGYESLLSPLGSMVPAILAKTAACIDPYFYAMNHPRYRQELRKMFGLNQQDLGNSQYQTSRYTRNASRMDDSEGGASER
VTIGRQPAADDTSLSVSIDLTETNPNSNH
```

GPRop12

```
>XP_001650802.3 vertebrate ancient opsin [Aedes aegypti]
MMTDISVIHVANISRLLILSTQQPTTTTTTTTTTVTTTTISSSSSGGGGGGSGSGATISAGTGLSMVVNGNGDVGGGSAGGSAIIITSATEVFGTGLPV
GSYERDPVPWPVPELMESWAYVASAVTLFFIGFFGFFLNLFVIALMCKDVQLWTPINIILFNLVCSDFSVSIIGNPFTLTSAISRHWIFGRTVCIAYGF
FMSLLGITSITTLTVLSYERFCLISHPFSSRSLSRRGAVFAILFIWSYSFALTSPPLFGWGAYVNEAANISCSVNWESQTLNATSYIIFLFVFGLVVPL
VVIVYSYTNIVVNMKRNAARVGRINRAEKRVTRMVFVMVLAFMIAWTPYAVFALIEQFGPTDIISPALGVLPALIAKSSICYNPIIYVGMNTQFRAAFN
RVRNNESVDNNTITNQKDITMNTSKEIVECSFDFCRKKRLKIKLQSNAKSKNNNNNSRNQSIADPSSTSNGDDLDQPSPAQTVLNSTVANSGSVASFGP
KKRLRSDFELSVISSGKSILIKSNTFRSNLV
```

Non-synonymous(s) substitution between gene model predicted from the L5 assembly as compared to that predicted from the L3 assembly (aqua shading); amino acid sequence unique to gene model predicted from the L5 assembly (gray shading); amino acids associated with functional opsin receptors in other species (olive shading).
