# Supplementary material for: Improved reference genome of Aedes aegypti informs arbovirus vector control
Source: Nature. 2018 Nov 14;563(7732):501–7. doi: 10.1038/s41586-018-0692-z (PMC6421076; doi:10.1038/s41586-018-0692-z)
Supplement: Supplementary file 3 — This file contains Supplementary Data 1-24 and a detailed guide for the datasets [file 41586_2018_692_MOESM3_ESM.zip › 41586_2018_692_MOESM3_ESM/Supplementary Data 2 - Fig 1 - TE - table of TE and repeat content.pdf]

Matthews et al. Supplementary Data 2

TE family	% of the genome (TFfam only)	% of the genome (TEfam and Repbase)	% of the genome *Nene et al.* 2007
Class I			
LTR retrotransposons	12.69	11.67	10.51
Ty1_copia	5.31	4.51	4.31
Ty3_gypsy	2.76	3.5	2.48
Pao_Bel	4.62	3.65	3.72
**ERV**	**NA**	**0.01**	**NA**
Non-LTR retrotransposons	13.5	16.09	14.37
CR1	1	1.33	1.05
I	0.76	2.85	0.67
Jockey	3.55	5.02	3.75
L1	0.32	0.5	0.38
L2	0.2	0.07	0.19
LOA	1.11	0.44	1.29
Loner	0.97	0.46	0.94
Outcast	0.03	0.06	0.08
R1	1.91	1.22	1.97
R4	0.05	0	0.05
RTE	3.6	3.92	4
**R2**	**NA**	**0.05**	**NA**
**Kiri**	**NA**	**0.17**	**NA**
SINEs			
tRNA-related SINE	1	1.16	1.86
Class II			
DNA transposon	5.51	15.06	3.04
DD41D	0	0	0
hAT	0.53	0.45	0.32
ITmD37D	0.1	0.03	0.06
ITmD37E	0.33	0.29	0.34
mariner	0	0	0
P	0.16	0.13	0.15
PIF	3.3	2.85	1.19
piggyBac	0.02	0.01	0.02
pogo	0.73	0.41	0.72
Tc1	0.34	0.57	0.24

**Sola**	**NA**	**2.64**	**NA**
**Transib**	**NA**	**0.41**	**NA**
**Chapaev**	**NA**	**1.03**	**NA**
**CACTA**	**NA**	**0.6**	**NA**
**Cryoton**	**NA**	**0.05**	**NA**
**Shinagawa**	**NA**	**1.29**	**NA**
**Zator**	**NA**	**0.31**	**NA**
**Ginger2/TDD**	**NA**	**0.01**	**NA**
**Kolobok**	**NA**	**0.12**	**NA**
**MuDR**	**NA**	**0.04**	**NA**
**PONY_AA**	**NA**	**0.17**	**NA**
**other DNA transposon**	**NA**	**3.65**	**NA**
MITEs	13.08	9.28	15.79
m3bp	0.48	0.36	0.7
m4bp	2	0.68	3.19
m8bp	1.51	1.39	1.69
m9bp	0.08	0.04	0.15
mTA	6.1	5	6.94
otherMITEs	2.86	1.78	3.12
**m7bp**	**0.05**	**0.03**	**NA**
Helitron	1.31	1.14	1.04
Penelope			
Penelope-like	0.42	0.45	0.44
Class I	27.19	28.92	26.74
Class II	19.9	25.48	19.87
Penelope	0.42	0.45	0.44
Total	47.51	54.85	47.07

Rows in bold indicate new TE types compared to Nene et al. 2007.
