# Supplementary material for: Improved reference genome of Aedes aegypti informs arbovirus vector control
Source: Nature. 2018 Nov 14;563(7732):501–7. doi: 10.1038/s41586-018-0692-z (PMC6421076; doi:10.1038/s41586-018-0692-z)
Supplement: Supplementary file 3 — This file contains Supplementary Data 1-24 and a detailed guide for the datasets [file 41586_2018_692_MOESM3_ESM.zip › 41586_2018_692_MOESM3_ESM/Supplementary Data 24 - Ext Fig 10d - LGIC sequences and notes.pdf]

# Matthews et al., Supplementary Data File 24

<table>
<tr><th>NCBI Accession Number</th><th>Given Name</th><th>Desired Name</th></tr>
<tr><td>XM_021851259</td><td>Alpha-like 1</td><td>Alpha 1 nicotinic acetylcholine receptor subunit</td></tr>
<tr><td colspan="3">>Aaeg_a1<br>MGSVLLTAVFIALHFATGLANPDSKRLYDDLLSNYNRLIRPVGNNSDRLTVKMGLRLSQLIDVNLKNQIM<br>TTNVWVEQEWNDYKLKWNPDDYGGVDQLHVPSEHIWLPDIVLYNNADGNYEVTIMTKAILHHTGKVVWKP<br>PAIYKSFCEIDVEYFPFDEQTCFMKFGSWTYDGYLVDLRHLQQTPDSDNIDVGIDLQDYYISVEWDIMKV<br>PAVRNEKFYSCCEEPYPDIIFNITLRRKTLFYTVNLIIPCVGISFLSVLVFYLPSDSGEKISLCISILLS<br>LTVFFLLLAEIIPPTSLTVPLLGKYLLFTMMLVTLSVVVTIAVLNVNFRSPVTHKMAPWVHRVFIELLPR<br>VLCIERPKKDDEPSENEEQAPEVLTDVFQVPPDVEKYVSYCGKEYTTDFDIPALPPSRFDVAASGGVGPC<br>FGEPPLPALPLPGGDDDLFSPTSNGDMSPNCCGDVSPTFEKPLLREMEKTIEASRFVAQHVRNKDKFESI<br>EEDWKYVALVLDRLFLWIFTIACVMGTALIILQAPSLYDNTQPIDAMYSKIAKKKMELLKMGSENV</td></tr>
<tr><td colspan="3"><u>NOTE</u>: AaegL5 has clarified some discrepancies in the sequence.</td></tr>
<tr><td>XM_001648229</td><td>Alpha-like 2</td><td>Alpha 2 nicotinic acetylcholine receptor subunit</td></tr>
<tr><td colspan="3">>Aaeg-a2<br>MGSVLLTAVFIALHFATGLANPDSKRLYDDLLSNYNRLIRPVGNNSDRLTVKMGLRLSQLIDVNLKNQIM<br>TTNVWVEQEWNDYKLKWNPDDYGGVDQLHVPSEHIWLPDIVLYNNADGNYEVTIMTKAILHHTGKVVWKP<br>PAIYKSFCEIDVEYFPFDEQTCFMKFGSWTYDGYLVDLRHLQQTPDSDNIDVGIDLQDYYISVEWDIMKV<br>PAVRNEKFYSCCEEPYPDIIFNITLRRKTLFYTVNLIIPCVGISFLSVLVFYLPSDSGEKISLCISILLS<br>LTVFFLLLAEIIPPTSLTVPLLGKYLLFTMMLVTLSVVVTIAVLNVNFRSPVTHKMAPWVHRVFIELLPR<br>VLCIERPKKDDEPSENEEQAPEVLTDVFQVPPDVEKYVSYCGKEYTTDFDIPALPPSRFDVAASGGVGPC<br>FGEPPLPALPLPGGDDDLFSPTSNGDMSPNCCGDVSPTFEKPLLREMEKTIEASRFVAQHVRNKDKFESI<br>EEDWKYVALVLDRLFLWIFTIACVMGTALIILQAPSLYDNTQPIDAMYSKIAKKKMELLKMGSENV</td></tr>
<tr><td colspan="3"><u>NOTE</u>: AaegL5 has identified the N-terminal signal peptide thus completing the subunit sequence.</td></tr>
<tr><td>XM_021848318</td><td>Alpha-like (LOC5564067)</td><td>Alpha 3 nicotinic acetylcholine receptor subunit</td></tr>
<tr><td colspan="3">>Aaeg_a3<br>MRLLHGVSFIIFAAVSVCAGNPDAKRLYDDLLSNYNKLVRPVVNVTDALTVKIKLKLSQLIDVNLKNQIM<br>TTNLWVEQTWYDYKLKWEPKEYGGVEMLHVPSDHIWRPDIVLYNNADGNFEVTLATKATLNYTGRVEWRP<br>PAIYKSSCEIDVEYFPFDEQTCVMKFGSWTYDGFQVDLRHIDEVNDTNVVEVGVDLSEFYTSVEWDILEV<br>PAVRNEKFYTCCDEPYLDITFNITMRRKTLFYTVNLIIPCMGISFLTILVFYLPSDSGEKVSLSISILLS<br>LTVFFLLLAEIIPPTSLVVPLLGKFVLFTMILDTFSICVTVIVLNIHFRSPQTHTMAPWVRTIFINHLPK<br>LLVMRRPIYQPLHHFSAASQRFMLRSCTGMGDNIPPLPPTIVFDPSVLLDHHLLDSSDSINTCRLHGSPT<br>HHHHHHGGGHHHHHHGLRGIDLMDDLPLPYQDHNHHNSPMSPINLNLLSSTSSTAVPIPATGGLSSAG<br>AALLDPHSTFQTSLAGGMVGGSSKTSTNVIINTATNTTTTRSNLSCCNNLFLNDGRTGLDSVQLPSSNIV<br>GGVADSSTAVPTVPIINTTDSGLGGSGGGGSGGGTDGKDKSTRHRWLTCPELHKAMDGVTYIADHTRKEE<br>ESTRVKEDWKFVAMVLDRLFLWIFTIAVVFGTAGIILQAPTLYDTRVPIDIKMSEIATTTAKPYIAKPVL</td></tr>
<tr><td colspan="3"><u>NOTE:</u> AaegL5 has clarified some discrepancies in the sequence.</td></tr>
</table>

<table>
<tr><td>XM_021838743</td><td>Alpha-like(LOC5575838), transcript variant X5</td><td>Alpha 4 nicotinic acetylcholine receptor subunit</td></tr>
<tr><td colspan="3">>Aaeg_a4<br>MKKMSPSAGETLRAWLLSALVVHGAVAGNPDAKRLYDDLLSNYNKLVRPVVNTSDVLRVCIKLKLSQLID<br>VNLKNQIMTTNLWVEQSWYDYKLRWEPKEYGGVQMLHVPSDHIWRPDIVLYNNADGHYEVTLMTKATVYN<br>TGLVIWQPPAVYKSSCSIDVEYFPYDVQTCVLKLGSWTYDGFKVDLRHMDEQSGSNIVDVGVDLSEFYMS<br>VEWDILEVPAVRNEKFYTCCDEPYLDITFNITMRRKTLFYTVNIIIPCMGISFLTVLTFYLPSDSGEKVT<br>LSISILISLHVFFLLVVEIIPPTSLVVPLLGKYLIFAMILVSISICVTVVVLNVHFRSPQTHRMAPWVKT<br>FFIDFLPRFLFMKRPAYIENHRKLLPKQPKVCFYPYYSTTALNRVKARFHIRTSSKDDQSPLSLSGTGPF<br>GGSCQIHGPLPVPHSESEELSLSTAADTAVPSGIKSPVFKQPSFSHSSCPIEVHRSCFCVRFIAEHTKML<br>EDSTKVKEDWKYVAMVLDRLFLWIFTLAVLAGTAGIILQAPTLYDDRIPIDKTFDELATSTVVRCPPQ</td></tr>
<tr><td colspan="3"><u>NOTE</u>: AaegL5 has identified the N-terminal signal peptide thus completing the subunit sequence.</td></tr>
<tr><td>XM_021847824</td><td>alpha-7 (LOC5567018), transcript variant X2</td><td>Alpha 5 nicotinic acetylcholine receptor subunit</td></tr>
<tr><td colspan="3">>Aaeg_a5<br>MLSRYHNFRCYKQLRLYKWIIVLMLIVNSVMVCKAGYNEKRLLHDLLDTYNTLERPVVNESDPLQLSFGL<br>TLMQIIDVDEKNQLLVTNIWLKLEWNDMNLRWNISDYGGVKDLRIPPHRIWKPDVLMYNSADEGFDGTYP<br>TNVVVRNNGSCLYVPPGIFKSTCKIDITWFPFDDQRCEMKFGSWTYDGFQLDLQLQDEAGGDISSFVTNG<br>EWDLLGVPGKRNEIYYNCCPEPYIDITFAIIIRRRTLYYFFNLIVPCVLIASMALLGFTLPPDSGEKLSL<br>GVTILLSLTVFLNMVAETMPATSDAVPLLGTYFNCIMFMVASSVVSTILILNYHHRNADTHEMSDWVRII<br>FLYWLPCILRMSRPGRSLSVEYPPTPTSDSSERKTHIQDVELKERSSKSLLANVLDIDDDFRHNCRPLTP<br>GGTLPHNPTYFRTVYSDDGSMGPIGSNRMPDAIAPPHTCFTSSADYELALILKEIRFITDQIRKEDEDSD<br>VAKDWKFAAMVVDRLCLIIFTFFTIVATIAVLFSAPHIIVS</td></tr>
<tr><td colspan="3"><u>NOTE:</u> AaegL5 has updated the alpha 5 by identifying a possible N-terminal sequence, however this does not include a putative N-terminal signal peptide so it remains unclear whether this sequence contains the correct N-terminus.</td></tr>
<tr><td>XM_001658813</td><td>alpha-7 (LOC5569979), transcript variant X6</td><td>Alpha 6 nicotinic acetylcholine receptor subunit</td></tr>
<tr><td colspan="3">>Aaeg_a6<br>MYFVMDLYLIVLCLLVICIRDSLQGPHEKNLLNKLLNTYNTLERPVANESESLEVKFGLTLQQIIDVDEK<br>NQILTTNAWLNLDEKNQLLITNIWLSLEWNDYSLRWNESEYGGVKDLRITPNKLWKPDVLMYNSADEGFD<br>GTYHTNVVVKNNGSCLYVPPGIFKSTCKIDITWFPFDDQHCEMKFGSWTYDGNQLDLVLNSDEGGDLSDF<br>ITNGEWYLIGMPGKKNTITYQCCPEPYVDITFTIQIRRRTLYYFFNLIVPCVLISSMALLGFTLPPDSGE<br>KLTLGVTILLSLTVFLNLVAETLPQVSDAIPLLGTYFNCIMFMVASSVVLTVVVLNYHHRTADIHEMPPW<br>IKSVFLQWLPWILRMGRPGKKITRKTILLSNRMKELELKERSSKSLLANVLDIEDDFRHASAGITGSTTA<br>ISTSGFSRPTTVEEHHAMSGCSHKDLHYILKELQFITNRMRKADEEAELISDWKFAAMVVDRFCLFVFTL<br>FTIIATVTVLLSAPHIIVQ</td></tr>
<tr><td colspan="3"><u>NOTE:</u> AaegL5 has identified the N-terminal signal peptide thus completing the subunit sequence.</td></tr>
</table>

<table>
<tr><td>XM_021846964</td><td>alpha-7 (LOC5569480)</td><td>Alpha 7 nicotinic acetylcholine receptor subunit</td></tr>
<tr><td colspan="3">>Aaeg_a7<br>MLMPPSILVLGIGWMWMPLQVVNAGYHEKRLLHNLLDNYNVLERPVVNESDPLQLSFGLTLMQIIDVDEK<br>NQLLITNIWLKLEWNDMNVRWNSSEYGGVRDLRIPPHRLWKPDVLMYNSADEGFDGTYPTNVVVRNNGSC<br>LYVPPGIFKSTCKIDITWFPFDDQRCEMKFGSWTYDGFQLDLQLQDEAGGDISSFITNGEWELLGVPGKR<br>NEIYYNCCPEPYIDITFAILIRRKTLYYFFNLIVPCVLIASMALLGFTLPPDSGEKLSLGVTILLSLTVF<br>LNMVAETMPATSDAVPLLGTYFNCIMFMVASSVVSTILILNYHHRNADTHVMSDWIRVVFLSWLPCILRM<br>SRPGEPFPHPCRQSVDEKNKQLQEVEMRERSSKSLLANVLDIDDDFRCNHRCSTLPHNPTYYRTMYRQGD<br>ADGGVGPLGTGTDTIGRSAGGLGTISAAGGGRLHETVSSHTCLSSSAEYELALILKELRYITDTLRKEDE<br>SGDVTRDWKFAAMVVDRLCLIIFTLFTIIATLAVLFSAPHFLVS</td></tr>
<tr><td colspan="3"><u>NOTE:</u> AaegL5 has clarified some discrepancies in the sequence.</td></tr>
<tr><td>XM_021852574</td><td>beta-like 2(LOC5563989)</td><td>Alpha 8 nicotinic acetylcholine receptor subunit</td></tr>
<tr><td colspan="3">>Aaeg_a8<br>MGSFIKFINFKLCLLSMIALSYAQASIVHIEANPDAKRLYDDLLSNYNRLIRPVVNNTETLTVWLGLKLS<br>QLIEVSLRNQVMTTNLWVKQKWFDYKLKWDPEEYGGVEMLYVPSEQIWLPDIVLYNNWDGNYEVTLMTKA<br>TLKYTGEVFWEPPAIYKSSCEMNVEYFPYDEQTCLMKFGSWTYNGAQVELRHLDQVPGSNLVQIGIDLSE<br>FYLSVEWDILEVPASRNEEYYPCCPEPFSDITFKLTMRRKTLFYTVNLIIPCVGITFLTVLVFYLPSDSG<br>EKVTLCISILVSLTVFFLLLAEIIPPTSLAVPLLGKYLLFTMILVSLSVWTTVCVLNVWYRSTSTHKMSP<br>IVKRLFLEIMPKIMMMRRAKYTLPDYDDSTPSNGYTNEIDMSISDYPGEFKEGGDSFDNIGMNLPHGSVE<br>NDNVIPKQLSPEVLSAIQAVRFIAQHIKDADKDNEIVEDWKFVSMVLDRFFLWVFTISCIFGTFGIICQS<br>PSLYDTRAPVDQQLSEIPLRKNNFMLPPDIVRITLD</td></tr>
<tr><td colspan="3"><u>NOTE:</u> AaegL5 has clarified some discrepancies in the sequence.</td></tr>
<tr><td>XM_001651961</td><td>alpha-like 1(LOC5568075)</td><td>Alpha 9 nicotinic acetylcholine receptor subunit</td></tr>
<tr><td colspan="3">>Aaeg_a9<br>MRVSLLIVCLIVHCYADDASSNSIKPSGSQTWIDKLKNDLFVNYDRNLRPAEYYNVTNLDIGLTIWHVDI<br>DEEKSILSLYGWVKMTWNDDKLKWNPSDYGNVEQFRYTPENVWKPDVVLYNNARGADNLHYGNTNVIIYN<br>SGKVLWVPPTDFHSFCELNLRFWPFDYQRCFLKIGSWTYDELHLNMTTTEVNPEILWLVPNHKWSIRKVT<br>VERHVKKYECCKEPYVDIQYNVTLQRHSATHKAIVVSPAFVIMLMALSVFWLPPQCGEKIVLNGIIALII<br>TIFLIYFAQQLPAMSGNPPLIVTFYSTTFYLVAISTILSVIALRMTRNKHCRAVPRPLKGQLDGCLGSVL<br>GVGNTSQLDDEKETVGEEVSVNSKQRDWCRLATLLDRLAFVVYLIVFVASIIYFTL</td></tr>
<tr><td colspan="3"><u>NOTE:</u> AaegL5 has clarified some discrepancies in the sequence.</td></tr>
<tr><td>XM_001651960</td><td>alpha-like1(LOC5568073), transcript variant X2</td><td>Alpha 10 nicotinic acetylcholine receptor subunit</td></tr>
<tr><td colspan="3">>Aaeg_a10<br>MGKGHRLAFLLVCLAGCCYADDATSDSNKPATTQTWIDKLKKDLLANYDRNVRPTQHYNVTHLDLKMTIR<br>HVDIDEENSIFSVYGWVKMTWTDDKLKWKPADYGNVDLFRCNPDSVWKPDVVLYNNARGSDNLHYGQTNV<br>IVYSSGQVLWVPPTDYHSFCELNLRYWPFDYQTCILKVGSWTYDGYKLNLTTSEAEPEIDIGVPNNEWSI<br>RKVTTDRNTVYYKCCSEPYIDIQYNVTLQRHSSTHKAIVVSPAFVIMLLALSVFWLPPHCGEKIVLNGII<br>VLVVTVFLIYFAQQLPAMSGNTPLIVTFYSTTFYLVAISTILSVIILRITRGKRCHAVPRVLKSQLDGCL<br>GSVLRVGNVGPSEEDKEAAGGEISVNSKQQDWCRLAVLLDRLAFVVYLIVFAISIICFSL</td></tr>
<tr><td colspan="3"><u>NOTE:</u> First identified in the AaegL5 assembly.</td></tr>
</table>

<table>
<tr><td>XM_001660871</td><td>beta-like 1(LOC5573524)</td><td>Beta 1 nicotinic acetylcholine receptor subunit</td></tr>
<tr><td colspan="3">>Aaeg_b1<br>MRFKQFLLSLTIAIQYYFGIANGSEDEERLVRDLFRGYNKLIRPVQNMTQKVDVRFGLAFVQLINVNEKN<br>QIMKSNVWLRLVWSDYQLQWDEADYGGIGVLRLPPDKVWKPDIVLFNNADGNYEVRYKSNVLIYPNGEVL<br>WVPPAIYQSSCTIDVTYFPFDQQTCIMKFGSWTFNGDQVSLALYNNKNFVDLSDYWKSGTWDIIEVPAYL<br>NVYEGNPTETDITFYIIIRRKTLFYTVNLILPTVLISFLCVLVFYLPAEAGEKVTLGISILLSLVVFLLL<br>VSKILPPTSLVLPLIAKYLLFTFIMNTVSILVTVIIINWNFRGPRTHRMPMWIRSVFLHYLPAMLLMKRP<br>RKTRLRWMMEMPGMSMPPQPHTHPSYGSPAEVPKHISALGGKQSKMDVMELSDLHHPNCKMNRKMNSGDI<br>GLGDSCRRESESSDSILLSPEASKATEAVEFIAEHLRNEDLYIQTREDWKYVAMVIDRLQLYIFFIVTTA<br>GTVGILMDAPHIFEYVDQDRIIEIYRGK</td></tr>
<tr><td colspan="3"><u>NOTE:</u> AaegL5 has clarified some discrepancies in the sequence.</td></tr>
<tr><td>XM_011494733</td><td>alpha-like 1(LOC5568073)</td><td>Beta 2 nicotinic acetylcholine receptor subunit</td></tr>
<tr><td colspan="3">>Aaeg_b2<br>MGKGHRLAFLLVCLAGCCYADDATSDSNKPATTQTWIDKLKKDLLANYDRNVRPTQHYNVTHLDLKMTIR<br>HVDIDEENSIFSVYGWVKMGWMDTRLTWSPEEYGGLRSVVFHSYYLWDPEINMHSVTLESSGSAFIGTAN<br>VRVSFDGMHNCTERLNFKSFCEMNFRRWPFDTQHCSVVLGKTTDDDMLQITTLPAEWEKNDQINPMWQIV<br>GVTVEKYQNPMEAQYEKYNGYQYGVVVRRKVQIFNSTIIAPAIVLILMSLASFWMPAYSSEKVLLNCINA<br>AVVCAFLLFFTIHLPLLATRTPLIVMFFSNSLYLTALSLILSVTVVNIVKSKHSKPLHPYIKGFISLPGV<br>SMFAWIGTNQRKTTTDEEDWSGEMKNDSLTEESCSSADEASQVQLGIQQDWIQFAVILERICFVIYVFLY<br>SVMAASYLH</td></tr>
<tr><td colspan="3"><u>NOTE:</u> First identified in the AaegL5 assembly.</td></tr>
<tr><td>XM_001650001</td><td>beta-3 (LOC5565694)</td><td>Beta 3 nicotinic acetylcholine receptor subunit</td></tr>
<tr><td colspan="3">>Aaeg_b3<br>MKLFIILLAMQNINEIVSLVNCGLEPSAPDAILRKKLLCGQYDNSERPVKKYSDPVIVSMHLVLQNFDID<br>DDRQKLFINVWIRLSWKDQFLTWDPREHNGLKDLMVDSKDIWLPDMMPYAAYYSNNLDVSCTSPKCSVLS<br>DGEVRCIPACDYHSLCETDFRNWPFDRMNCTIRFGMWAEYSNEVDFTADGMSFISNQTNSHNEWLIVATN<br>FTKHETDTTDGNDTTKYPSVVYNFVLERHSGVHCAIILTPAFVMISLNLISLWINFCTIERLIMLSISVF<br>IHFLFIVNMYWQVPYNGSTVPVFLIFFRDSLIITASLLILTVFIKHLYLSGKKVPMVLSSTVAVLTGNQL<br>GKWFFNLESTTGLEVEPLEENRPNEESPDESTRDTAILVGEQTDGNADKPSKLGDEMKVFAMVLDRLLFI<br>VGLVSYTLMIVTLIPRN</td></tr>
<tr><td colspan="3"><u>NOTE:</u> First identified in the AaegL5 assembly.</td></tr>
<tr><td>XM_021844896</td><td>alpha-4 (LOC5565695), transcript variant X2</td><td>Beta 4 nicotinic acetylcholine receptor subunit</td></tr>
<tr><td colspan="3">>Aaeg_b4<br>MSSRVNRLVAVGVSILVIVSSKGVFSGPAAPFDCASEVNGNTTTEEALKNALLCGTGYNTHQRPVKNQQD<br>RVAMYIGADVMNVELIHNSFAKLEITVEISMQWYDAYLHWNKKAQNNIQTLSVSEKDIWTPILSAKSLSK<br>SPVQKVDHCYQVKCHLSSDGEVMYSMLCTFTVDCLDNSLHWAFETKDCPLRIFTPEYDINQLSLFHFQRR<br>LSYSVAGVLPYKITSFQMAIVNNSVSPEFRMDIVVERMVGPHLVVFFILILILMTLNLMITWFRIDTTVR<br>AVTSITSLALHAIYTVILYWYANTKMHPASCLANILLGSLIITIILIGVLVYSMNITKQSSMGVPHALQK<br>CYQSVTKIAILKAFLKLGYLNINDQLIKPSKKSSSAGFSNHVDHIESTSKDTAIIEQTEDSEEGDVDPTK<br>LKWNVLIQPFDRIIFCGIAIAYALMLLVLFLA</td></tr>
<tr><td colspan="3"><u>NOTE:</u> First identified in the AaegL5 assembly.</td></tr>
</table>

<table>
<tr><td>XM_001649283</td><td>glycine receptor subunit alpha-2(LOC5564979)</td><td>CLGC1 (cys-loop ligand-gated ion channel subunit in Insect Group 1)</td></tr>
<tr><td colspan="3">>Aaeg_CLGC1<br>MRPMIHISAIVSVLFIAVEIFAEPPNDITTEASKAPTRANLANETEYVDTVQLIKEYQNTNPADNDTVTT<br>ISSTSTETLSTSSTSTPTENVTESSTAASTENKTMTPEEIQRLLLPPAKVEASILHLNASDEVHEKIIKE<br>SDCPSLDEAHKLSQTQLLQRLTHGCRYDRLERPVEYADNGTKLPVQVYARAYIYFLQNLEAHDLQFKIHA<br>LLQLRYVDSRLVFKKVAPNRTEPIMGEQSLRDVLWVPHVFLANERSSDILGTAEKDILTSVSPDGTVIIS<br>TRISATLYCWMNLQKFPFDEQHCSTVLESWMYNEADLNLQWEKKSPVTLAPELHLTEYVLLDMFTNETMI<br>NADLSDLRHGAFAGNYSSLSFTVHLAREMGFYLMDYFIPSIMLVAISWVTFWLQADQSAPRITLGTSTML<br>TFITLASAQGKTLPKVSYIKASEIWFLGCTGFIFGSLVEFAFVNTIWRRQKNVELKKKNSKYILKSTFTP<br>VPSRKNTSGNLQKSHSCTSLDAQSTITASNNSYNNYLTVHAFPSKTNSLLPIITTSNADSPDSRNGNVSI<br>KIEDTSSSTNDLNKTNEQSNVNGNGWTTMTPQEIAIWIDKRSRFVFPVCFFIFNIFYWSFVYYL</td></tr>
<tr><td colspan="3"><u>NOTE:</u> AaegL5 has clarified some discrepancies in the sequence.</td></tr>
<tr><td>XM_021848575</td><td>glutamate-gated chloride channel(LOC5580270), transcript variant X4</td><td>GluCl (glutamate-gated chloride channel)</td></tr>
<tr><td colspan="3">>Aaeg_GluCl<br>MAPGHYFWAIFYFACLCSASLANNAKVNFREKEKKILDQILGAGKYDARIRPSGINGTDGPAIVRINLFV<br>RSIMTISDIKMEYSVQLTFREQWLDERLKFDDIGGRLKYLTLTEANRVWMPDLFFSNEKEGHFHNIIMPN<br>VYIRIFPYGSVLYSIRISLTLACPMNLKLYPLDRQVCSLRMASYGWTTADLVFLWKEGDPVQVVKNLHLP<br>RFTLEKFLTDYCNSKTNTGKYYSWPWRYLPKSNVLLPILGEYSCLKVDLLFKREFSYYLIQIYIPCCMLV<br>IVSWVSFWLDQGAVPARVSLGVTTLLTMATQTSGINASLPPVSYTKAIDVWTGVCLTFVFGALLEFALVN<br>YASRSADRAADIQRENMKKKRREMEQVSLDAASDLLDTDSNATFAMKPLVRHPGDPMAMEKLRQCEVHMQ<br>APKRPNCCRTWWSRFPTRQCSRSKRIDVISRITFPLVFALFNLVYWSTYLFREEEED</td></tr>
<tr><td colspan="3"><u>NOTE:</u> AaegL5 has clarified some discrepancies in the sequence.</td></tr>
<tr><td>XM_021852040</td><td>gamma-aminobutyric acid receptor alpha-like (LOC110678752), transcript variant X1</td><td>GRD (GABA and glycine-like receptor of Drosophila)</td></tr>
<tr><td colspan="3">>Aaeg_GRD<br>MKANLFIAINRIIFSSFSSAVFGIGTSFFWAATPDPATVAVVGAIRLTQRNNHGNISELLDNLLRGYDNS<br>IRPDFGGPPAVIEVDIMVRSMGPISEVDMTYSMDCYFRQSWVDRRLEFTGEYNTLALSISMLGRIWKPDT<br>YFYNGKQSYLHTITTPNKFVRINQDGRVLYSSRLTIKAGCPMNLEDFPMDIQRCPLKFGSFGYTSNDVLY<br>RWNSGRSAVAIAEDMKLSQFDLVDCPAGNVTDRVVHSTASVGATVINAANDIDGLDPNAKLYVSEYSMLL<br>VSFHLQRHMGNFLIQVYGPCVLLVVLSWVSFWLNREATADRVSLGITTVLTMTFLGLEARTDLPKVPYPT<br>ALDFFVFLSFAFIFATIIQFAVVHYFTKYGSGECYFSADELTSSSDEDSDCGGGNANGDHSHLHSIKISS<br>KRCSAVTSISGAGGTDSKVIEVIPLSVCSIPVTPVRKSSKTSWADLSCFGNKNEDIPPSFASSSFSTPVP<br>STKRSSLQLSLGGSAQIQRRRKRKKRTPRFNSVSKIDRASRIFFPLLFLAINVFYWFLYLSRSERLPQQH<br>KN</td></tr>
<tr><td colspan="3"><u>NOTE:</u> AaegL5 has updated the GRD by identifying a possible N-terminal sequence, however this does not include a putative N-terminal signal peptide so it remains unclear whether this sequence contains the correct N-terminus.</td></tr>
</table>

<table>
<tr><td>XM_021852483</td><td>glycine receptor subunit alpha-3 (LOC5576018)</td><td>HisCl1 (histamine-gated chloride channel 1)</td></tr>
<tr><td colspan="3">>Aaeg_HisCl1<br>MLLDYGLSLLLTLLTVCITGIAGESYQTSLTFNDILPEDPKLYDKMRPPKKDGQPTTVLFHVTVMGLDSI<br>DETSMTYAADIFFAQTWKDHRLRLPENMTSEYRLLEVEWLKNMWRPDSFFKNAKSVTFQTMTIPNHYMWL<br>YKDKTILYMVKLTLRLSCAMNFLIYPHDTQECKLQMESLSHTTDDMIFQWDPEVPLVVDEHIELPQLALV<br>KNNTADCTQVYSTGNFTCLEVVFVLKRRLGYYLFHTYIPTCLIVIMSWVSFWIKPEAAPARVTLGVTSLL<br>TLSTQHAKSQASLPPVSYLKAVDAFMSVCTVFVFMALMEYCLVNIVLGDSDPPNPKPHPPPKMDRFFDFN<br>APGGKNGKRNSKSLENSETTTVNTFQRQESTLLLSPVPHIQTIPPPPCKPASAIPKLTPAQIRLRRAINI<br>DRFSRVFFPLLFTLLNTAYWIMFYEYI</td></tr>
<tr><td colspan="3"><u>NOTE:</u> AaegL5 has clarified some discrepancies in the sequence.</td></tr>
<tr><td>Not present in AaegL5.</td><td></td><td>HisCl2 (histamine-gated chloride channel 2)</td></tr>
<tr><td colspan="3">>Aaeg_HisCl2<br>MTSKWTLLLIPLMTSCFHSTLGAKTNRNNYANISDIGELSKHHAVSLSLSDILPQHHKTYDKNRAPKLLG<br>QPTVVYFHVTVLSIDSINEESMTYVADIFLAQSWRDPRLRLPENMSEEYRILDVDWLHNIWRPDCFFKNA<br>KKVTFHEMSIPNHYLWLYHDKTLLYMSKLTLVLSCAMKFESYPHDTQVCSMMIESVSHTVQDLVFIWNMT<br>DPLVVNPEIELPQLDISNNYTTDCTIEYSTGNFTCLAVVFNLRRRLGYHLFHTYIPSALIVVMSWISFWI<br>KPEAIPARVTLGVTSLLTLATQNTQSQQSLPPVSYVKAIDVWMSSCSVFVFLSLMEFAVVNNYMGPVATK<br>AMKGYSDEDLSEAIDFNKNGFNKTHRSSDLPQYDTFCNGRETALCIDKFSRFFFPFSFFILNVTYWTTFL</td></tr>
<tr><td colspan="3"><u>NOTE:</u> HisCl2 has been identified in Aedes Aegypti L3 and Aedes Albopticus genomes. The AaegL5 data does not seem to contain this and hence would be appropriate if this sequence was included.</td></tr>
<tr><td>XM_021839245</td><td>gamma-aminobutyric acid receptor subunit beta-like (LOC5573727), transcript variant X2</td><td>LCCH3 (ligand-gated chloride channel homolog 3)</td></tr>
<tr><td colspan="3">>Aaeg_LCCH3<br>MWWQLMGFALLHAFQVGLAVKGGHSKSMAAGRLENVTQTISRILEGYDIRLRPNFGGDPLHVGMDLTIAS<br>FDAISEVNMDYTITMYLNQYWKDERLAFNARQYDQNGDIMIEDDGANDVITLSGDFAEKIWVPDTFFAND<br>KNSFLHDVTERNKLVRLAGDGSVTYGMRFTTTLACMMDLHYYPLDSQNCTVEIESYGYTVSDVLMYWRST<br>PIRGVEEAELPQFTIIGYETNDRKERLATGEYQRLSLSFKLQRNIGYFVFQTYLPSILIVMLSWVSFWIN<br>HEATSARVALGITTVLTMTTISTGVRSSLPRISYVKAIDIYLVMCFVFVFAALLEYAAVNYTYWGARAKK<br>KSKKNKEAEKKVSRKQENSAGCSSEDIIELQDVRMSPIASLRNRHYANTTSSSGADAVDLAKFPPSFRIA<br>RPYGSSTRSSGLRYRGNRGNNRPKMLHAIKRGASVIKASIPKIKDVNVIDKYSRVIFPVSFAAFNAGYWI<br>FYVLE</td></tr>
<tr><td colspan="3"><u>NOTE:</u> LCCH3 in AaegL5 data shows to have some extra sequences which does not look appropriate based on homology with LCCH3 from other species and was thus removed. This sequence contains a putative N-terminal signal peptide.</td></tr>
</table>

<table>
<tr><td>XM_021842823</td><td>glutamate-gated chloride channel(LOC5579421), transcript variant X20</td><td>pHCL (pH-sensitive chloride channel)</td></tr>
<tr><td colspan="3">>Aaeg_pHCL<br>MGFTCEKAHYRTSHSEQSWIETIGCDQLTVPELKPVGSVSNFKKHQKLRPKAHNQSKSPPAGEHQSTTSI<br>LPPATEPPTGVNSGISLHYPFNLFHPFHLSSYQCSAIILDKLSQFISKYVVVRSGSGWSVAQPCVSIGSG<br>PFPSVPPEPSASKYQPFPPSSPSFPRSTTSRVAISRASCTSTAEPQQKVVPLPPLSSCDPLVAPVHPIGS<br>TASTTGCTKTLPYSSSLSLEQAPSAAASLSSTTSQALLLTPSSSSLRLPSRDPSSHETPTISESSILASS<br>STTTTTAAAAARPTSSHYLVRHSLCLYAASSVILALCYLTIPTVASDTPHTMRGKTRFAEGKSDKEILDH<br>LLTAARYDKRLLPPVDDADFCCGMQTPEMAKHIPDTRVPGRPQNRGSLTVNISVLLLSLASPDESSLKYE<br>VEFLLQQQWYDPRLRYANQSSYEYLNAIHHHEDIWIPDTYFIMHGDFKDPLIPMHFALRIYRNGTINYLM<br>RRHLILSCQGRLNIFPFDDPLCSFALESISYEQSAIRYVWKNDEDTLRKSPSLTTLNAYLIQNQTITCPI<br>KASWRGNYSCLKVDLIFTRDRAFYFTTVFIPGIILVTSSFITFWLEWNAVPARSMIGVTTMLNFFTTSNG<br>FRSTLPVVSNLTAMNVWDGVCMCFIYASLLEFVCVNYVGRKRPLHNVVYRPGENPVTQRLPAVLNRIGII<br>LASPLPKRESSGPNEIVACTSCAGGTSPCTHSANNGCATETCFVQVRKKEPPHPIRVAKTIDVIARITFP<br>SAYAVFLIFFFIHYKGFS</td></tr>
<tr><td colspan="3"><u>NOTE:</u> AaegL5 has updated the pHCl by identifying a possible N-terminal sequence that is extremely elongated, however this does not include a putative N-terminal signal peptide so it remains unclear whether this sequence contains the correct N-terminus.</td></tr>
<tr><td>XM_021840621</td><td>gamma-aminobutyric acid receptor subunit beta (LOC5570466), transcript variant X4</td><td>RDL (resistance to dieldrin locus)</td></tr>
<tr><td colspan="3">>Aaeg_RDL<br>MSLEIEVPHVRCPSLGVLILTLNLALFLPQTINRTPPYVLAGTGGGSMLGDVNISAILDSFSVGYDKRVRPNYGGPP<br>VEVGVTMYVLSISSLSEVKMDFTLDFYFRQFWSDPRLAYRKRPGVETLSVGSEFIKNIWVPDTFFVNEKQSYFHIAT<br>TSNEFIRIHHSGSITRSIRLTITASCPMNLQYFPMDRQLCHIEIESFGYTMRDIRYFWKDGLSSVGMSSEVELPQFR<br>VLGHRQRATEINLTTGNYSRLACEIQFVRSMGYYLIQIYIPSGLIVIISWVSFWLNRNATPARVALGVTTVLTMTTL<br>MSSTNAALPKISYVKSIDVYLGTCFVMVFASLLEYATVGYMAKRIQMRKQRFMAIQKIAEQKKQQAADANHPPPPPP<br>VSDHSHGHGHGHSHGHQHTPKQQMGSRSGTMSNVPPHNIAGSRGCSIVGPLFQEVRFKVHDPKAHSKGGTLENTING<br>GRGGGGPPGGGGGPPGGGGGGPDEESGAPQHLIHPGKDINKLLGITPSDIDKYSRIVFPVCFVCFNLMYWIIYLHVS<br>DVVADDLVLLGEEK</td></tr>
<tr><td colspan="3"><u>NOTE:</u> Sequence already published with accession number AAA68961.1.</td></tr>
</table>
